# Change in Cognition Following Ischaemic Stroke

**DOI:** 10.1002/acn3.70192

**Published:** 2025-09-22

**Authors:** Wenci Yan, Terence Quinn, Alex McConnachie, Niall Broomfield, Yun Wong, David Dickie, Kirsten Forbes, Matthew Walters, Jesse Dawson

**Affiliations:** ^1^ Department of Neurology First Affiliated Hospital of Harbin Medical University Harbin China; ^2^ School of Cardiovascular and Metabolic Health, College of Medical, Veterinary & Life Sciences, Queen Elizabeth University Hospital University of Glasgow Glasgow UK; ^3^ School of Cardiovascular and Metabolic Health, College of Medical, Veterinary & Life Sciences University of Glasgow, Glasgow Royal Infirmary Glasgow UK; ^4^ Robertson Centre for Biostatistics, School of Health and Wellbeing, College of Medical, Veterinary & Life Sciences University of Glasgow Glasgow UK; ^5^ Department of Clinical Psychology and Psychological Therapies, Norwich Medical School University of East Anglia Norwich UK; ^6^ School of Medicine, Dentistry and Nursing, College of Medical, Veterinary & Life Sciences University of Glasgow Glasgow UK; ^7^ Department of Neuroradiology, Institute of Neurological Sciences Queen Elizabeth University Hospital Glasgow UK

**Keywords:** cognitive impairment, cognitive trajectory, post‐stroke cognition, risk factor, stroke

## Abstract

**Objective:**

Cognitive decline can occur following ischaemic stroke. How cognition changes over time and associations with cognitive change are poorly understood. This study aimed to explore these issues over 2 years following ischaemic stroke.

**Methods:**

This analysis used data from the XILO‐FIST study, a clinical trial of allopurinol versus placebo in people with ischaemic stroke according to Tissue‐Based Definition. Participants underwent clinical assessment, brain MRI at baseline, and Montreal Cognitive Assessment (MoCA) at baseline, year 1 and year 2. We defined cognitive impairment as a MoCA score < 26 and cognitive change as a difference in MoCA score of 2 points or more at year 1 or year 2 after randomisation. Associations with cognitive impairment and cognitive change were assessed by univariable analysis and multiple logistic regression.

**Results:**

Three hundred and sixty participants with complete MoCA data were included. Mean age was 65.4 (SD 8.36) years, and mean baseline MoCA score was 26.4 (SD 2.7). Seventy‐seven participants had second‐year cognitive improvement. Eighty‐four had second‐year cognitive decline. After adjustment for age and education year, second‐year cognitive improvement was associated with smaller brain volume, lower albumin level, smoking and greater white‐matter hyperintensity, and second‐year cognitive decline was associated with peripheral arterial disease, higher cholesterol level, small‐vessel stroke and greater white‐matter hyperintensity.

**Interpretation:**

Cognition is dynamic following stroke, with different patterns of change. Brain reserve and vascular risk factors relate to later post‐stroke cognitive change. This complex nature of cognitive trajectory has implications for cognitive rehabilitation provision and cognitive impairment detection after stroke.

## 1 Introduction

Cognitive deficits are common after stroke, affecting as many as 1/3 of stroke survivors [1]. Cognitive impairment after stroke can be a consequence of brain damage caused by the stroke itself, the effect of concomitant risk factors such as hypertension, or progression of underlying disorders such as Alzheimer's disease. As a result, cognition after stroke is dynamic and variable. Cognitive decline, cognitive improvement and stable cognition have been described in many studies. In a recent systematic review and meta‐analysis, studies with short durations of follow up tended to show average cognitive improvement in the cohort, while those with longer durations of follow up showed average cognitive decline [2]. Additionally, studies found cognitive trajectories can show 2 distinct slopes in the first and second year after stroke [3, 4]. Predictors of cognitive change also vary and many reported studies are old or used population‐based cohorts where detailed imaging data was not available. Associations with post stroke cognitive impairment, such as demographic status, previous stroke history, stroke severity and white‐matter hyperintensity have been described [1, 2], but it is less clear how these relate to cognitive change over time.

We aimed to address some of these uncertainties using data from the XILO‐FIST trial which contains detailed data on baseline characteristics, brain magnetic resonance imaging and longitudinal assessment of cognitive function. We aimed to describe longitudinal changes in cognition over a two‐year period and identify predictors of cognitive impairment after ischaemic stroke and change over 2 years thereafter.

## 2 Methods

### 2.1 Study Design

This study is a post hoc analysis of data from the XILO‐FIST trial, which was a randomised, double‐blind, placebo‐controlled, parallel group clinical trial comparing the effect of allopurinol with placebo on white matter hyperintensity progression in people aged greater than 50 years with recent (within 1 month) ischaemic stroke according to Tissue‐Based Definition [5] which included transient ischaemic attack (TIA) with positive imaging. People with a diagnosis of dementia, cognitive impairment deemed sufficient to compromise compliance with the protocol, or who could not consent were not included. The study was approved by the NHS Research Ethics Committee (REC number 14/WS/0113). The trial protocol has been published and the main trial results have been reported [6, 7]. Reporting of this analysis followed the Strengthening the Reporting of Observational Studies in Epidemiology (STROBE) guidance [8].

### 2.2 Clinical, Cognitive and Brain Imaging Assessments

Baseline assessments were performed at the end of a 4‐week run‐in phase and included clinical assessments and brain magnetic resonance imaging (MRI). Details on clinical assessments are given in the supplement. Brain MRI was performed. Images were reviewed using definitions provided in the STRIVE guidance [9]. Two trained observers assigned a Fazekas scale score [10] and Scheltens scale score [11]. White‐matter hyperintensity (WMH) volume, intracranial volume (ICV), brain volume and cerebrospinal fluid (CSF) volume were calculated using previously reported methods [12, 13].

Cognition was evaluated at baseline, year 1 and year 2 using the Montreal Cognitive Assessment (MoCA, version 7.3) by trained assessors in a standardised fashion. We defined cognitive impairment as a MoCA score of < 26 [14]. We defined a change in cognition during year 1 as a ≥ 2 point change in MoCA score between baseline and year 1. We defined a change in cognition during year 2 as a ≥ 2 point change in MoCA score between year 1 and year 2. This threshold for cognitive change was based on the Reliability Change Index cutoff of ±1.73, which represents a clinically meaningful difference [15].

### 2.3 Statistical Analysis

Participants who completed cognitive assessments at baseline, year 1 and year 2 were included in the analyses. Participants were included regardless of randomised treatment group as allopurinol had no effect on MoCA score [7]. We described the numbers of participants who had cognitive decline, improvement, or stable cognition in year 1 and year 2 after randomisation. We analysed the difference in MoCA score at year 2 among cognitive change types at year 1 and at year 2, and among cognitive change patterns over 2 years. We analysed the difference in cognitive impairment and in patterns of cognitive change between participants having ischemic stroke and participants having TIA with positive imaging. We then described associations with cognitive impairment over year 1 and year 2 after stroke and with patterns of cognitive change. We performed unadjusted logistic regression analysis and logistic regression adjusting for age and education year on each factor. Due to the small number who had improvement in cognition in both year 1 and year 2 and decline in both year 1 and year 2, we did not perform multivariable analysis for these patterns of change. Sensitivity analysis was performed to (1) test the effect of allopurinol treatment by additional adjustment for allopurinol treatment group and (2) test the effect of missing data by repeating the analysis using the data after multiple imputation. For multiple imputation, Markov chain Monte Carlo method was used, and the number of imputations was 20. Two‐tailed *p*‐values < 0.05 was considered statistically significant. Data were analysed using SPSS 29.0.

## 3 Results

### 3.1 Baseline Data

In total, 360 participants were included in this analysis. The mean age of included participants was 65.4 (SD 8.4) years, 32% were female and 7% had TIA with positive imaging. Mean time from stroke to baseline assessments was 40.0 (SD 8.8) days. Baseline characteristics are shown in Table S1.

### 3.2 Cognition and Cognitive Change Over 2 Years

At baseline, year 1 and year 2, the mean MoCA score was 26.4 (SD 2.7), 26.8 (SD 2.9) and 26.8 (SD 2.8) respectively. The number of participants with cognitive impairment was 107 (30%) at baseline, 95 (26%) at year 1 and 94 (26%) at year 2 (Figure S1). In the first year after stroke, 123 (34%) participants had a 2 or more points increase in MoCA score, 39 (11%) participants had cognitive improvement to the max MoCA score, and 73 (20%) participants had a 2 or more points decrease. In year 2, 77 (21%) participants had a 2 or more points increase, 27 (8%) improved to the max MoCA score, and 84 (23%) participants had a 2 or more points decrease. 56% of those who had 2 or more points change in the first year had a 3 or more points change, and 61% of those having 2 or more points change in the second year had a 3 or more points change (Table S2). Across participants with cognitive improvement, stable cognition and cognitive decline in the first year after stroke, there was no significant difference in MoCA score at year 2 (*H* = 4.873, df = 2, *p* = 0.087). But across participants with different cognitive change types in the second year, there was a significant difference in MoCA score at year 2 (*H* = 90.918, df = 2, *p* < 0.001), and participants having cognitive decline in the second year had the lowest MoCA score median (Table S3).

**FIGURE 1:**
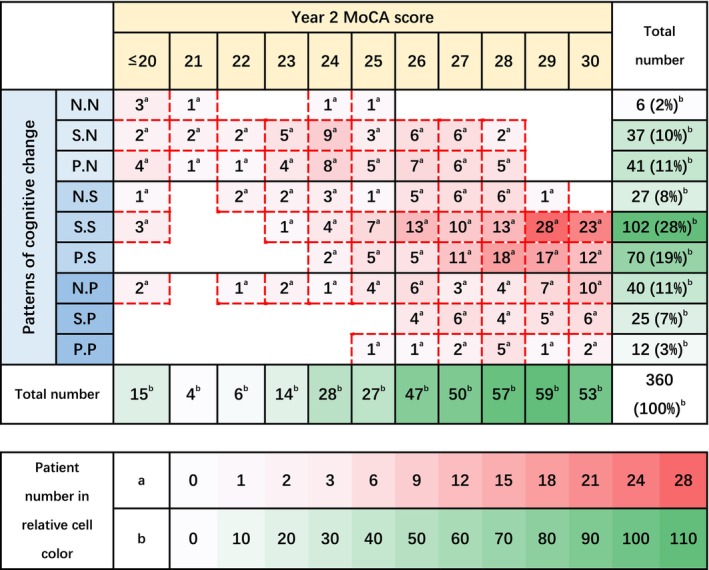
Patterns of cognitive change, and year 2 MoCA score. For patterns of cognitive change, the first letter denotes change in year 1 and the second change in year 2 (N: Negative cognitive change; S: Stable cognition; P: Positive cognitive change). For example, S.P denotes stable cognition in year 1 and a 2 point or more improvement in MoCA score in year 2.

There were nine possible patterns of cognitive change during the study and all nine were observed (Figures 1 and S2). 12 (3%) participants had improvement in cognition in year 1 and 2; 65 (18%) had stable cognition or cognitive decline in year 1 followed by cognitive improvement in year 2; 102 (28%) participants had stable cognition in both years; 70 (19%) had cognitive improvement in year 1 and stable cognition in year 2; 6 (2%) participants had cognitive decline in years 1 and 2; and 78 (22%) had stable cognition or cognitive improvement in year 1 then cognitive decline in year 2. There was a significant difference in MoCA score at year 2 (*H* = 112.630, df = 8, *p* < 0.001) among nine patterns of cognitive change. Three patterns of cognitive decline in year 2 had relatively lower MoCA score median, even in the pattern of cognitive improvement in the first year followed by cognitive decline in the second year. Among most patterns of cognitive improvement and patterns of stable cognition in year 2, MoCA score at year 2 there was no significant difference (Table S4).

Between participants having ischemic stroke and participants having TIA with positive imaging, no significant difference in cognitive impairment was found at baseline (*X* ^2^ = 0.592, df = 1, *p* = 0.441), at year 1 (*X* ^2^ = 0.277, df = 1, *p* = 0.599), or at year 2 (*X* ^2^ = 0.315, df = 1, *p* = 0.575) (Table S5). All nine patterns of cognitive change were observed in ischemic stroke participants. Except for continuous cognitive improvement, the other eight patterns of cognitive change were observed in participants having TIA with positive imaging (Figure S3). No significant difference in patterns of cognitive change was found between participants having ischemic stroke and participants having TIA with positive imaging (*p* = 0.672) (Table S6).

### 3.3 Associations With Cognitive Impairment at Year 1 and Year 2

**FIGURE 2:**
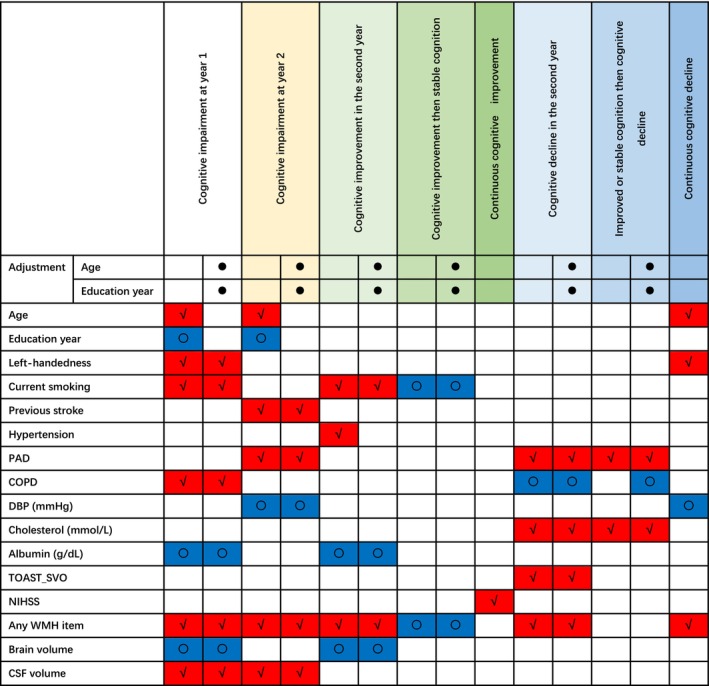
Predictors of cognitive change patterns. ‘√’: Positive association; ‘○’: Negative association. WMH item includes Fazekas' score (PVHs, DWMH and total score), Scheltens' score (PVH, WMH, BG, ITF and total score) and WMH volume/ICV. The significance level is 0.05.

At year 1 after stroke, after adjusting for age and education year, left‐handedness (OR, 2.648 [1.154, 6.075]), smoking (OR, 2.248 [1.239, 4.077]), COPD (OR, 2.677 [1.162, 6.163]), lower albumin (OR, 0.905 [0.848, 0.965]), higher Scheltens WMH (OR, 1.078 [1.018, 1.140]) and total (OR, 1.05 [1.011, 1.090]) score, higher WMH volume/ICV*100% (OR, 1.28 [1.033, 1.585]), lower brain volume (OR, 0.996 [0.994, 0.999]) and higher CSF volume (OR, 1.006 [1.001, 1.010]) were associated with higher odds of cognitive impairment. The pseudo *R* ^2^ of associated variable ranged from 0.020 to 0.054, and the variable with the highest pseudo *R* ^2^ was brain volume (Figure 2, Table S7).

At year 2 after stroke, after adjusting for age and education year, previous stroke (OR, 3.145 [1.456, 6.792]), a history of PAD (OR, 4.529 [1.769, 11.593]), lower diastolic blood pressure (DBP) (OR, 0.972 [0.950, 0.996]), higher Fazeka PVHs (OR, 1.447 [1.022, 2.051]) and total (OR, 1.24 [1.018, 1.510]) score, higher Scheltens PVH (OR, 1.231 [1.001, 1.513]), WMH (OR, 1.069 [1.010, 1.131]) and total (OR, 1.043 [1.005, 1.082]) score, higher WMH volume/ICV*100% (OR, 1.348 [1.086, 1.073]) and higher CSF volume (OR, 1.009 [1.004, 1.014]) were associated with higher odds of cognitive impairment. The pseudo *R* ^2^ of associated variables ranged from 0.030 to 0.076, and the variable with the highest pseudo *R* ^2^ was CSF volume (Figure 2, Table S8).

### 3.4 Associations With Cognitive Changes

After adjusting for age and education year, higher odds of cognitive improvement in the second year after stroke were associated with smoking (OR, 3.325 [1.805, 6.125]), lower albumin level (OR, 0.917 [0.857, 0.981]), higher Scheltens BG score (OR, 1.304 [1.081, 1.573]) and lower brain volume (OR, 0.995 [0.993, 0.998]). The pseudo *R* ^2^ of associated variables ranged from 0.020 to 0.060, and the variable with the highest pseudo *R* ^2^ was brain volume (Figure 2, Table S9).

After adjusting for age and education year, higher odds of cognitive decline in the second year were associated with a history of PAD (OR, 3.481 [1.392, 8.708]), no history of COPD (OR, 0.115 [0.015, 0.865]), small‐vessel stroke (OR, 1.783 [1.055, 3.012]), higher cholesterol level (OR, 1.429 [1.097, 1.862]), higher Fazeka PVHs score (OR, 1.505 [1.053, 2.151]) and higher WMH volume/ICV*100% (OR, 1.346 [1.083, 1.675]). The pseudo *R* ^2^ of associated variable ranged from 0.019 to 0.035, and the variable with the highest pseudo *R* ^2^ was WMH volume (Figure 2, Table S12).

The associations between baseline variables and other patterns of cognitive change that may be of interest to clinicians are shown in the supplement (cognitive improvement then stable cognition after stroke (Table S10), continuous cognitive improvement (Table S11), delayed cognitive decline (Table S13) and continuous cognitive decline (Table S14)). Allopurinol treatment had no effect on cognitive impairment or cognitive change patterns, and adjustment of the allopurinol treatment group did not affect the significance of any variables (Tables S15–S20). There was no significant difference between original data and imputed data (Table S21). All of the significance of associations were maintained using the imputed data (Tables S22–S35). The summary of predictors for patterns of cognitive change is shown in Figure 2.

## 4 Discussion

We found cognitive impairment was common early after stroke, even in a population of people aged an average of 65 years with relatively mild ischaemic stroke, who were deemed able to consent to a trial by a trial investigator and who did not have a known diagnosis of cognitive impairment on admission. Although the mean MoCA score was similar at all timepoints, cognitive change was variable between individuals. Few people exhibited continuous change in cognition across both the first and second years. The commonest pattern was stable cognition in years 1 and 2, followed by improvement in year 1 and stable cognition in year 2. Approximately 1/5 of people had cognitive improvement in the second year after stroke, and a similar number of people had cognitive decline in this period. Cognitive change types in the second year were related to cognitive condition at year 2. Measures of white matter hyperintensity burden were consistently associated with cognitive impairment at years 1 and 2 and were also typically associated with adverse patterns of change in years 1 and 2. Smaller brain volume, smoking and lower albumin level were associated with second‐year cognitive improvement. PAD and higher cholesterol level were associated with second‐year cognitive decline.

The prevalence of post‐stroke cognitive impairment ranges from 17% to 92% in different studies depending on the study criteria used, stroke subtype, time after stroke and population [16]. Our data are in keeping with the previous report, but a prevalence of 30% is high given that people with known cognitive impairment before stroke were excluded, most people had relatively mild stroke, and the average age was 65 years.

We observed spontaneous cognitive recovery in the first year after stroke in around 1/3 of participants and some had cognitive improvement to the maximum MoCA score. This early cognitive improvement is consistent with reports of studies with follow‐up for 3 months to 13 months [2, 17]. And this could be explained by brain recovery after the initial cognitive impairment due to the stroke [3, 18]. We also observed that around 1/5 of people had cognitive improvement in the second year, suggesting post‐stroke cognitive recovery may occur over a long period. We found that smaller brain volume, lower albumin level, smoking and more severe white‐matter hyperintensity were associated with cognitive impairment at year 1 and cognitive improvement at year 2, suggesting that people with these factors of worse brain reserve and cognition [19, 20] may require longer to recover from the cognitive insult caused by stroke.

In contrast, we observed that 23% of people had clinically meaningful cognitive decline in the second year after stroke. Most of them (93%) did not have cognitive decline in the first year, and most of them (83%) did not have cognitive impairment at year 1. Some vascular risk factors, like PAD, higher cholesterol level, small vessel disease stroke and greater WMH burden were associated with later and (or) delayed decline. PAD and greater WMH burden were associated with cognitive impairment in the second year. These factors may, through increasing stroke recurrence and small vessel disease burden, lead to delayed‐onset post‐stroke dementia after stroke [4, 21]. This suggests that physicians should be aware that these people may exhibit later cognitive decline after stroke and should monitor their cognition for longer than 2 years after stroke even though they have unimpaired cognition early after stroke.

We found people with COPD were more likely to have cognitive impairment early after stroke and less likely to have later or delayed cognitive decline. This may be as COPD exacerbates cerebral hypoxia [22], so they already had poorer cognition early after stroke. Additionally, we found lower DBP was associated with continuous cognitive decline and cognitive impairment at year 2. Lower DBP early after stroke increases the risk of cognitive impairment three months after stroke [23]. Long‐term low blood pressure can cause a reduction in cerebral blood flow and brain tissue metabolism, leading to white matter disturbances and cognitive impairment. Maintaining appropriate blood pressure may be beneficial to maintaining cognitive function after stroke. No significant differences were observed in cognitive impairment or in the patterns of cognitive change between participants who experienced ischaemic stroke and those who experienced TIA with positive imaging findings. No significant association was identified between the specific pattern of cognitive change and TIA with positive imaging events. These findings imply that underlying pathological processes, rather than clinical manifestations alone, may play a more prominent role in shaping cognitive trajectories following cerebral ischemic events. In this study, even the imaging variables and measures of WMH, which had the highest pseudo *R* ^2^ in most models, only explained a small proportion of cognitive impairment or cognitive change. There is still much to learn regarding the causes of cognitive issues after stroke.

There are several limitations to our analysis. The data come from a clinical trial which excluded people with pre‐stroke dementia and we excluded people with missing cognitive assessments. Participants had mild stroke, an average age lower than in unselected populations and were predominantly male. This may reduce the generalisability of the findings to a broader stroke population, particularly those with more severe cognitive deficits. In addition, the sample size was small. There were small numbers with continuous cognitive improvement or decline. We included the data of participants taking allopurinol, which may have had an effect on cognitive function [24], albeit no effect of allopurinol treatment was observed in our models [7]. Baseline assessments were performed an average of 40 days after stroke. We may therefore have missed early changes after stroke. In addition, regression to the mean and practice effects can confound longitudinal assessment of cognition. We also used MoCA score in this analysis, so changes in cognitive domains not well assessed by MoCA cannot be excluded. We did not analyse the association of factors like stroke recurrence, post‐stroke depression, medication adherence and rehabilitation therapy, which may also relate to cognitive trajectory after stroke. Strengths of our study include a well‐phenotyped population who were part of a monitored clinical trial. All assessors were trained in the use of the MoCA score, and we had repeated measures over time. We demonstrated the variety of cognitive trajectories after stroke and analysed the associated factors of different cognitive change patterns.

In summary, our findings have the following implications: (1) There are a variety of patterns of cognitive change following stroke. Later recovery and decline are both common, so ongoing cognitive assessments after stroke are needed using standardised screening tools such as MoCA. (2) For people having early cognitive impairment with worse brain reserve and cognition reserve, such as smaller brain volume, lower albumin level, smoking and greater WMH burden, longer than 1 year of cognitive rehabilitation may be reasonable given the potential delayed cognitive recovery. (3) For people with vascular risk factors, such as PAD, higher cholesterol level and greater WMH burden, there is a risk of cognitive decline in the longer term. (4) Smoking cessation, treatment of hypoalbuminemia, maintenance of appropriate blood pressure and lipid‐lowering therapy may be potential interventions to reduce post‐stroke cognitive impairment.

## Author Contributions

Wenci Yan performed the analysis and drafted the manuscript with Jesse Dawson. All authors made critical revisions to the manuscript. Yun Wong contributed to study analysis. Terence Quinn, Alex McConnachie, Niall Broomfield, Matthew Walters, David Dickie, Kirsten Forbes designed the study and collected study data. Jesse Dawson was the study Chief Investigator.

## Conflicts of Interest

The authors declare no conflicts of interest.

## Supporting information


**Supinfo S1.** Details on clinical assessments.
**Table S1:** Description of baseline measurements.
**Table S2:** Participant number of different cognitive change types in the first year and the second year.
**Table S3:** Difference in MoCA score at year 2 after stroke among cognitive change types in the first year and the second year.
**Table S4:** Difference in MoCA score at year 2 after stroke among 9 cognitive change patterns over 2 years.
**Table S5:** Difference in cognitive impairment between participants having ischemic stroke and participants having TIA with positive imaging.
**Table S6:** Difference in patterns of cognitive change between participants having ischemic stroke and participants having TIA with positive imaging.
**Table S7:** Predictors of cognitive impairment at year 1 after stroke.
**Table S8:** Predictors of cognitive impairment at year 2 after stroke.
**Table S9:** Predictors of cognitive improvement in the second year after stroke.
**Table S10:** Predictors of cognitive improvement then stable cognition after stroke.
**Table S11:** Predictors of continuous cognitive improvement over 2 years after stroke.
**Table S12:** Predictors of cognitive decline in the second year after stroke.
**Table S13:** Predictors of delayed cognitive decline after stroke.
**Table S14:** Predictors of continuous cognitive decline over 2 years after stroke.
**Table S15:** Predictors of cognitive impairment in year 1 after stroke (adjusting for allopurinol treatment group).
**Table S16:** Predictors of cognitive impairment in year 2 after stroke (adjusting for allopurinol treatment group).
**Table S17:** Predictors of cognitive improvement in the second year after stroke (adjusting for allopurinol treatment group).
**Table S18:** Predictors of cognitive improvement then stable cognition after stroke (adjusting for allopurinol treatment group).
**Table S19:** Predictors of cognitive decline in the second year after stroke (adjusting for allopurinol treatment group).
**Table S20:** Predictors of delayed cognitive decline after stroke (adjusting for allopurinol treatment group).
**Table S21:** Difference between original data and imputed data.
**Table S22:** Predictors of cognitive impairment in year 1 after stroke (multiple imputed data).
**Table S23:** Predictors of cognitive impairment in year 2 after stroke (multiple imputed data).
**Table S24:** Predictors of cognitive improvement in the second year after stroke (multiple imputed data).
**Table S25:** Predictors of cognitive improvement then stable cognition after stroke (multiple imputed data).
**Table S26:** Predictors of continuous cognitive improvement over 2 years after stroke (multiple imputed data).
**Table S27:** Predictors of cognitive decline in the second year after stroke (multiple imputed data).
**Table S28:** Predictors of delayed cognitive decline in the second year after stroke (multiple imputed data).
**Table S29:** Predictors of continuous cognitive decline over 2 years after stroke (multiple imputed data).
**Table S30:** Predictors of cognitive impairment in year 1 after stroke (adjusting for allopurinol treatment group using multiple imputed data).
**Table S31:** Predictors of cognitive impairment in year 2 after stroke (adjusting for allopurinol treatment group using multiple imputed data).
**Table S32:** Predictors of cognitive improvement in the second year after stroke (adjusting for allopurinol treatment group using multiple imputed data).
**Table S33:** Predictors of cognitive improvement then stable cognition after stroke (adjusting for allopurinol treatment group using multiple imputed data).
**Table S34:** Predictors of cognitive decline in the second year after stroke (adjusting for allopurinol treatment group using multiple imputed data).
**Table S35:** Predictors of delayed cognitive decline after stroke (adjusting for allopurinol treatment group using multiple imputed data).
**Figure S1:** Cognition and cognitive change over 2 years.
**Figure S2:** Cognitive trajectory over 2 years.
**Figure S3:** Patterns of cognitive change over 2 years in participants having ischemic stroke and in participants having TIA with positive imaging.

## Data Availability

Study data will be shared with academic investigators or health care professionals following review and approval of a proposal and subject to a data sharing agreement (contact: jesse.dawson@glasgow.ac.uk).
