# Supplementary material for: Change in Cognition Following Ischaemic Stroke
Source: Ann Clin Transl Neurol. 2025 Sep 22;13(1):14–20. doi: 10.1002/acn3.70192 (PMC12790154; doi:10.1002/acn3.70192)
Supplement: Supplementary file 1 — Supinfo S1. Details on clinical assessments. Table S1: Description of baseline measurements. Table S2: Participant number of different cognitive change types in the first year and the second year. Table S3: Difference in MoCA score at year 2 after stroke among cognitive change types in the first year and the second year. Table S4: Difference in MoCA score at year 2 after stroke among 9 cognitive change patterns over 2 years. Table S5: Difference in cognitive impairment between participants having ischemic stroke and participants having TIA with positive imaging. Table S6: Difference in patterns of cognitive change between participants having ischemic stroke and participants having TIA with positive imaging. Table S7: Predictors of cognitive impairment at year 1 after stroke. Table S8: Predictors of cognitive impairment at year 2 after stroke. Table S9: Predictors of cognitive improvement in the second year after stroke. Table S10: Predictors of cognitive improvement then stable cognition after stroke. Table S11: Predictors of continuous cognitive improvement over 2 years after stroke. Table S12: Predictors of cognitive decline in the second year after stroke. Table S13: Predictors of delayed cognitive decline after stroke. Table S14: Predictors of continuous cognitive decline over 2 years after stroke. Table S15: Predictors of cognitive impairment in year 1 after stroke (adjusting for allopurinol treatment group). Table S16: Predictors of cognitive impairment in year 2 after stroke (adjusting for allopurinol treatment group). Table S17: Predictors of cognitive improvement in the second year after stroke (adjusting for allopurinol treatment group). Table S18: Predictors of cognitive improvement then stable cognition after stroke (adjusting for allopurinol treatment group). Table S19: Predictors of cognitive decline in the second year after stroke (adjusting for allopurinol treatment group). Table S20: Predictors of delayed cognitive decline after stroke (adjusting fo [file ACN3-13-14-s001.docx]

**Supplemental Material**

[Details on clinical assessments. 3](#_Toc202705762)

[Table S1: Description of baseline measurements. 3](#_Toc202705763)

[Table S2: Participant number of different cognitive change types in the first year and the second year. 4](#_Toc202705764)

[Table S3: Difference in MoCA score at year 2 after stroke among cognitive change types in the first year and the second year. 5](#_Toc202705765)

[Table S4: Difference in MoCA score at year 2 after stroke among 9 cognitive change patterns over 2 years. 5](#_Toc202705766)

[Table S5: Difference in cognitive impairment between participants having ischemic stroke and participants having TIA with positive imaging. 6](#_Toc202705767)

[Table S6: Difference in patterns of cognitive change between participants having ischemic stroke and participants having TIA with positive imaging. 7](#_Toc202705768)

[Table S7: Predictors of cognitive impairment at year 1 after stroke. 7](#_Toc202705769)

[Table S8: Predictors of cognitive impairment at year 2 after stroke. 8](#_Toc202705770)

[Table S9: Predictors of cognitive improvement in the second year after stroke. 9](#_Toc202705771)

[Table S10: Predictors of cognitive improvement then stable cognition after stroke. 10](#_Toc202705772)

[Table S11: Predictors of continuous cognitive improvement over 2 years after stroke 12](#_Toc202705773)

[Table S12: Predictors of cognitive decline in the second year after stroke. 13](#_Toc202705774)

[Table S13: Predictors of delayed cognitive decline after stroke. 14](#_Toc202705775)

[Table S14: Predictors of continuous cognitive decline over 2 years after stroke. 15](#_Toc202705776)

[Table S15: Predictors of cognitive impairment in year 1 after stroke (adjusting for allopurinol treatment group). 16](#_Toc202705777)

[Table S16: Predictors of cognitive impairment in year 2 after stroke (adjusting for allopurinol treatment group). 17](#_Toc202705778)

[Table S17: Predictors of cognitive improvement in the second year after stroke (adjusting for allopurinol treatment group). 18](#_Toc202705779)

[Table S18: Predictors of cognitive improvement then stable cognition after stroke (adjusting for allopurinol treatment group). 20](#_Toc202705780)

[Table S19: Predictors of cognitive decline in the second year after stroke (adjusting for allopurinol treatment group). 21](#_Toc202705781)

[Table S20: Predictors of delayed cognitive decline after stroke (adjusting for allopurinol treatment group). 22](#_Toc202705782)

[Table S21: Difference between original data and imputed data. 23](#_Toc202705783)

[Table S22: Predictors of cognitive impairment in year 1 after stroke (multiple imputed data). 26](#_Toc202705784)

[Table S23: Predictors of cognitive impairment in year 2 after stroke (multiple imputed data). 27](#_Toc202705785)

[Table S24: Predictors of cognitive improvement in the second year after stroke (multiple imputed data). 28](#_Toc202705786)

[Table S25: Predictors of cognitive improvement then stable cognition after stroke (multiple imputed data). 29](#_Toc202705787)

[Table S26: Predictors of continuous cognitive improvement over 2 years after stroke (multiple imputed data). 31](#_Toc202705788)

[Table S27: Predictors of cognitive decline in the second year after stroke (multiple imputed data). 32](#_Toc202705789)

[Table S28: Predictors of delayed cognitive decline in the second year after stroke (multiple imputed data). 33](#_Toc202705790)

[Table S29: Predictors of continuous cognitive decline over 2 years after stroke (multiple imputed data). 34](#_Toc202705791)

[Table S30: Predictors of cognitive impairment in year 1 after stroke (adjusting for allopurinol treatment group using multiple imputed data). 35](#_Toc202705792)

[Table S31: Predictors of cognitive impairment in year 2 after stroke (adjusting for allopurinol treatment group using multiple imputed data). 37](#_Toc202705793)

[Table S32: Predictors of cognitive improvement in the second year after stroke (adjusting for allopurinol treatment group using multiple imputed data). 38](#_Toc202705794)

[Table S33: Predictors of cognitive improvement then stable cognition after stroke (adjusting for allopurinol treatment group using multiple imputed data). 39](#_Toc202705795)

[Table S34: Predictors of cognitive decline in the second year after stroke (adjusting for allopurinol treatment group using multiple imputed data). 40](#_Toc202705796)

[Table S35: Predictors of delayed cognitive decline after stroke (adjusting for allopurinol treatment group using multiple imputed data). 42](#_Toc202705797)

[Figure S1: Cognition and cognitive change over 2 years. 43](#_Toc202705798)

[Figure S2: Cognitive trajectory over 2 years. 44](#_Toc202705799)

[Figure S3: Patterns of cognitive change over 2 years in participants having ischemic stroke and in participants having TIA with positive imaging. 45](#_Toc202705800)

## Details on clinical assessments.

Clinical assessments included demographic characteristics (age, education year, gender and dominant hand), health behaviours (current smoking or others, alcohol consumption or others), comorbidities (previous stroke, peripheral arterial disease (PAD), myocardial infarction, carotid artery disease, hypertension, diabetes and chronic obstructive pulmonary disease (COPD)), stroke features (stroke or transient ischaemic attack, TOAST stroke subtype (Due to small-vessel occlusion (SVO) dominant in our sample, dichotomous variable of small-vessel occlusion or others was used in the analysis.), NIH stroke score (NIHSS) and modified Rankin scale (mRS)), physiological measurements (mean of 2 brachial blood pressure measurements and BMI) and blood tests (haemoglobin, cholesterol, albumin, creatinine and eGFR).

## Table S1: Description of baseline measurements.

| **Variable** | **N** | **Missingness** | **Mean/N** | **SD/ %** |
| --- | --- | --- | --- | --- |
| **Time from stroke onset to baseline assessments (days)** | 360 | 0% | 40.0 | 8.8 |
| **Age (years)** | 360 | 0% | 65.4 | 8.36 |
| **Education year** | 360 | 0% | 12.4 | 2.72 |
| **Female** | 360 | 0% | 114 | 32% |
| **Left-handed** | 360 | 0% | 26 | 7% |
| **Smoker** | 360 | 0% | 70 | 19% |
| **Alcohol use** | 360 | 0% | 258 | 72% |
| **Myocardial infarction** | 360 | 0% | 32 | 9% |
| **Previous stroke** | 360 | 0% | 31 | 9% |
| **Peripheral arterial disease** | 360 | 0% | 20 | 6% |
| **Carotid artery disease** | 360 | 0% | 34 | 9% |
| **Hypertension** | 360 | 0% | 181 | 50% |
| **Diabetes** | 360 | 0% | 72 | 20% |
| **COPD** | 360 | 0% | 26 | 7% |
| **Event type (TIA with positive imaging)** | 360 | 0% | 26 | 7% |
| **TOAST SVO subtype** | 360 | 0% | 99 | 28% |
| **NIHSS** | 360 | 0% | 1.3 | 1.69 |
| **mRS** | 360 | 0% | 1.3 | 0.99 |
| **SBP (mmHg)** | 360 | 0% | 136.1 | 17.03 |
| **DBP (mmHg)** | 360 | 0% | 79.2 | 10.63 |
| **BMI (km/m2)** | 358 | 1% | 28.3 | 5.1 |
| **Haemoglobin (g/dL)** | 360 | 0% | 14.3 | 1.44 |
| **Cholesterol (mmol/L)** | 346 | 4% | 3.9 | 0.94 |
| **Albumin (g/dL)** | 360 | 0% | 40.2 | 3.98 |
| **Creatinine (μmol/L)** | 360 | 0% | 79.7 | 18.32 |
| **eGFR (mL/min)** | 360 | 0% | 86.4 | 19.75 |
| **Fazeka PVHs score** | 360 | 0% | 1.4 | 0.67 |
| **Fazeka DWMH score** | 360 | 0% | 1.3 | 0.65 |
| **Fazeka total score** | 360 | 0% | 2.7 | 1.18 |
| **Scheltens PVH score** | 360 | 0% | 3.7 | 1.15 |
| **Scheltens WMH score** | 360 | 0% | 6.6 | 4.33 |
| **Scheltens BG score** | 360 | 0% | 0.9 | 1.25 |
| **Scheltens ITF score** | 360 | 0% | 1.3 | 1.56 |
| **Scheltens total score** | 360 | 0% | 12.6 | 6.54 |
| **WMH volume/ICV*100%** | 354 | 2% | 1.2 | 0.01 |
| **Brain volume (cm^3^)** | 348 | 3% | 1061.6 | 108.86 |
| **CSF volume (cm^3^)** | 348 | 3% | 308.2 | 58.51 |
| **Trial intervention (allopurinol)** | 360 | 0% | 173 | 48% |

**Table S1: Description of baseline measurements.**

NIHSS: NIH stroke score; mRS: modified Rankin scale; SBP: systolic blood pressure; DBP: diastolic blood pressure; COPD: chronic obstructive pulmonary disease; PAD: Peripheral arterial disease; SVO: small-vessel occlusion; PVH: periventricular hyperintensity; DWMH: deep white-matter hyperintensity; WMH: white-matter hyperintensity; BG: basal ganglia; ITF: infra-tentorial foci; ICV: intracranial volume; CSF: cerebrospinal fluid.

## Table S2: Participant number of different cognitive change types in the first year and the second year.

| **Subject number, N (%)** | | **Year 2** | | | |
| --- | --- | --- | --- | --- | --- |
|  |  | **N** | **S** | **P** | **Total** |
| **Year 1** | **N** | 6 (2%) | 27 (8%) | 40 (11%) | 73 (20%) |
|  | **S** | 37 (10%) | 102 (28%) | 25 (7%) | 164 (46%) |
|  | **P** | 41 (11%) | 70 (19%) | 12 (3%) | 123 (34%) |
|  | **Total** | 84 (23%) | 199 (55%) | 77 (21%) | 360 (100%) |
| **Subject number, N (%)** | | **Year 2** | | | |
|  |  | **N2** | **S2** | **P2** | **Total** |
| **Year 1** | **N2** | 1 (0.3%) | 17 (5%) | 22 (6%) | 40 (11%) |
|  | **S2** | 31 (9%) | 199 (55%) | 20 (6%) | 250 (69%) |
|  | **P2** | 21 (6%) | 45 (13%) | 4 (1%) | 70 (19%) |
|  | **Total** | 53 (15%) | 261 (73%) | 46 (13%) | 360 (100%) |

**Table S2: Subjects number of different cognitive change types** **in the first year and the second year.**

N: Negative cognitive change group (2 or more MoCA score decrease); S: Stable cognition group (cognitive change between -1 to 1 MoCA); P: Positive cognitive change group: (2 or more MoCA score increase); N2: Negative cognitive change group 2 (3 or more MoCA score decrease); S2: Stable cognition group 2 (cognitive change between -2 to 2 MoCA); P2: Positive cognitive change group 2: (3 or more MoCA score increase).

## Table S3: Difference in MoCA score at year 2 after stroke among cognitive change types in the first year and the second year.

| **Year** | **Group** | **Number** | **Mean** | **SD** | **Median** | **IQR** | ***H* value** | ***p* value** | **Pairwise comparisons *p* value (a)** | | | |
| --- | --- | --- | --- | --- | --- | --- | --- | --- | --- | --- | --- | --- |
|  |  |  |  |  |  |  |  |  | **Groups** | **N** | **S** | **P** |
| **Year 1** | **N** | 73 | 26.0 | 3.3 | 27 | 5 | 4.873 | 0.087 | **N** | - | 0.082 | 0.450 |
|  | **S** | 164 | 27.0 | 2.8 | 27 | 3 |  |  | **S** | 0.082 | - | 1.000 |
|  | **P** | 123 | 26.9 | 2.4 | 27 | 4 |  |  | **P** | 0.450 | 1.000 | - |
| **Year 2** | **N** | 84 | 24.2 | 2.8 | 24 | 3 | 90.918 | **<0.001*** | **N** | - | **<0.001*** | **<0.001*** |
|  | **S** | 199 | 27.5 | 2.3 | 28 | 3 |  |  | **S** | **<0.001*** | - | 1.000 |
|  | **P** | 77 | 27.5 | 2.4 | 28 | 3 |  |  | **P** | **<0.001*** | 1.000 | - |

**Table S3:** **Difference in MoCA score at year 2 after stroke among cognitive change types in the first year and second year.**

Kruskal-Wallis test with Dunn’s test as post hoc test. (a): Significance values have been adjusted by the Bonferroni correction for multiple tests. N: Negative cognitive change group (2 or more MoCA score decrease); S: Stable cognition group (cognitive change between -1 to 1 MoCA); P: Positive cognitive change group: (2 or more MoCA score increase)*: *p<*0.05.

## Table S4: Difference in MoCA score at year 2 after stroke among 9 cognitive change patterns over 2 years.

| **Cognitive change patterns** | **Number** | **Mean** | **SD** | **Median** | **IQR** | ***H* value** | | ***p* value** | |
| --- | --- | --- | --- | --- | --- | --- | --- | --- | --- |
| **N.N** | 6 | 20.5 | 3.4 | 20 | 7 | 112.630 | | **<0.001*** | |
| **S.N** | 37 | 24.3 | 2.7 | 24 | 3 |  |  |  |  |
| **P.N** | 41 | 24.7 | 2.5 | 25 | 4 |  |  |  |  |
| **N.S** | 27 | 25.7 | 2.5 | 26 | 4 |  | |  | |
| **S.S** | 102 | 27.7 | 2.4 | 29 | 3 |  |  |  |  |
| **P.S** | 70 | 28.0 | 1.6 | 28 | 2 |  |  |  |  |
| **N.P** | 40 | 27.0 | 3.0 | 28 | 5 |  | |  | |
| **S.P** | 25 | 28.1 | 1.5 | 28 | 3 |  |  |  |  |
| **P.P** | 12 | 27.8 | 1.5 | 28 | 2 |  |  |  |  |
| **Pairwise comparisons *p* value (a)** | | | | | | | | | |
| **Cognitive change patterns** | **N.N** | **S.N** | **P.N** | **N.S** | **S.S** | **P.S** | **N.P** | **S.P** | **P.P** |
| **N.N** | - | 1.000 | 1.000 | 0.949 | **<0.001*** | **<0.001*** | **0.007*** | **0.001*** | **0.010*** |
| **S.N** | 1.000 | - | 1.000 | 1.000 | 1.000 | **<0.001*** | **<0.001*** | **<0.001*** | **0.005*** |
| **P.N** | 1.000 | 1.000 | - | 1.000 | **<0.001*** | **<0.001*** | **0.001*** | **<0.001*** | **0.018*** |
| **N.S** | 0.949 | 1.000 | 1.000 | - | **0.003*** | **0.003*** | 0.406 | **0.021*** | 0.702 |
| **S.S** | **<0.001*** | **<0.001*** | **<0.001*** | **0.003*** | - | 1.000 | 1.000 | 1.000 | 1.000 |
| **P.S** | **<0.001*** | **<0.001*** | **<0.001*** | **0.003*** | 1.000 | - | 1.000 | 1.000 | 1.000 |
| **N.P** | **0.007*** | **<0.001*** | **0.001*** | 0.406 | 1.000 | 1.000 | - | 1.000 | 1.000 |
| **S.P** | **0.001*** | **<0.001*** | **<0.001*** | **0.021*** | **<0.001*** | 1.000 | 1.000 | - | 1.000 |
| **P.P** | **0.010*** | **0.005*** | **0.018*** | 0.702 | 1.000 | 1.000 | 1.000 | 1.000 | - |

**Table S4:** **Difference in MoCA score at year 2 after stroke among 9 cognitive change patterns over 2 years.**

Kruskal-Wallis test with Dunn’s test as post hoc test. (a): Significance values have been adjusted by the Bonferroni correction for multiple tests. The first letter denotes change in year 1 and the second change in year 2 (N: Negative cognitive change; S: Stable cognition; P: Positive cognitive change). For example, S.P denotes stable cognition in year 1 and a 2 point or more improvement in MoCA score in year 2. *: *p<*0.05.

## Table S5: Difference in cognitive impairment between participants having ischemic stroke and participants having TIA with positive imaging.

| **Subject number, N (%)** | | **Cognitive condition** | | **Total** | ***X*2** | ***p* value** |
| --- | --- | --- | --- | --- | --- | --- |
|  |  | **Cognitive impairment** | **Cognitive non-impairment** |  |  |  |
| **Baseline** | **Stroke** | 101 (28%) | 233 (65%) | 334 (93%) | 0.592 | 0.441 |
|  | **TIA with positive imaging** | 6 (2%) | 20 (6%) | 26 (7%) |  |  |
|  | **Total** | 107 (30%) | 253 (70%) | 360 (100%) |  |  |
| **Year 1** | **Stroke** | 87 (24%) | 247 (69%) | 334 (93%) | 0.277 | 0.599 |
|  | **TIA with positive imaging** | 8 (2%) | 18 (5%) | 26 (7%) |  |  |
|  | **Total** | 95 (26%) | 265 (74%) | 360 (100%) |  |  |
| **Year 2** | **Stroke** | 86 (24%) | 248 (69%) | 334 (93%) | 0.315 | 0.575 |
|  | **TIA with positive imaging** | 8 (2%) | 18 (5%) | 26 (7%) |  |  |
|  | **Total** | 94 (26%) | 266 (74%) | 360 (100%) |  |  |

**Table S5: Difference in cognitive impairment between participants having ischemic stroke and participants having TIA with positive imaging.**

Chi-Square.

## Table S6: Difference in patterns of cognitive change between participants having ischemic stroke and participants having TIA with positive imaging.

| **Subject number, N (%)** | **Patterns of cognitive change** | | | | | | | | | **Total** | **p value** |
| --- | --- | --- | --- | --- | --- | --- | --- | --- | --- | --- | --- |
|  | **N.N** | **S.N** | **P.N** | **N.S** | **S.S** | **P.S** | **N.P** | **S.P** | **P.P** |  |  |
| **Stroke** | 5  (1%) | 35  (10%) | 39  (11%) | 25  (7%) | 91  (25%) | 67  (19%) | 36  (10%) | 24  (7%) | 12  (3%) | 334  (93%) | 0.672 |
| **TIA with positive imaging** | 1  (0%) | 2  (1%) | 2  (1%) | 2  (1%) | 11  (3%) | 3  (1%) | 4  (1%) | 1  (0%) | 0  (0%) | 26  (7%) |  |
| **Total** | 6  (2%) | 37  (10%) | 41  (11%) | 27  (8%) | 102  (28%) | 70  (19%) | 40  (11%) | 25  (7%) | 12  (3%) | 360  (100%) |  |

**Table S6: Difference in patterns of cognitive change between participants of ischemic stroke and participants of TIA with positive imaging.**

Fisher's exact test. The first letter denotes change in year 1 and the second change in year 2 (N: Negative cognitive change; S: Stable cognition; P: Positive cognitive change). For example, S.P denotes stable cognition in year 1 and a 2 point or more improvement in MoCA score in year 2.

## Table S7: Predictors of cognitive impairment at year 1 after stroke.

|  | **Model 1** | | | |  | **Model 2** | | | |
| --- | --- | --- | --- | --- | --- | --- | --- | --- | --- |
| **Variable** | **Pseudo R^2^** | **OR** | **95% CI** | ***p* value** |  | **Pseudo R^2^** | **OR** | **95% CI** | ***p* value** |
| **Age** | 0.038 | 1.046 | (1.016, 1.076) | **0.002*** |  |  |  |  |  |
| **Education year** | 0.022 | 0.896 | (0.814, 0.987) | **0.026*** |  |  |  |  |  |
| **Female** | 0.003 | 0.812 | (0.486, 1.359) | 0.428 |  | 0.060 | 0.833 | (0.493, 1.409) | 0.497 |
| **Left-handedness** | 0.020 | 2.592 | (1.153, 5.827) | **0.021*** |  | 0.078 | 2.648 | (1.154, 6.075) | **0.022*** |
| **Smoker** | 0.020 | 1.893 | (1.086, 3.297) | **0.024*** |  | 0.085 | 2.248 | (1.239, 4.077) | **0.008*** |
| **Alcohol use** | 0.005 | 0.756 | (0.455, 1.255) | 0.279 |  | 0.061 | 0.794 | (0.473, 1.333) | 0.384 |
| **Myocardial infarction** | <0.001 | 1.101 | (0.490, 2.473) | 0.815 |  | 0.059 | 1.129 | (0.496, 2.569) | 0.772 |
| **Previous stroke** | 0.010 | 1.872 | (0.872, 4.020) | 0.108 |  | 0.070 | 2.000 | (0.911, 4.390) | 0.084 |
| **PAD** | 0.003 | 1.542 | (0.596, 3.988) | 0.372 |  | 0.061 | 1.436 | (0.548, 3.764) | 0.462 |
| **Carotid artery disease** | <0.001 | 1.005 | (0.451, 2.238) | 0.991 |  | 0.059 | 0.957 | (0.424, 2.159) | 0.915 |
| **Hypertension** | 0.009 | 1.431 | (0.893, 2.295) | 0.137 |  | 0.061 | 1.237 | (0.760, 2.011) | 0.392 |
| **Diabetes** | 0.009 | 1.528 | (0.874, 2.671) | 0.137 |  | 0.063 | 1.393 | (0.784, 2.476) | 0.258 |
| **COPD** | 0.020 | 2.592 | (1.153, 5.827) | **0.021*** |  | 0.078 | 2.677 | (1.162, 6.163) | **0.021*** |
| **Event type (TIA with positive imaging)** | 0.001 | 1.262 | (0.530, 3.006) | 0.599 |  | 0.059 | 1.134 | (0.470, 2.737) | 0.780 |
| **TOAST_SVO** | <0.001 | 1.064 | (0.632, 1.793) | 0.815 |  | 0.059 | 1.009 | (0.592, 1.719) | 0.974 |
| **NIHSS** | 0.002 | 1.055 | (0.923, 1.206) | 0.431 |  | 0.062 | 1.071 | (0.931, 1.231) | 0.336 |
| **mRS** | 0.008 | 1.183 | (0.935, 1.496) | 0.162 |  | 0.067 | 1.194 | (0.938, 1.520) | 0.149 |
| **SBP (mmHg)** | 0.007 | 1.009 | (0.995, 1.023) | 0.195 |  | 0.062 | 1.007 | (0.993, 1.021) | 0.319 |
| **DBP (mmHg)** | 0.002 | 0.991 | (0.970, 1.014) | 0.449 |  | 0.059 | 0.999 | (0.977, 1.022) | 0.958 |
| **BMI (km/m2)** | 0.007 | 1.031 | (0.985, 1.079) | 0.190 |  | 0.070 | 1.038 | (0.990, 1.088) | 0.121 |
| **Haemoglobin (g/dL)** | 0.009 | 0.886 | (0.753, 1.043) | 0.146 |  | 0.063 | 0.910 | (0.768, 1.077) | 0.273 |
| **Cholesterol (mmol/L)** | 0.006 | 0.854 | (0.656, 1.112) | 0.241 |  | 0.057 | 0.893 | (0.679, 1.173) | 0.414 |
| **Albumin (g/dL)** | 0.043 | 0.903 | (0.848, 0.962) | **0.002*** |  | 0.096 | 0.905 | (0.848, 0.965) | **0.002*** |
| **Creatinine (μmol/L)** | 0.009 | 1.009 | (0.997, 1.022) | 0.139 |  | 0.062 | 1.006 | (0.994, 1.019) | 0.332 |
| **eGFR (mL/min)** | 0.005 | 0.993 | (0.982, 1.006) | 0.287 |  | 0.059 | 0.998 | (0.985, 1.011) | 0.767 |
| **Fazeka PVHs score** | 0.020 | 1.459 | (1.047, 2.033) | **0.026*** |  | 0.065 | 1.260 | (0.887, 1.791) | 0.197 |
| **Fazeka DWMH score** | 0.022 | 1.515 | (1.068, 2.148) | **0.020*** |  | 0.069 | 1.348 | (0.942, 1.928) | 0.102 |
| **Fazeka Total score** | 0.025 | 1.278 | (1.058, 1.545) | **0.011*** |  | 0.069 | 1.179 | (0.967, 1.436) | 0.103 |
| **Scheltens PVH score** | 0.019 | 1.243 | (1.021, 1.514) | **0.031*** |  | 0.064 | 1.136 | (0.924, 1.398) | 0.227 |
| **Scheltens WMH score** | 0.051 | 1.103 | (1.045, 1.165) | **<0.001*** |  | 0.084 | 1.078 | (1.018, 1.140) | **0.010*** |
| **Scheltens BG score** | 0.009 | 1.151 | (0.963, 1.376) | 0.121 |  | 0.062 | 1.098 | (0.913, 1.320) | 0.322 |
| **Scheltens ITF score** | 0.022 | 1.186 | (1.028, 1.369) | **0.019*** |  | 0.070 | 1.140 | (0.985, 1.320) | 0.080 |
| **Scheltens total score** | 0.052 | 1.067 | (1.030, 1.106) | **<0.001*** |  | 0.084 | 1.050 | (1.011, 1.090) | **0.011*** |
| **WMH volume/ICV*100%** | 0.046 | 1.413 | (1.152, 1.733) | **0.001*** |  | 0.086 | 1.280 | (1.033, 1.585) | **0.024*** |
| **Brain volume** | 0.054 | 0.996 | (0.993, 0.998) | **0.001*** |  | 0.095 | 0.996 | (0.994, 0.999) | **0.005*** |
| **CSF volume** | 0.041 | 1.007 | (1.002, 1.011) | **0.002*** |  | 0.085 | 1.006 | (1.001, 1.010) | **0.015*** |
| **Trial intervention (allopurinol)** | 0.007 | 0.722 | (0.450, 1.159) | 0.177 |  | 0.066 | 0.703 | (0.434, 1.141) | 0.154 |

**Table S7:** **Predictors of cognitive impairment at year 1 after stroke.**

Model 1: Logistic regression with no adjustment; Model 2: Logistic regression adjusting for age and education year. The reference group was participants having not cognitive impairment. *: *p<*0.05.

## Table S8: Predictors of cognitive impairment at year 2 after stroke.

|  | **Model 1** | | | |  | **Model 2** | | | |
| --- | --- | --- | --- | --- | --- | --- | --- | --- | --- |
| **Variable** | **Pseudo R^2^** | **OR** | **95% CI** | ***p* value** |  | **Pseudo R^2^** | **OR** | **95% CI** | ***p* value** |
| **Age** | 0.026 | 1.037 | (1.008, 1.067) | **0.012*** |  |  |  |  |  |
| **Education year** | 0.026 | 0.886 | (0.803, 0.977) | **0.015*** |  |  |  |  |  |
| **Female** | 0.009 | 0.670 | (0.395, 1.138) | 0.138 |  | 0.058 | 0.689 | (0.402, 1.179) | 0.174 |
| **Left-handedness** | 0.014 | 2.218 | (0.980, 5.018) | 0.056 |  | 0.064 | 2.259 | (0.981, 5.204) | 0.056 |
| **Smoker** | 0.003 | 1.274 | (0.716, 2.266) | 0.410 |  | 0.054 | 1.363 | (0.744, 2.494) | 0.316 |
| **Alcohol use** | <0.001 | 1.046 | (0.619, 1.767) | 0.866 |  | 0.051 | 1.104 | (0.648, 1.882) | 0.716 |
| **Myocardial infarction** | 0.005 | 1.546 | (0.715, 3.342) | 0.268 |  | 0.055 | 1.581 | (0.723, 3.458) | 0.252 |
| **Previous stroke** | 0.031 | 2.967 | (1.404, 6.271) | **0.004*** |  | 0.082 | 3.145 | (1.456, 6.792) | **0.004*** |
| **PAD** | 0.044 | 4.720 | (1.865, 11.943) | **0.001*** |  | 0.089 | 4.529 | (1.769, 11.593) | **0.002*** |
| **Carotid artery disease** | 0.011 | 1.872 | (0.897, 3.908) | 0.095 |  | 0.059 | 1.807 | (0.856, 3.817) | 0.121 |
| **Hypertension** | 0.011 | 1.478 | (0.919, 2.375) | 0.107 |  | 0.055 | 1.303 | (0.800, 2.120) | 0.287 |
| **Diabetes** | 0.009 | 1.557 | (0.890, 2.724) | 0.121 |  | 0.056 | 1.411 | (0.795, 2.506) | 0.239 |
| **COPD** | 0.001 | 1.282 | (0.538, 3.054) | 0.575 |  | 0.051 | 1.251 | (0.517, 3.027) | 0.620 |
| **Event type (TIA with positive imaging)** | 0.001 | 1.282 | (0.538, 3.054) | 0.575 |  | 0.051 | 1.155 | (0.479, 2.781) | 0.748 |
| **TOAST_SVO** | 0.011 | 1.533 | (0.922, 2.549) | 0.100 |  | 0.059 | 1.491 | (0.888, 2.503) | 0.131 |
| **NIHSS** | <0.001 | 1.017 | (0.887, 1.167) | 0.806 |  | 0.051 | 1.023 | (0.886, 1.180) | 0.758 |
| **mRS** | 0.004 | 1.123 | (0.887, 1.422) | 0.336 |  | 0.054 | 1.124 | (0.883, 1.430) | 0.343 |
| **SBP (mmHg)** | <0.001 | 0.998 | (0.985, 1.012) | 0.825 |  | 0.051 | 0.996 | (0.982, 1.011) | 0.617 |
| **DBP (mmHg)** | 0.034 | 0.967 | (0.945, 0.990) | **0.005*** |  | 0.072 | 0.972 | (0.950, 0.996) | **0.020*** |
| **BMI (km/m2)** | 0.010 | 1.038 | (0.992, 1.086) | 0.108 |  | 0.064 | 1.042 | (0.995, 1.093) | 0.083 |
| **Haemoglobin (g/dL)** | 0.002 | 0.949 | (0.806, 1.118) | 0.532 |  | 0.051 | 0.969 | (0.818, 1.147) | 0.711 |
| **Cholesterol (mmol/L)** | <0.001 | 1.006 | (0.779, 1.299) | 0.963 |  | 0.046 | 1.044 | (0.801, 1.360) | 0.749 |
| **Albumin (g/dL)** | 0.001 | 0.983 | (0.926, 1.043) | 0.570 |  | 0.051 | 0.987 | (0.928, 1.049) | 0.674 |
| **Creatinine (μmol/L)** | 0.008 | 1.009 | (0.997, 1.022) | 0.145 |  | 0.055 | 1.007 | (0.994, 1.020) | 0.290 |
| **eGFR (mL/min)** | 0.001 | 0.997 | (0.986, 1.009) | 0.669 |  | 0.050 | 1.001 | (0.989, 1.014) | 0.842 |
| **Fazeka PVHs score** | 0.031 | 1.613 | (1.158, 2.246) | **0.005*** |  | 0.067 | 1.447 | (1.022, 2.051) | **0.038*** |
| **Fazeka DWMH score** | 0.023 | 1.533 | (1.080, 2.175) | **0.017*** |  | 0.062 | 1.378 | (0.964, 1.969) | 0.079 |
| **Fazeka Total score** | 0.034 | 1.325 | (1.096, 1.602) | **0.004*** |  | 0.068 | 1.240 | (1.018, 1.510) | **0.032*** |
| **Scheltens PVH score** | 0.030 | 1.319 | (1.083, 1.606) | **0.006*** |  | 0.065 | 1.231 | (1.001, 1.513) | **0.049*** |
| **Scheltens WMH score** | 0.041 | 1.092 | (1.034, 1.153) | **0.001*** |  | 0.071 | 1.069 | (1.010, 1.131) | **0.021*** |
| **Scheltens BG score** | 0.004 | 1.095 | (0.914, 1.312) | 0.326 |  | 0.051 | 1.045 | (0.867, 1.260) | 0.643 |
| **Scheltens ITF score** | 0.010 | 1.122 | (0.972, 1.295) | 0.117 |  | 0.054 | 1.076 | (0.929, 1.247) | 0.326 |
| **Scheltens total score** | 0.040 | 1.058 | (1.022, 1.097) | **0.002*** |  | 0.069 | 1.043 | (1.005, 1.082) | **0.027*** |
| **WMH volume/ICV*100%** | 0.050 | 1.438 | (1.171, 1.765) | **0.001*** |  | 0.077 | 1.348 | (1.086, 1.073) | **0.007*** |
| **Brain volume** | 0.002 | 0.999 | (0.997, 1.002) | 0.517 |  | 0.048 | 1.000 | (0.998, 1.002) | 0.994 |
| **CSF volume** | 0.076 | 1.009 | (1.005, 1.014) | **<0.001*** |  | 0.111 | 1.009 | (1.004, 1.014) | **<0.001*** |
| **Trial intervention (allopurinol)** | 0.001 | 0.882 | (0.550, 1.414) | 0.602 |  | 0.052 | 0.870 | (0.538, 1.405) | 0.568 |

**Table S8:** **Predictors of cognitive impairment at year 2 after stroke.**

Model 1: Logistic regression with no adjustment; Model 2: Logistic regression adjusting for age and education year. The reference group was participants having not cognitive impairment. *: *p<*0.05.

## Table S9: Predictors of cognitive improvement in the second year after stroke.

|  | **Model 1** | | | |  | **Model 2** | | | |
| --- | --- | --- | --- | --- | --- | --- | --- | --- | --- |
| **Variable** | **Pseudo R^2^** | **OR** | **95% CI** | ***p* value** |  | **Pseudo R^2^** | **OR** | **95% CI** | ***p* value** |
| **Age** | 0.006 | 1.018 | (0.988, 1.049) | 0.234 |  |  |  |  |  |
| **Education year** | <0.001 | 0.996 | (0.908, 1.093) | 0.932 |  |  |  |  |  |
| **Female** | 0.014 | 1.624 | (0.963, 2.738) | 0.069 |  | 0.020 | 1.627 | (0.963, 2.749) | 0.069 |
| **Left-handedness** | <0.001 | 0.866 | (0.316, 2.378) | 0.781 |  | 0.007 | 0.850 | (0.309, 2.338) | 0.753 |
| **Smoker** | 0.049 | 2.769 | (1.564, 4.903) | **<0.001*** |  | 0.067 | 3.325 | (1.805, 6.125) | **<0.001*** |
| **Alcohol use** | 0.009 | 0.668 | (0.390, 1.143) | 0.141 |  | 0.014 | 0.682 | (0.397, 1.169) | 0.164 |
| **Myocardial infarction** | 0.003 | 0.658 | (0.245, 1.771) | 0.408 |  | 0.009 | 0.667 | (0.247, 1.797) | 0.423 |
| **Previous stroke** | 0.002 | 1.311 | (0.562, 3.058) | 0.531 |  | 0.008 | 1.369 | (0.583, 3.219) | 0.471 |
| **PAD** | 0.001 | 1.241 | (0.436, 3.528) | 0.686 |  | 0.007 | 1.214 | (0.426, 3.462) | 0.717 |
| **Carotid artery disease** | 0.010 | 0.462 | (0.158, 1.354) | 0.159 |  | 0.016 | 0.458 | (0.156, 1.346) | 0.156 |
| **Hypertension** | 0.020 | 1.742 | (1.042, 2.913) | **0.034*** |  | 0.023 | 1.684 | (0.998, 2.842) | 0.051 |
| **Diabetes** | 0.003 | 0.771 | (0.398, 1.495) | 0.441 |  | 0.009 | 0.756 | (0.388, 1.474) | 0.412 |
| **COPD** | 0.011 | 2.071 | (0.885, 4.849) | 0.093 |  | 0.018 | 2.136 | (0.906, 5.037) | 0.083 |
| **Event type (TIA with positive imaging)** | <0.001 | 0.866 | (0.316, 2.378) | 0.781 |  | 0.007 | 0.843 | (0.306, 2.324) | 0.742 |
| **TOAST_SVO** | <0.001 | 0.906 | (0.511, 1.605) | 0.735 |  | 0.007 | 0.881 | (0.496, 1.566) | 0.667 |
| **NIHSS** | 0.001 | 1.040 | (0.901, 1.201) | 0.595 |  | 0.008 | 1.050 | (0.907, 1.215) | 0.512 |
| **mRS** | <0.001 | 1.044 | (0.810, 1.345) | 0.740 |  | 0.007 | 1.051 | (0.814, 1.356) | 0.702 |
| **SBP (mmHg)** | <0.001 | 1.001 | (0.986, 1.016) | 0.922 |  | 0.006 | 1.000 | (0.985, 1.015) | 0.988 |
| **DBP (mmHg)** | 0.001 | 1.006 | (0.983, 1.030) | 0.611 |  | 0.009 | 1.010 | (0.986, 1.034) | 0.437 |
| **BMI (km/m2)** | 0.003 | 1.021 | (0.972, 1.072) | 0.406 |  | 0.008 | 1.025 | (0.976, 1.078) | 0.323 |
| **Haemoglobin (g/dL)** | 0.001 | 0.963 | (0.809, 1.147) | 0.675 |  | 0.006 | 0.982 | (0.821, 1.175) | 0.843 |
| **Cholesterol (mmol/L)** | 0.018 | 0.748 | (0.558, 1.004) | 0.053 |  | 0.021 | 0.765 | (0.567, 1.032) | 0.080 |
| **Albumin (g/dL)** | 0.031 | 0.914 | (0.855, 0.977) | **0.008*** |  | 0.034 | 0.917 | (0.857, 0.981) | **0.012*** |
| **Creatinine (μmol/L)** | 0.008 | 0.990 | (0.976, 1.005) | 0.188 |  | 0.017 | 0.988 | (0.974, 1.003) | 0.126 |
| **eGFR (mL/min)** | <0.001 | 1.000 | (0.988, 1.013) | 0.971 |  | 0.007 | 1.003 | (0.989, 1.016) | 0.700 |
| **Fazeka PVHs score** | 0.006 | 1.245 | (0.870, 1.783) | 0.231 |  | 0.009 | 1.188 | (0.816, 1.729) | 0.369 |
| **Fazeka DWMH score** | 0.002 | 1.127 | (0.770, 1.650) | 0.537 |  | 0.007 | 1.091 | (0.738, 1.612) | 0.664 |
| **Fazeka Total score** | 0.004 | 1.113 | (0.906, 1.367) | 0.308 |  | 0.008 | 1.085 | (0.876, 1.345) | 0.453 |
| **Scheltens PVH score** | 0.003 | 1.090 | (0.879, 1.351) | 0.431 |  | 0.007 | 1.056 | (0.845, 1.321) | 0.631 |
| **Scheltens WMH score** | 0.008 | 1.042 | (0.984, 1.103) | 0.162 |  | 0.011 | 1.035 | (0.975, 1.100) | 0.261 |
| **Scheltens BG score** | 0.035 | 1.315 | (1.093, 1.583) | **0.004*** |  | 0.038 | 1.304 | (1.081, 1.573) | **0.006*** |
| **Scheltens ITF score** | 0.003 | 1.065 | (0.913, 1.243) | 0.425 |  | 0.008 | 1.054 | (0.900, 1.235) | 0.511 |
| **Scheltens total score** | 0.014 | 1.035 | (0.997, 1.075) | 0.069 |  | 0.016 | 1.032 | (0.992, 1.074) | 0.118 |
| **WMH volume/ICV*100%** | 0.002 | 1.076 | (0.864, 1.341) | 0.512 |  | 0.008 | 1.030 | (0.813, 1.306) | 0.805 |
| **Brain volume** | 0.060 | 0.995 | (0.993, 0.998) | **<0.001*** |  | 0.061 | 0.995 | (0.993, 0.998) | **0.001*** |
| **CSF volume** | 0.004 | 0.998 | (0.993, 1.002) | 0.337 |  | 0.018 | 0.996 | (0.992, 1.001) | 0.142 |
| **Trial intervention (allopurinol)** | 0.003 | 0.819 | (0.494, 1.359) | 0.440 |  | 0.009 | 0.816 | (0.491, 1.355) | 0.432 |

**Table S9: Predictors of cognitive improvement in the second year after stroke.**

Model 1: Logistic regression with no adjustment; Model 2: Logistic regression adjusting for age and education year. The reference group was participants having any other cognitive trajectories. *: *p<*0.05.

## Table S10: Predictors of cognitive improvement then stable cognition after stroke.

|  | **Model 1** | | | |  | **Model 2** | | | |
| --- | --- | --- | --- | --- | --- | --- | --- | --- | --- |
| **Variable** | **Pseudo R^2^** | **OR** | **95% CI** | ***p* value** |  | **Pseudo R^2^** | **OR** | **95% CI** | ***p* value** |
| **Age** | 0.004 | 0.985 | (0.955, 1.017) | 0.365 |  |  |  |  |  |
| **Education year** | <0.001 | 1.015 | (0.923, 1.116) | 0.758 |  |  |  |  |  |
| **Female** | 0.006 | 0.700 | (0.388, 1.261) | 0.234 |  | 0.011 | 0.697 | (0.386, 1.258) | 0.231 |
| **Left-handedness** | <0.001 | 0.985 | (0.358, 2.711) | 0.977 |  | 0.004 | 1.000 | (0.363, 2.756) | 0.999 |
| **Smoker** | 0.025 | 0.400 | (0.175, 0.917) | **0.030*** |  | 0.033 | 0.363 | (0.156, 0.848) | **0.019*** |
| **Alcohol use** | <0.001 | 0.986 | (0.553, 1.757) | 0.961 |  | 0.004 | 0.963 | (0.539, 1.722) | 0.900 |
| **Myocardial infarction** | 0.007 | 1.713 | (0.755, 3.886) | 0.198 |  | 0.011 | 1.708 | (0.752, 3.883) | 0.201 |
| **Previous stroke** | 0.001 | 1.231 | (0.508, 2.986) | 0.645 |  | 0.005 | 1.211 | (0.497, 2.951) | 0.674 |
| **PAD** | 0.016 | 0.207 | (0.027, 1.571) | 0.128 |  | 0.020 | 0.211 | (0.028, 1.609) | 0.133 |
| **Carotid artery disease** | <0.001 | 0.877 | (0.349, 2.208) | 0.781 |  | 0.004 | 0.889 | (0.352, 2.242) | 0.803 |
| **Hypertension** | <0.001 | 0.986 | (0.585, 1.662) | 0.959 |  | 0.004 | 1.036 | (0.608, 1.766) | 0.896 |
| **Diabetes** | 0.008 | 0.613 | (0.297, 1.267) | 0.186 |  | 0.012 | 0.622 | (0.300, 1.292) | 0.203 |
| **COPD** | <0.001 | 0.985 | (0.358, 2.711) | 0.977 |  | 0.004 | 0.977 | (0.353, 2.703) | 0.965 |
| **Event type (TIA with positive imaging)** | 0.006 | 0.520 | (0.152, 1.783) | 0.298 |  | 0.009 | 0.534 | (0.155, 1.836) | 0.319 |
| **TOAST_SVO** | 0.011 | 0.603 | (0.319, 1.141) | 0.120 |  | 0.015 | 0.614 | (0.324, 1.163) | 0.135 |
| **NIHSS** | 0.016 | 0.838 | (0.691, 1.017) | 0.074 |  | 0.021 | 0.834 | (0.688, 1.012) | 0.066 |
| **mRS** | 0.016 | 0.764 | (0.579, 1.009) | 0.058 |  | 0.021 | 0.761 | (0.575, 1.005) | 0.054 |
| **SBP (mmHg)** | 0.005 | 0.992 | (0.976, 1.007) | 0.291 |  | 0.008 | 0.992 | (0.977, 1.008) | 0.333 |
| **DBP (mmHg)** | 0.001 | 1.005 | (0.981, 1.030) | 0.663 |  | 0.004 | 1.003 | (0.978, 1.029) | 0.805 |
| **BMI (km/m2)** | 0.001 | 1.009 | (0.960, 1.062) | 0.717 |  | 0.004 | 1.008 | (0.957, 1.061) | 0.772 |
| **Haemoglobin (g/dL)** | 0.013 | 1.174 | (0.973, 1.415) | 0.093 |  | 0.015 | 1.166 | (0.963, 1.413) | 0.116 |
| **Cholesterol (mmol/L)** | <0.001 | 1.018 | (0.765, 1.353) | 0.905 |  | 0.005 | 1.002 | (0.748, 1.343) | 0.987 |
| **Albumin (g/dL)** | <0.001 | 1.004 | (0.940, 1.072) | 0.914 |  | 0.004 | 1.000 | (0.936, 1.069) | 0.996 |
| **Creatinine (μmol/L)** | <0.001 | 1.001 | (0.987, 1.015) | 0.867 |  | 0.004 | 1.002 | (0.988, 1.017) | 0.742 |
| **eGFR (mL/min)** | <0.001 | 1.001 | (0.988, 1.014) | 0.884 |  | 0.004 | 0.999 | (0.986, 1.013) | 0.919 |
| **Fazeka PVHs score** | 0.027 | 0.577 | (0.360, 0.925) | **0.022*** |  | 0.027 | 0.587 | (0.361, 0.955) | **0.032*** |
| **Fazeka DWMH score** | 0.012 | 0.699 | (0.451, 1.085) | 0.111 |  | 0.014 | 0.717 | (0.459, 1.121) | 0.144 |
| **Fazeka Total score** | 0.023 | 0.751 | (0.580, 0.972) | **0.030*** |  | 0.024 | 0.760 | (0.583, 0.991) | **0.043*** |
| **Scheltens PVH score** | 0.019 | 0.772 | (0.598, 0.997) | **0.047*** |  | 0.020 | 0.783 | (0.602, 1.019) | 0.068 |
| **Scheltens WMH score** | 0.034 | 0.912 | (0.853, 0.976) | **0.007*** |  | 0.034 | 0.913 | (0.852, 0.979) | **0.011*** |
| **Scheltens BG score** | 0.001 | 0.939 | (0.755, 1.168) | 0.570 |  | 0.005 | 0.952 | (0.764, 1.187) | 0.663 |
| **Scheltens ITF score** | <0.001 | 0.999 | (0.844, 1.181) | 0.987 |  | 0.004 | 1.012 | (0.854, 1.200) | 0.890 |
| **Scheltens total score** | 0.024 | 0.951 | (0.910, 0.994) | **0.025*** |  | 0.024 | 0.952 | (0.909, 0.997) | **0.037*** |
| **WMH volume/ICV*100%** | 0.030 | 0.681 | (0.492, 0.943) | **0.021*** |  | 0.030 | 0.674 | (0.477, 0.952) | **0.025*** |
| **Brain volume** | <0.001 | 1.000 | (0.997, 1.002) | 0.823 |  | 0.004 | 0.999 | (0.997, 1.002) | 0.655 |
| **CSF volume** | <0.001 | 1.000 | (0.996, 1.005) | 0.873 |  | 0.003 | 1.001 | (0.996, 1.006) | 0.681 |
| **Trial intervention (allopurinol)** | <0.001 | 0.956 | (0.567, 1.612) | 0.865 |  | 0.004 | 0.959 | (0.568, 1.619) | 0.877 |

**Table S10: Predictors of cognitive improvement then stable cognition after stroke.**

Model 1: Logistic regression with no adjustment; Model 2: Logistic regression adjusting for age and education year. The reference group was participants having any other cognitive trajectories. *: *p<*0.05.

## Table S11: Predictors of continuous cognitive improvement over 2 years after stroke

| **Variable** | **OR/mean difference** | **95% CI** | **p value** |
| --- | --- | --- | --- |
| **Age** | 0.996 | (0.930, 1.068) | 0.917 |
| **Education year** | 1.064 | (0.875, 1.293) | 0.535 |
| **Female** | 1.566 | (0.486, 5.045) | 0.452 |
| **Left-handedness** | 2.700 | (0.560, 13.027) | 0.216 |
| **Smoker** | 0.824 | (0.176, 3.846) | 0.805 |
| **Alcohol use** | 0.381 | (0.120, 1.210) | 0.102 |
| **Myocardial infarction** | 0.930 | (0.116, 7.442) | 0.945 |
| **Previous stroke** | 0.964 | (0.120, 7.722) | 0.972 |
| **PAD** | 1.574 | (0.193, 12.838) | 0.672 |
| **Carotid artery disease** | 0.868 | (0.109, 6.934) | 0.894 |
| **Hypertension** | 0.989 | (0.313, 3.125) | 0.984 |
| **Diabetes** | 0.355 | (0.045, 2.793) | 0.325 |
| **COPD** | 1.175 | (0.146, 9.469) | 0.880 |
| **Event type (TIA with positive imaging) (a)** | 0.964 | (0.944, 0.984) | 1.000 |
| **TOAST_SVO** | 0.232 | (0.030, 1.820) | 0.164 |
| **NIHSS** | 1.260 | (1.006, 1.579) | 0.045* |
| **mRS** | 1.094 | (0.617, 1.939) | 0.759 |
| **SBP (mmHg)** | 0.994 | (0.960, 1.029) | 0.715 |
| **DBP (mmHg)** | 0.998 | (0.946, 1.054) | 0.952 |
| **BMI (km/m2)** | 1.049 | (0.946, 1.162) | 0.364 |
| **Haemoglobin (g/dL)** | 0.822 | (0.561, 1.204) | 0.313 |
| **Cholesterol (mmol/L)** | 1.006 | (0.545, 1.856) | 0.986 |
| **Albumin (g/dL)** | 0.964 | (0.833, 1.117) | 0.630 |
| **Creatinine (μmol/L)** | 1.012 | (0.984, 1.041) | 0.418 |
| **eGFR (mL/min)** | 0.978 | (0.948, 1.009) | 0.160 |
| **Fazeka PVHs score** | 0.981 | (0.413, 2.329) | 0.965 |
| **Fazeka DWMH score** | 1.090 | (0.459, 2.589) | 0.844 |
| **Fazeka Total score** | 1.021 | (0.631, 1.651) | 0.934 |
| **Scheltens PVH score** | 0.993 | (0.600, 1.644) | 0.980 |
| **Scheltens WMH score** | 0.978 | (0.853, 1.121) | 0.746 |
| **Scheltens BG score** | 0.931 | (0.568, 1.527) | 0.778 |
| **Scheltens ITF score** | 1.079 | (0.774, 1.503) | 0.655 |
| **Scheltens total score** | 0.992 | (0.907, 1.086) | 0.868 |
| **WMH volume/ICV*100%** | 0.666 | (0.299, 1.487) | 0.321 |
| **Brain volume** | 0.997 | (0.991, 1.002) | 0.230 |
| **CSF volume** | 0.994 | (0.984, 1.005) | 0.273 |
| **Trial intervention (allopurinol)** | 0.765 | (0.238, 2.458) | 0.653 |

**Table S11: Predictors of continuous cognitive improvement over 2 years after stroke.**

Logistic regression with no adjustment. (a): Fisher’s exact test. The reference group was participants having any other cognitive trajectories. *: *p<*0.05.

## Table S12: Predictors of cognitive decline in the second year after stroke.

|  | **Model 1** | | | |  | **Model 2** | | | |
| --- | --- | --- | --- | --- | --- | --- | --- | --- | --- |
| **Variable** | **Pseudo R^2^** | **OR** | **95% CI** | ***p* value** |  | **Pseudo R^2^** | **OR** | **95% CI** | ***p* value** |
| **Age** | 0.002 | 1.010 | (0.981, 1.040) | 0.483 |  |  |  |  |  |
| **Education year** | 0.010 | 0.927 | (0.843, 1.021) | 0.123 |  |  |  |  |  |
| **Female** | 0.001 | 1.105 | (0.657, 1.859) | 0.708 |  | 0.013 | 1.134 | (0.672, 1.914) | 0.639 |
| **Left-handedness** | 0.001 | 1.230 | (0.498, 3.034) | 0.654 |  | 0.013 | 1.237 | (0.499, 3.066) | 0.646 |
| **Smoker** | 0.001 | 0.874 | (0.464, 1.643) | 0.675 |  | 0.013 | 0.837 | (0.437, 1.605) | 0.593 |
| **Alcohol use** | 0.001 | 1.150 | (0.663, 1.997) | 0.619 |  | 0.014 | 1.175 | (0.675, 2.046) | 0.569 |
| **Myocardial infarction** | <0.001 | 1.105 | (0.477, 2.561) | 0.815 |  | 0.012 | 1.094 | (0.471, 2.543) | 0.834 |
| **Previous stroke** | 0.002 | 1.385 | (0.612, 3.137) | 0.434 |  | 0.014 | 1.356 | (0.594, 3.091) | 0.470 |
| **PAD** | 0.030 | 3.595 | (1.442, 8.962) | **0.006*** |  | 0.040 | 3.481 | (1.392, 8.708) | **0.008*** |
| **Carotid artery disease** | 0.012 | 1.924 | (0.909, 4.076) | 0.087 |  | 0.023 | 1.868 | (0.879, 3.968) | 0.104 |
| **Hypertension** | <0.001 | 0.986 | (0.605, 1.606) | 0.954 |  | 0.013 | 0.927 | (0.563, 1.525) | 0.764 |
| **Diabetes** | 0.001 | 0.835 | (0.445, 1.568) | 0.575 |  | 0.015 | 0.775 | (0.409, 1.470) | 0.435 |
| **COPD** | 0.034 | 0.121 | (0.016, 0.907) | **0.040*** |  | 0.047 | 0.115 | (0.015, 0.865) | **0.036*** |
| **Event type (TIA with positive imaging)** | 0.001 | 0.769 | (0.281, 2.105) | 0.609 |  | 0.014 | 0.724 | (0.264, 1.991) | 0.532 |
| **TOAST_SVO** | 0.019 | 1.789 | (1.063, 3.012) | **0.029*** |  | 0.031 | 1.783 | (1.055, 3.012) | **0.031*** |
| **NIHSS** | <0.001 | 1.020 | (0.885, 1.176) | 0.784 |  | 0.012 | 1.017 | (0.880, 1.174) | 0.821 |
| **mRS** | 0.001 | 1.052 | (0.823, 1.346) | 0.684 |  | 0.013 | 1.045 | (0.816, 1.338) | 0.728 |
| **SBP (mmHg)** | 0.004 | 1.007 | (0.993, 1.021) | 0.349 |  | 0.015 | 1.006 | (0.992, 1.021) | 0.402 |
| **DBP (mmHg)** | <0.001 | 0.999 | (0.977, 1.023) | 0.960 |  | 0.012 | 1.002 | (0.978, 1.025) | 0.899 |
| **BMI (km/m2)** | 0.001 | 1.009 | (0.962, 1.058) | 0.723 |  | 0.013 | 1.007 | (0.959, 1.057) | 0.785 |
| **Haemoglobin (g/dL)** | 0.006 | 0.901 | (0.761, 1.068) | 0.229 |  | 0.019 | 0.896 | (0.753, 1.066) | 0.214 |
| **Cholesterol (mmol/L)** | 0.029 | 1.407 | (1.088, 1.820) | **0.009*** |  | 0.040 | 1.429 | (1.097, 1.862) | **0.008*** |
| **Albumin (g/dL)** | 0.003 | 1.028 | (0.967, 1.093) | 0.376 |  | 0.015 | 1.029 | (0.967, 1.095) | 0.374 |
| **Creatinine (μmol/L)** | 0.001 | 0.997 | (0.984, 1.011) | 0.669 |  | 0.013 | 0.996 | (0.983, 1.010) | 0.594 |
| **eGFR (mL/min)** | 0.006 | 1.007 | (0.995, 1.020) | 0.248 |  | 0.020 | 1.009 | (0.996, 1.022) | 0.175 |
| **Fazeka PVHs score** | 0.024 | 1.529 | (1.087, 2.151) | **0.015*** |  | 0.032 | 1.505 | (1.053, 2.151) | **0.025*** |
| **Fazeka DWMH score** | 0.002 | 1.157 | (0.800, 1.672) | 0.439 |  | 0.013 | 1.099 | (0.756, 1.598) | 0.620 |
| **Fazeka Total score** | 0.013 | 1.200 | (0.986, 1.461) | 0.069 |  | 0.022 | 1.174 | (0.957, 1.439) | 0.123 |
| **Scheltens PVH score** | 0.010 | 1.176 | (0.958, 1.444) | 0.122 |  | 0.019 | 1.152 | (0.930, 1.429) | 0.196 |
| **Scheltens WMH score** | 0.013 | 1.052 | (0.995, 1.112) | 0.072 |  | 0.021 | 1.044 | (0.985, 1.107) | 0.145 |
| **Scheltens BG score** | 0.003 | 1.085 | (0.900, 1.309) | 0.392 |  | 0.014 | 1.065 | (0.880, 1.288) | 0.520 |
| **Scheltens ITF score** | <0.001 | 1.010 | (0.864, 1.180) | 0.900 |  | 0.012 | 0.987 | (0.842, 1.158) | 0.874 |
| **Scheltens total score** | 0.011 | 1.031 | (0.994, 1.070) | 0.098 |  | 0.019 | 1.025 | (0.987, 1.066) | 0.199 |
| **WMH volume/ICV*100%** | 0.035 | 1.353 | (1.102, 1.660) | **0.004*** |  | 0.042 | 1.346 | (1.083, 1.675) | **0.008*** |
| **Brain volume** | 0.002 | 1.001 | (0.998, 1.003) | 0.522 |  | 0.018 | 1.001 | (0.999, 1.004) | 0.350 |
| **CSF volume** | 0.002 | 1.002 | (0.997, 1.006) | 0.464 |  | 0.016 | 1.001 | (0.997, 1.006) | 0.544 |
| **Trial intervention (allopurinol)** | <0.001 | 1.040 | (0.638, 1.696) | 0.874 |  | 0.012 | 1.036 | (0.634, 1.693) | 0.887 |

**Table S12: Predictors of cognitive decline in the second year after stroke.**

Model 1: Logistic regression with no adjustment; Model 2: Logistic regression adjusting for age and education year. The reference group was participants having any other cognitive trajectories. *: *p<*0.05.

## Table S13: Predictors of delayed cognitive decline after stroke.

|  | **Model 1** | | | |  | **Model 2** | | | |
| --- | --- | --- | --- | --- | --- | --- | --- | --- | --- |
| **Variable** | **Pseudo R^2^** | **OR** | **95% CI** | ***p* value** |  | **Pseudo R^2^** | **OR** | **95% CI** | ***p* value** |
| **Age** | <0.001 | 0.998 | (0.969, 1.029) | 0.916 |  |  |  |  |  |
| **Education year** | 0.007 | 0.941 | (0.854, 1.037) | 0.219 |  |  |  |  |  |
| **Female** | 0.003 | 1.276 | (0.753, 2.163) | 0.365 |  | 0.011 | 1.305 | (0.768, 2.218) | 0.325 |
| **Left-handedness** | 0.003 | 0.639 | (0.213, 1.912) | 0.423 |  | 0.010 | 0.647 | (0.216, 1.940) | 0.437 |
| **Smoker** | <0.001 | 0.983 | (0.521, 1.855) | 0.957 |  | 0.007 | 0.913 | (0.474, 1.756) | 0.784 |
| **Alcohol use** | <0.001 | 1.008 | (0.578, 1.760) | 0.977 |  | 0.007 | 1.011 | (0.578, 1.769) | 0.969 |
| **Myocardial infarction** | <0.001 | 1.014 | (0.421, 2.439) | 0.976 |  | 0.007 | 0.995 | (0.413, 2.400) | 0.991 |
| **Previous stroke** | 0.004 | 1.542 | (0.679, 3.499) | 0.301 |  | 0.010 | 1.483 | (0.650, 3.384) | 0.349 |
| **PAD** | 0.024 | 3.213 | (1.281, 8.061) | **0.013*** |  | 0.030 | 3.164 | (1.257, 7.963) | **0.014*** |
| **Carotid artery disease** | 0.005 | 1.581 | (0.721, 3.465) | 0.253 |  | 0.012 | 1.544 | (0.703, 3.391) | 0.279 |
| **Hypertension** | <0.001 | 0.923 | (0.559, 1.525) | 0.756 |  | 0.007 | 0.901 | (0.540, 1.501) | 0.688 |
| **Diabetes** | 0.003 | 0.756 | (0.390, 1.464) | 0.407 |  | 0.011 | 0.715 | (0.366, 1.396) | 0.326 |
| **COPD** | 0.030 | 0.134 | (0.018, 1.001) | 0.050 |  | 0.039 | 0.127 | (0.017, 0.951) | **0.045*** |
| **Event type (TIA with positive imaging)** | 0.003 | 0.639 | (0.213, 1.912) | 0.423 |  | 0.010 | 0.616 | (0.205, 1.848) | 0.387 |
| **TOAST_SVO** | 0.014 | 1.664 | (0.975, 2.842) | 0.062 |  | 0.022 | 1.681 | (0.981, 2.879) | 0.059 |
| **NIHSS** | <0.001 | 1.004 | (0.865, 1.164) | 0.961 |  | 0.007 | 0.996 | (0.858, 1.156) | 0.959 |
| **mRS** | <0.001 | 1.021 | (0.792, 1.314) | 0.875 |  | 0.007 | 1.010 | (0.784, 1.302) | 0.938 |
| **SBP (mmHg)** | 0.003 | 1.007 | (0.992, 1.022) | 0.368 |  | 0.010 | 1.007 | (0.992, 1.021) | 0.379 |
| **DBP (mmHg)** | 0.002 | 1.008 | (0.984, 1.032) | 0.517 |  | 0.009 | 1.008 | (0.984, 1.033) | 0.512 |
| **BMI (km/m2)** | <0.001 | 0.995 | (0.947, 1.046) | 0.852 |  | 0.007 | 0.991 | (0.942, 1.042) | 0.724 |
| **Haemoglobin (g/dL)** | 0.011 | 0.870 | (0.731, 1.035) | 0.116 |  | 0.020 | 0.853 | (0.713, 1.019) | 0.080 |
| **Cholesterol (mmol/L)** | 0.039 | 1.493 | (1.147, 1.942) | **0.003*** |  | 0.042 | 1.490 | (1.137, 1.951) | **0.004*** |
| **Albumin (g/dL)** | 0.002 | 1.025 | (0.962, 1.091) | 0.449 |  | 0.009 | 1.022 | (0.959, 1.089) | 0.502 |
| **Creatinine (μmol/L)** | 0.003 | 0.994 | (0.980, 1.008) | 0.419 |  | 0.009 | 0.994 | (0.980, 1.009) | 0.435 |
| **eGFR (mL/min)** | 0.007 | 1.008 | (0.996, 1.021) | 0.197 |  | 0.014 | 1.009 | (0.995, 1.022) | 0.205 |
| **Fazeka PVHs score** | 0.002 | 1.145 | (0.797, 1.646) | 0.463 |  | 0.009 | 1.149 | (0.787, 1.679) | 0.472 |
| **Fazeka DWMH score** | <0.001 | 0.955 | (0.646, 1.410) | 0.815 |  | 0.007 | 0.930 | (0.626, 1.382) | 0.720 |
| **Fazeka Total score** | <0.001 | 1.031 | (0.836, 1.272) | 0.773 |  | 0.007 | 1.023 | (0.824, 1.270) | 0.838 |
| **Scheltens PVH score** | 0.001 | 1.042 | (0.840, 1.293) | 0.709 |  | 0.007 | 1.038 | (0.829, 1.301) | 0.745 |
| **Scheltens WMH score** | 0.001 | 1.015 | (0.959, 1.075) | 0.604 |  | 0.007 | 1.012 | (0.953, 1.075) | 0.698 |
| **Scheltens BG score** | 0.002 | 1.065 | (0.877, 1.292) | 0.526 |  | 0.008 | 1.057 | (0.868, 1.286) | 0.582 |
| **Scheltens ITF score** | <0.001 | 1.009 | (0.860, 1.185) | 0.909 |  | 0.007 | 0.997 | (0.847, 1.173) | 0.972 |
| **Scheltens total score** | 0.001 | 1.011 | (0.973, 1.050) | 0.577 |  | 0.008 | 1.009 | (0.969, 1.050) | 0.677 |
| **WMH volume/ICV*100%** | 0.009 | 1.170 | (0.948, 1.443) | 0.144 |  | 0.016 | 1.182 | (0.946, 1.476) | 0.142 |
| **Brain volume** | <0.001 | 1.000 | (0.998, 1.003) | 0.763 |  | 0.008 | 1.000 | (0.998, 1.003) | 0.723 |
| **CSF volume** | <0.001 | 1.000 | (0.996, 1.005) | 0.893 |  | 0.008 | 1.001 | (0.996, 1.005) | 0.817 |
| **Trial intervention (allopurinol)** | <0.001 | 1.034 | (0.626, 1.708) | 0.895 |  | 0.007 | 1.033 | (0.625, 1.709) | 0.898 |

**Table S13: Predictors of delayed cognitive decline (cognitive improvement or stable cognition in the first year and cognitive decline in the second year) after stroke.**

Model 1: Logistic regression with no adjustment; Model 2: Logistic regression adjusting for age and education year. The reference group was participants having any other cognitive trajectories. *: *p<*0.05.

## Table S14: Predictors of continuous cognitive decline over 2 years after stroke.

| **Variable** | **OR/mean difference** | **95% CI** | ***p* value** |
| --- | --- | --- | --- |
| **Age** | 9.132 | (2.418, 15.846) | **0.008*** |
| **Education year** | -1.294 | (-3.497, 0.909) | 0.249 |
| **Female (a)** | 0.976 | (0.957,0.995) | 0.183 |
| **Left-handedness (a)** | 14.391 | (2.750,75.324) | **0.006*** |
| **Smoker (a)** | 0.979 | (0.963,0.996) | 0.601 |
| **Alcohol use (a)** | 1.024 | (1.005, 1.043) | 0.190 |
| **Myocardial infarction (a)** | 2.084 | (0.236, 18.406) | 0.430 |
| **Previous stroke (a)** | 0.982 | (0.967, 0.996) | 1.000 |
| **PAD (a)** | 3.526 | (0.392, 31.705) | 0.292 |
| **Carotid artery disease (a)** | 5.031 | (0.887, 28.545) | 0.102 |
| **Hypertension (a)** | 2.000 | (0.362, 11.059) | 0.685 |
| **Diabetes (a)** | 2.029 | (0.364, 11.299) | 0.345 |
| **COPD (a)** | 0.982 | (0.968, 0.996) | 1.000 |
| **Event type (TIA with positive imaging) (a)** | 2.632 | (0.296, 23.405) | 0.364 |
| **TOAST_SVO (a)** | 2.688 | (0.533, 13.544) | 0.352 |
| **NIHSS (a)** | 0.517 | (-0.849, 1.883) | 0.457 |
| **mRS (a)** | 0.342 | (-0.460, 1.143) | 0.402 |
| **SBP (mmHg) (b)** | 1.386 | (-12.418, 15.189) | 0.844 |
| **DBP (mmHg) (b)** | -9.846 | (-18.407, -1.285) | **0.024*** |
| **BMI (km/m2) (b)** | 3.696 | (-0.424, 7.816) | 0.079 |
| **Haemoglobin (g/dL) (b)** | 0.651 | (-0.513, 1.816) | 0.272 |
| **Cholesterol (mmol/L) (b)** | -0.400 | (-1.161, 0.360) | 0.301 |
| **Albumin (g/dL) (b)** | 0.802 | (-2.424, 4.029) | 0.625 |
| **Creatinine (μmol/L) (b)** | 8.949 | (-5.874, 23.772) | 0.236 |
| **eGFR (mL/min) (b)** | -2.733 | (-18.742, 13.277) | 0.737 |
| **Fazeka PVHs score (b)** | 1.602 | (1.534, 1.669) | **<0.001*** |
| **Fazeka DWMH score (b)** | 0.884 | (0.366, 1.402) | **0.001*** |
| **Fazeka Total score (b)** | 2.486 | (1.562, 3.409) | **<0.001*** |
| **Scheltens PVH score (b)** | 1.856 | (0.946, 2.766) | **<0.001*** |
| **Scheltens WMH score (b)** | 7.647 | (4.230, 11.064) | **<0.001*** |
| **Scheltens BG score (b)** | 0.407 | (-0.607, 1.421) | 0.431 |
| **Scheltens ITF score (b)** | 0.031 | (-1.231, 1.293) | 0.961 |
| **Scheltens total score (b)** | 9.941 | (4.744, 15.138) | **<0.001*** |
| **WMH volume/ICV*100%** | 2.363 | (1.499, 3.228) | **<0.001*** |
| **Brain volume (b)** | 49.941 | (-38.203, 138.085) | 0.266 |
| **CSF volume (b)** | 46.524 | (-0.682, 93.729) | 0.053 |
| **Trial intervention (allopurinol)** | 1.082 | (0.216, 5.436) | 1.000 |

**Table S14: Predictors of continuous cognitive decline over 2 years after stroke.**

(a): Fisher’s exact test; (b) T test. The reference group was participants having any other cognitive trajectories. *: *p<*0.05.

## Table S15: Predictors of cognitive impairment in year 1 after stroke (adjusting for allopurinol treatment group).

|  | **Model 3** | | |  | **Model 4** | | |
| --- | --- | --- | --- | --- | --- | --- | --- |
| **Variable** | **OR** | **95% CI** | **p value** |  | **OR** | **95% CI** | **p value** |
| **Age** | 1.046 | (1.016, 1.076) | **0.002*** |  |  |  |  |
| **Education** | 0.897 | (0.815, 0.987) | **0.025*** |  |  |  |  |
| **Female** | 0.822 | (0.491, 1.377) | 0.457 |  | 0.853 | (0.503, 1.444) | 0.553 |
| **Left-handedness** | 2.555 | (1.134, 5.759) | **0.024*** |  | 2.604 | (1.132, 5.987) | **0.024*** |
| **Smoker** | 1.916 | (1.097, 3.345) | **0.022*** |  | 2.275 | (1.250, 4.139) | **0.007*** |
| **Alcohol use** | 0.758 | (0.455, 1.261) | 0.286 |  | 0.792 | (0.472, 1.331) | 0.379 |
| **Myocardial infarction** | 1.097 | (0.487, 2.469) | 0.823 |  | 1.117 | (0.489, 2.554) | 0.793 |
| **Previous stroke** | 1.859 | (0.864, 4.002) | 0.113 |  | 1.986 | (0.903, 4.368) | 0.088 |
| **PAD** | 1.504 | (0.580, 3.903) | 0.401 |  | 1.382 | (0.523, 3.657) | 0.514 |
| **Carotid artery disease** | 0.950 | (0.424, 2.127) | 0.900 |  | 0.894 | (0.393, 2.035) | 0.789 |
| **Hypertension** | 1.419 | (0.884, 2.278) | 0.148 |  | 1.220 | (0.749, 1.988) | 0.425 |
| **Diabetes** | 1.535 | (0.877, 2.688) | 0.134 |  | 1.397 | (0.785, 2.489) | 0.256 |
| **COPD** | 2.589 | (1.149, 5.836) | **0.022*** |  | 2.666 | (1.150, 6.178) | **0.022*** |
| **Event type (TIA with positive imaging)** | 1.290 | (0.540, 3.082) | 0.567 |  | 1.150 | (0.473, 2.792) | 0.758 |
| **TOAST_SVO** | 1.052 | (0.624, 1.776) | 0.848 |  | 1.002 | (0.588, 1.709) | 0.994 |
| **NIHSS** | 1.049 | (0.918, 1.200) | 0.479 |  | 1.064 | (0.925, 1.224) | 0.385 |
| **mRS** | 1.181 | (0.933, 1.494) | 0.167 |  | 1.192 | (0.936, 1.517) | 0.155 |
| **SBP (mmHg)** | 1.009 | (0.995, 1.023) | 0.201 |  | 1.007 | (0.993, 1.021) | 0.327 |
| **DBP (mmHg)** | 0.991 | (0.969, 1.013) | 0.402 |  | 0.998 | (0.976, 1.022) | 0.893 |
| **BMI (km/m2)** | 1.029 | (0.983, 1.077) | 0.218 |  | 1.036 | (0.989, 1.086) | 0.139 |
| **Haemoglobin (g/dL)** | 0.890 | (0.755, 1.048) | 0.163 |  | 0.913 | (0.770, 1.083) | 0.298 |
| **Cholesterol (mmol/L)** | 0.846 | (0.649, 1.102) | 0.216 |  | 0.885 | (0.673, 1.164) | 0.382 |
| **Albumin (g/dL)** | 0.904 | (0.849, 0.963) | **0.002*** |  | 0.905 | (0.849, 0.966) | **0.003*** |
| **Creatinine (μmol/L)** | 1.009 | (0.997, 1.022) | 0.138 |  | 1.006 | (0.993, 1.019) | 0.338 |
| **eGFR (mL/min)** | 0.993 | (0.981, 1.005) | 0.275 |  | 0.998 | (0.985, 1.011) | 0.743 |
| **Fazeka PVHs score** | 1.474 | (1.056, 2.058) | **0.023*** |  | 1.273 | (0.894, 1.813) | 0.180 |
| **Fazeka DWMH score** | 1.525 | (1.075, 2.164) | **0.018*** |  | 1.357 | (0.948, 1.942) | 0.095 |
| **Fazeka Total score** | 1.284 | (1.062, 1.553) | **0.010*** |  | 1.185 | (0.972, 1.444) | 0.094 |
| **Scheltens PVH score** | 1.246 | (1.023, 1.518) | **0.029*** |  | 1.139 | (0.926, 1.402) | 0.217 |
| **Scheltens WMH score** | 1.107 | (1.048, 1.170) | **<0.001*** |  | 1.082 | (1.022, 1.146) | **0.007*** |
| **Scheltens BG score** | 1.141 | (0.954, 1.365) | 0.147 |  | 1.086 | (0.903, 1.307) | 0.382 |
| **Scheltens ITF score** | 1.181 | (1.022, 1.363) | **0.024*** |  | 1.133 | (0.978, 1.313) | 0.096 |
| **Scheltens total score** | 1.067 | (1.030, 1.106) | **<0.001*** |  | 1.050 | (1.012, 1.090) | **0.010*** |
| **WMH volume/ICV** | 1.418 | (1.155, 1.741) | **0.001*** |  | 1.285 | (1.037, 1.592) | **0.022*** |
| **Brain volume** | 0.996 | (0.993, 0.998) | **0.001*** |  | 0.996 | (0.994, 0.999) | **0.004*** |
| **CSF volume** | 1.007 | (1.002, 1.011) | **0.002*** |  | 1.005 | (1.001, 1.010) | **0.016*** |

**Table S15: Predictors of cognitive impairment in year 1 after stroke (adjusting for allopurinol treatment group).**

Model 3: Logistic regression with adjustment for allopurinol treatment group; Model 4: Logistic regression adjusting for age, education and allopurinol treatment group. The reference group was participants having not cognitive impairment. *: *p<*0.05.

## Table S16: Predictors of cognitive impairment in year 2 after stroke (adjusting for allopurinol treatment group).

|  | **Model 3** | | |  | **Model 4** | | |
| --- | --- | --- | --- | --- | --- | --- | --- |
| **Variable** | **OR** | **95% CI** | **p value** |  | **OR** | **95% CI** | **p value** |
| **Age** | 1.037 | (1.008, 1.067) | **0.012*** |  |  |  |  |
| **Education** | 0.886 | (0.803, 0.977) | **0.015*** |  |  |  |  |
| **Female** | 0.673 | (0.397, 1.143) | 0.143 |  | 0.694 | (0.405, 1.189) | 0.184 |
| **Left-handedness** | 2.204 | (0.973, 4.990) | 0.058 |  | 2.242 | (0.973, 5.167) | 0.058 |
| **Smoker** | 1.278 | (0.718, 2.274) | 0.404 |  | 1.366 | (0.746, 2.502) | 0.313 |
| **Alcohol use** | 1.048 | (0.620, 1.771) | 0.861 |  | 1.104 | (0.647, 1.881) | 0.717 |
| **Myocardial infarction** | 1.544 | (0.714, 3.339) | 0.269 |  | 1.576 | (0.719, 3.451) | 0.256 |
| **Previous stroke** | 2.958 | (1.399, 6.255) | **0.005*** |  | 3.135 | (1.452, 6.772) | **0.004*** |
| **PAD** | 4.685 | (1.850, 11.866) | **0.001*** |  | 4.495 | (1.752, 11.534) | **0.002*** |
| **Carotid artery disease** | 1.845 | (0.880, 3.869) | 0.105 |  | 1.775 | (0.836, 3.771) | 0.135 |
| **Hypertension** | 1.472 | (0.916, 2.367) | 0.110 |  | 1.296 | (0.796, 2.112) | 0.297 |
| **Diabetes** | 1.559 | (0.891, 2.728) | 0.120 |  | 1.413 | (0.795, 2.509) | 0.238 |
| **COPD** | 1.279 | (0.536, 3.048) | 0.579 |  | 1.243 | (0.513, 3.015) | 0.630 |
| **Event type (TIA with positive imaging)** | 1.292 | (0.542, 3.082) | 0.563 |  | 1.161 | (0.481, 2.802) | 0.739 |
| **TOAST_SVO** | 1.527 | (0.918, 2.541) | 0.103 |  | 1.487 | (0.886, 2.497) | 0.133 |
| **NIHSS** | 1.015 | (0.885, 1.165) | 0.829 |  | 1.020 | (0.884, 1.177) | 0.786 |
| **mRS** | 1.122 | (0.886, 1.420) | 0.340 |  | 1.123 | (0.882, 1.429) | 0.347 |
| **SBP (mmHg)** | 0.998 | (0.985, 1.012) | 0.818 |  | 0.996 | (0.982, 1.010) | 0.612 |
| **DBP (mmHg)** | 0.967 | (0.944, 0.989) | **0.004*** |  | 0.972 | (0.949, 0.995) | **0.018*** |
| **BMI (km/m2)** | 1.038 | (0.991, 1.086) | 0.113 |  | 1.042 | (0.994, 1.092) | 0.087 |
| **Haemoglobin (g/dL)** | 0.951 | (0.808, 1.120) | 0.550 |  | 0.970 | (0.819, 1.150) | 0.729 |
| **Cholesterol (mmol/L)** | 1.002 | (0.776, 1.295) | 0.987 |  | 1.040 | (0.798, 1.355) | 0.772 |
| **Albumin (g/dL)** | 0.984 | (0.927, 1.044) | 0.584 |  | 0.987 | (0.929, 1.050) | 0.684 |
| **Creatinine (μmol/L)** | 1.009 | (0.997, 1.022) | 0.145 |  | 1.007 | (0.994, 1.020) | 0.292 |
| **eGFR (mL/min)** | 0.997 | (0.985, 1.009) | 0.662 |  | 1.001 | (0.989, 1.014) | 0.850 |
| **Fazeka PVHs score** | 1.620 | (1.162, 2.257) | **0.004*** |  | 1.453 | (1.025, 2.061) | **0.036*** |
| **Fazeka DWMH score** | 1.536 | (1.082, 2.180) | **0.016*** |  | 1.381 | (0.966, 1.973) | 0.076 |
| **Fazeka Total score** | 1.328 | (1.098, 1.605) | **0.003*** |  | 1.242 | (1.020, 1.513) | **0.031*** |
| **Scheltens PVH score** | 1.319 | (1.083, 1.607) | **0.006*** |  | 1.232 | (1.002, 1.514) | **0.048*** |
| **Scheltens WMH score** | 1.093 | (1.036, 1.155) | **0.001*** |  | 1.071 | (1.012, 1.133) | **0.018*** |
| **Scheltens BG score** | 1.091 | (0.910, 1.309) | 0.345 |  | 1.041 | (0.863, 1.255) | 0.677 |
| **Scheltens ITF score** | 1.119 | (0.970, 1.292) | 0.124 |  | 1.074 | (0.927, 1.244) | 0.344 |
| **Scheltens total score** | 1.058 | (1.022, 1.097) | **0.002*** |  | 1.043 | (1.005, 1.083) | **0.026*** |
| **WMH volume/ICV** | 1.440 | (1.172, 1.769) | **0.001*** |  | 1.351 | (1.088, 1.677) | **0.006*** |
| **Brain volume** | 0.999 | (0.997, 1.002) | 0.518 |  | 1.000 | (0.998, 1.002) | 0.995 |
| **CSF volume** | 1.009 | (1.005, 1.014) | **<0.001*** |  | 1.009 | (1.004, 1.014) | **<0.001*** |

**Table S16: Predictors of cognitive impairment in year 2 after stroke (adjusting for allopurinol treatment group).**

Model 3: Logistic regression with adjustment for allopurinol treatment group; Model 4: Logistic regression adjusting for age, education and allopurinol treatment group. The reference group was participants having not cognitive impairment. *: *p<*0.05.

## Table S17: Predictors of cognitive improvement in the second year after stroke (adjusting for allopurinol treatment group).

|  | **Model 3** | | |  | **Model 4** | | |
| --- | --- | --- | --- | --- | --- | --- | --- |
| **Variable** | **OR** | **95% CI** | **p value** |  | **OR** | **95% CI** | **p value** |
| **Age** | 1.019 | (0.988, 1.050) | 0.231 |  |  |  |  |
| **Education** | 0.995 | (0.908, 1.092) | 0.923 |  |  |  |  |
| **Female** | 1.640 | (0.972, 2.769) | 0.064 |  | 1.648 | (0.974, 2.789) | 0.063 |
| **Left-handedness** | 0.855 | (0.311, 2.351) | 0.762 |  | 0.838 | (0.304, 2.309) | 0.733 |
| **Smoker** | 2.791 | (1.574, 4.948) | **<0.001*** |  | 3.345 | (1.814, 6.167) | **<0.001*** |
| **Alcohol use** | 0.669 | (0.391, 1.146) | 0.143 |  | 0.682 | (0.398, 1.170) | 0.165 |
| **Myocardial infarction** | 0.656 | (0.244, 1.766) | 0.404 |  | 0.664 | (0.246, 1.790) | 0.418 |
| **Previous stroke** | 1.303 | (0.558, 3.043) | 0.541 |  | 1.361 | (0.579, 3.201) | 0.480 |
| **PAD** | 1.221 | (0.429, 3.477) | 0.709 |  | 1.190 | (0.416, 3.405) | 0.745 |
| **Carotid artery disease** | 0.443 | (0.150, 1.304) | 0.139 |  | 0.438 | (0.148, 1.292) | 0.135 |
| **Hypertension** | 1.733 | (1.036, 2.899) | **0.036*** |  | 1.672 | (0.990, 2.824) | 0.055 |
| **Diabetes** | 0.772 | (0.398, 1.497) | 0.444 |  | 0.756 | (0.388, 1.475) | 0.412 |
| **COPD** | 2.065 | (0.881, 4.840) | 0.095 |  | 2.125 | (0.900, 5.020) | 0.086 |
| **Event type (TIA with positive imaging)** | 0.877 | (0.319, 2.410) | 0.799 |  | 0.852 | (0.309, 2.351) | 0.757 |
| **TOAST_SVO** | 0.899 | (0.507, 1.594) | 0.717 |  | 0.875 | (0.492, 1.556) | 0.650 |
| **NIHSS** | 1.036 | (0.897, 1.197) | 0.627 |  | 1.046 | (0.904, 1.211) | 0.545 |
| **mRS** | 1.042 | (0.809, 1.343) | 0.748 |  | 1.049 | (0.813, 1.354) | 0.711 |
| **SBP (mmHg)** | 1.001 | (0.986, 1.016) | 0.932 |  | 1.000 | (0.985, 1.015) | 0.980 |
| **DBP (mmHg)** | 1.006 | (0.982, 1.030) | 0.643 |  | 1.009 | (0.985, 1.034) | 0.461 |
| **BMI (km/m2)** | 1.019 | (0.971, 1.070) | 0.440 |  | 1.024 | (0.974, 1.076) | 0.355 |
| **Haemoglobin (g/dL)** | 0.967 | (0.811, 1.152) | 0.704 |  | 0.986 | (0.823, 1.180) | 0.875 |
| **Cholesterol (mmol/L)** | 0.745 | (0.556, 1.000) | 0.050* |  | 0.762 | (0.565, 1.028) | 0.076 |
| **Albumin (g/dL)** | 0.915 | (0.856, 0.978) | **0.009*** |  | 0.918 | (0.858, 0.982) | **0.013*** |
| **Creatinine (μmol/L)** | 0.990 | (0.976, 1.005) | 0.186 |  | 0.988 | (0.974, 1.003) | 0.123 |
| **eGFR (mL/min)** | 1.000 | (0.987, 1.013) | 0.982 |  | 1.003 | (0.989, 1.016) | 0.711 |
| **Fazeka PVHs score** | 1.252 | (0.874, 1.793) | 0.221 |  | 1.194 | (0.819, 1.738) | 0.357 |
| **Fazeka DWMH score** | 1.131 | (0.773, 1.655) | 0.526 |  | 1.093 | (0.740, 1.616) | 0.654 |
| **Fazeka Total score** | 1.116 | (0.908, 1.370) | 0.297 |  | 1.088 | (0.878, 1.347) | 0.442 |
| **Scheltens PVH score** | 1.091 | (0.881, 1.352) | 0.425 |  | 1.057 | (0.846, 1.322) | 0.624 |
| **Scheltens WMH score** | 1.044 | (0.985, 1.105) | 0.146 |  | 1.037 | (0.976, 1.102) | 0.238 |
| **Scheltens BG score** | 1.309 | (1.087, 1.576) | **0.005*** |  | 1.298 | (1.075, 1.567) | **0.007*** |
| **Scheltens ITF score** | 1.061 | (0.909, 1.239) | 0.451 |  | 1.050 | (0.897, 1.230) | 0.544 |
| **Scheltens total score** | 1.036 | (0.998, 1.075) | 0.067 |  | 1.032 | (0.992, 1.074) | 0.117 |
| **WMH volume/ICV** | 1.078 | (0.865, 1.343) | 0.505 |  | 1.032 | (0.814, 1.307) | 0.796 |
| **Brain volume** | 0.995 | (0.993, 0.998) | **<0.001*** |  | 0.995 | (0.993, 0.998) | **0.001*** |
| **CSF volume** | 0.998 | (0.993, 1.002) | 0.318 |  | 0.996 | (0.992, 1.001) | 0.134 |

**Table S17: Predictors of cognitive improvement in the second year after stroke (adjusting for allopurinol treatment group).**

Model 3: Logistic regression with adjustment for allopurinol treatment group; Model 4: Logistic regression adjusting for age, education and allopurinol treatment group. The reference group was participants having not cognitive impairment. *: *p<*0.05.

## Table S18: Predictors of cognitive improvement then stable cognition after stroke (adjusting for allopurinol treatment group).

|  | **Model 3** | | |  | **Model 4** | | |
| --- | --- | --- | --- | --- | --- | --- | --- |
| **Variable** | **OR** | **95% CI** | **p value** |  | **OR** | **95% CI** | **p value** |
| **Age** | 0.985 | (0.955, 1.017) | 0.366 |  |  |  |  |
| **Education** | 1.015 | (0.923, 1.116) | 0.761 |  |  |  |  |
| **Female** | 0.700 | (0.388, 1.263) | 0.237 |  | 0.698 | (0.387, 1.259) | 0.232 |
| **Left-handedness** | 0.983 | (0.357, 2.705) | 0.973 |  | 0.997 | (0.361, 2.751) | 0.996 |
| **Smoker** | 0.401 | (0.175, 0.918) | **0.031*** |  | 0.364 | (0.156, 0.849) | **0.019*** |
| **Alcohol use** | 0.986 | (0.553, 1.758) | 0.962 |  | 0.964 | (0.539, 1.724) | 0.902 |
| **Myocardial infarction** | 1.712 | (0.754, 3.884) | 0.198 |  | 1.708 | (0.751, 3.881) | 0.201 |
| **Previous stroke** | 1.230 | (0.507, 2.982) | 0.647 |  | 1.209 | (0.496, 2.948) | 0.676 |
| **PAD** | 0.206 | (0.027, 1.563) | 0.126 |  | 0.211 | (0.028, 1.603) | 0.132 |
| **Carotid artery disease** | 0.869 | (0.344, 2.198) | 0.767 |  | 0.881 | (0.348, 2.233) | 0.790 |
| **Hypertension** | 0.985 | (0.584, 1.660) | 0.954 |  | 1.035 | (0.607, 1.764) | 0.900 |
| **Diabetes** | 0.613 | (0.297, 1.267) | 0.187 |  | 0.622 | (0.300, 1.292) | 0.203 |
| **COPD** | 0.984 | (0.358, 2.709) | 0.976 |  | 0.977 | (0.353, 2.702) | 0.964 |
| **Event type (TIA with positive imaging)** | 0.521 | (0.152, 1.788) | 0.300 |  | 0.535 | (0.155, 1.841) | 0.321 |
| **TOAST_SVO** | 0.602 | (0.318, 1.139) | 0.119 |  | 0.612 | (0.323, 1.161) | 0.133 |
| **NIHSS** | 0.838 | (0.690, 1.016) | 0.073 |  | 0.834 | (0.688, 1.012) | 0.065 |
| **mRS** | 0.764 | (0.579, 1.009) | 0.058 |  | 0.761 | (0.576, 1.005) | 0.054 |
| **SBP (mmHg)** | 0.992 | (0.976, 1.007) | 0.290 |  | 0.992 | (0.977, 1.008) | 0.331 |
| **DBP (mmHg)** | 1.005 | (0.981, 1.030) | 0.670 |  | 1.003 | (0.978, 1.029) | 0.812 |
| **BMI (km/m2)** | 1.009 | (0.959, 1.062) | 0.725 |  | 1.007 | (0.957, 1.061) | 0.780 |
| **Haemoglobin (g/dL)** | 1.176 | (0.974, 1.418) | 0.091 |  | 1.168 | (0.964, 1.416) | 0.113 |
| **Cholesterol (mmol/L)** | 1.016 | (0.764, 1.351) | 0.913 |  | 1.001 | (0.747, 1.342) | 0.994 |
| **Albumin (g/dL)** | 1.004 | (0.940, 1.072) | 0.908 |  | 1.000 | (0.936, 1.069) | 0.990 |
| **Creatinine (μmol/L)** | 1.001 | (0.987, 1.016) | 0.867 |  | 1.002 | (0.988, 1.017) | 0.742 |
| **eGFR (mL/min)** | 1.001 | (0.988, 1.014) | 0.886 |  | 0.999 | (0.986, 1.013) | 0.917 |
| **Fazeka PVHs score** | 0.577 | (0.360, 0.925) | **0.022*** |  | 0.588 | (0.361, 0.956) | **0.032*** |
| **Fazeka DWMH score** | 0.700 | (0.451, 1.086) | 0.112 |  | 0.718 | (0.459, 1.121) | 0.145 |
| **Fazeka Total score** | 0.751 | (0.580, 0.973) | **0.030*** |  | 0.760 | (0.583, 0.991) | **0.043*** |
| **Scheltens PVH score** | 0.773 | (0.599, 0.997) | **0.048*** |  | 0.783 | (0.602, 1.019) | 0.069 |
| **Scheltens WMH score** | 0.912 | (0.853, 0.976) | **0.007*** |  | 0.913 | (0.851, 0.979) | **0.011*** |
| **Scheltens BG score** | 0.937 | (0.754, 1.166) | 0.562 |  | 0.951 | (0.763, 1.185) | 0.655 |
| **Scheltens ITF score** | 0.998 | (0.843, 1.181) | 0.979 |  | 1.011 | (0.853, 1.199) | 0.897 |
| **Scheltens total score** | 0.951 | (0.910, 0.994) | **0.025*** |  | 0.952 | (0.909, 0.997) | **0.038*** |
| **WMH volume/ICV** | 0.682 | (0.492, 0.944) | **0.021*** |  | 0.675 | (0.478, 0.954) | **0.026*** |
| **Brain volume** | 1.000 | (0.997, 1.002) | 0.826 |  | 0.999 | (0.997, 1.002) | 0.659 |
| **CSF volume** | 1.000 | (0.996, 1.005) | 0.888 |  | 1.001 | (0.996, 1.006) | 0.696 |

**Table S18: Predictors of cognitive improvement then stable cognition after stroke (adjusting for allopurinol treatment group).**

Model 3: Logistic regression with adjustment for allopurinol treatment group; Model 4: Logistic regression adjusting for age, education and allopurinol treatment group. The reference group was participants having not cognitive impairment. *: *p<*0.05.

## Table S19: Predictors of cognitive decline in the second year after stroke (adjusting for allopurinol treatment group).

|  | **Model 3** | | |  | **Model 4** | | |
| --- | --- | --- | --- | --- | --- | --- | --- |
| **Variable** | **OR** | **95% CI** | **p value** |  | **OR** | **95% CI** | **p value** |
| **Age** | 1.010 | (0.981, 1.040) | 0.484 |  |  |  |  |
| **Education** | 0.927 | (0.843, 1.021) | 0.123 |  |  |  |  |
| **Female** | 1.103 | (0.655, 1.857) | 0.712 |  | 1.132 | (0.670, 1.912) | 0.643 |
| **Left-handedness** | 1.233 | (0.499, 3.044) | 0.650 |  | 1.240 | (0.500, 3.075) | 0.642 |
| **Smoker** | 0.873 | (0.464, 1.641) | 0.673 |  | 0.837 | (0.437, 1.604) | 0.591 |
| **Alcohol use** | 1.150 | (0.662, 1.996) | 0.620 |  | 1.175 | (0.674, 2.046) | 0.570 |
| **Myocardial infarction** | 1.106 | (0.477, 2.562) | 0.814 |  | 1.095 | (0.471, 2.545) | 0.832 |
| **Previous stroke** | 1.387 | (0.613, 3.142) | 0.432 |  | 1.357 | (0.595, 3.095) | 0.468 |
| **PAD** | 3.617 | (1.449, 9.031) | **0.006*** |  | 3.504 | (1.399, 8.772) | **0.007*** |
| **Carotid artery disease** | 1.953 | (0.917, 4.160) | 0.083 |  | 1.896 | (0.887, 4.050) | 0.099 |
| **Hypertension** | 0.987 | (0.605, 1.609) | 0.958 |  | 0.928 | (0.563, 1.527) | 0.768 |
| **Diabetes** | 0.835 | (0.445, 1.568) | 0.575 |  | 0.775 | (0.409, 1.469) | 0.435 |
| **COPD** | 0.121 | (0.016, 0.907) | **0.040*** |  | 0.115 | (0.015, 0.865) | **0.036*** |
| **Event type (TIA with positive imaging)** | 0.766 | (0.280, 2.100) | 0.605 |  | 0.723 | (0.263, 1.988) | 0.530 |
| **TOAST_SVO** | 1.793 | (1.065, 3.020) | **0.028*** |  | 1.785 | (1.056, 3.017) | **0.030*** |
| **NIHSS** | 1.021 | (0.885, 1.177) | 0.777 |  | 1.017 | (0.881, 1.176) | 0.814 |
| **mRS** | 1.053 | (0.823, 1.346) | 0.682 |  | 1.045 | (0.816, 1.339) | 0.726 |
| **SBP (mmHg)** | 1.007 | (0.993, 1.021) | 0.348 |  | 1.006 | (0.992, 1.021) | 0.401 |
| **DBP (mmHg)** | 1.000 | (0.977, 1.023) | 0.967 |  | 1.002 | (0.978, 1.025) | 0.892 |
| **BMI (km/m2)** | 1.009 | (0.962, 1.059) | 0.708 |  | 1.007 | (0.959, 1.058) | 0.773 |
| **Haemoglobin (g/dL)** | 0.900 | (0.760, 1.067) | 0.225 |  | 0.895 | (0.752, 1.065) | 0.211 |
| **Cholesterol (mmol/L)** | 1.411 | (1.091, 1.826) | **0.009*** |  | 1.433 | (1.100, 1.867) | **0.008*** |
| **Albumin (g/dL)** | 1.028 | (0.967, 1.093) | 0.379 |  | 1.028 | (0.966, 1.095) | 0.376 |
| **Creatinine (μmol/L)** | 0.997 | (0.984, 1.011) | 0.669 |  | 0.996 | (0.983, 1.010) | 0.595 |
| **eGFR (mL/min)** | 1.007 | (0.995, 1.020) | 0.247 |  | 1.009 | (0.996, 1.022) | 0.174 |
| **Fazeka PVHs score** | 1.529 | (1.087, 2.151) | **0.015*** |  | 1.504 | (1.053, 2.150) | **0.025*** |
| **Fazeka DWMH score** | 1.156 | (0.800, 1.671) | 0.440 |  | 1.099 | (0.756, 1.598) | 0.622 |
| **Fazeka Total score** | 1.200 | (0.985, 1.461) | 0.070 |  | 1.173 | (0.957, 1.439) | 0.124 |
| **Scheltens PVH score** | 1.176 | (0.958, 1.444) | 0.122 |  | 1.152 | (0.929, 1.429) | 0.196 |
| **Scheltens WMH score** | 1.052 | (0.995, 1.112) | 0.073 |  | 1.044 | (0.985, 1.107) | 0.147 |
| **Scheltens BG score** | 1.087 | (0.900, 1.313) | 0.385 |  | 1.066 | (0.880, 1.291) | 0.511 |
| **Scheltens ITF score** | 1.011 | (0.865, 1.182) | 0.893 |  | 0.988 | (0.842, 1.159) | 0.881 |
| **Scheltens total score** | 1.031 | (0.994, 1.070) | 0.098 |  | 1.025 | (0.987, 1.066) | 0.199 |
| **WMH volume/ICV** | 1.352 | (1.102, 1.660) | **0.004*** |  | 1.346 | (1.082, 1.674) | **0.008*** |
| **Brain volume** | 1.001 | (0.998, 1.003) | 0.526 |  | 1.001 | (0.999, 1.004) | 0.351 |
| **CSF volume** | 1.002 | (0.997, 1.006) | 0.451 |  | 1.001 | (0.997, 1.006) | 0.529 |

**Table S19: Predictors of cognitive decline in the second year after stroke (adjusting for allopurinol treatment group).**

Model 3: Logistic regression with adjustment for allopurinol treatment group; Model 4: Logistic regression adjusting for age, education and allopurinol treatment group. The reference group was participants having not cognitive impairment. *: *p<*0.05.

## Table S20: Predictors of delayed cognitive decline after stroke (adjusting for allopurinol treatment group).

|  | **Model 3** | | |  | **Model 4** | | |
| --- | --- | --- | --- | --- | --- | --- | --- |
| **Variable** | **OR** | **95% CI** | **p value** |  | **OR** | **95% CI** | **p value** |
| **Age** | 0.998 | (0.969, 1.029) | 0.915 |  |  |  |  |
| **Education** | 0.941 | (0.854, 1.037) | 0.219 |  |  |  |  |
| **Female** | 1.275 | (0.752, 2.162) | 0.367 |  | 1.304 | (0.767, 2.216) | 0.327 |
| **Left-handedness** | 0.640 | (0.214, 1.916) | 0.425 |  | 0.648 | (0.216, 1.943) | 0.438 |
| **Smoker** | 0.982 | (0.520, 1.854) | 0.955 |  | 0.912 | (0.474, 1.755) | 0.782 |
| **Alcohol use** | 1.008 | (0.577, 1.759) | 0.979 |  | 1.011 | (0.578, 1.769) | 0.970 |
| **Myocardial infarction** | 1.014 | (0.421, 2.441) | 0.975 |  | 0.996 | (0.413, 2.402) | 0.993 |
| **Previous stroke** | 1.543 | (0.680, 3.504) | 0.299 |  | 1.484 | (0.650, 3.389) | 0.348 |
| **PAD** | 3.231 | (1.286, 8.116) | **0.013*** |  | 3.182 | (1.263, 8.018) | **0.014*** |
| **Carotid artery disease** | 1.599 | (0.726, 3.522) | 0.244 |  | 1.562 | (0.707, 3.448) | 0.270 |
| **Hypertension** | 0.924 | (0.560, 1.527) | 0.759 |  | 0.901 | (0.541, 1.503) | 0.691 |
| **Diabetes** | 0.756 | (0.390, 1.464) | 0.406 |  | 0.715 | (0.366, 1.396) | 0.325 |
| **COPD** | 0.134 | (0.018, 1.002) | 0.050 |  | 0.127 | (0.017, 0.952) | **0.045*** |
| **Event type (TIA with positive imaging)** | 0.637 | (0.213, 1.908) | 0.421 |  | 0.614 | (0.205, 1.845) | 0.385 |
| **TOAST_SVO** | 1.667 | (0.976, 2.848) | 0.061 |  | 1.683 | (0.982, 2.884) | 0.058 |
| **NIHSS** | 1.004 | (0.866, 1.165) | 0.954 |  | 0.997 | (0.859, 1.157) | 0.965 |
| **mRS** | 1.021 | (0.792, 1.315) | 0.874 |  | 1.010 | (0.784, 1.303) | 0.936 |
| **SBP (mmHg)** | 1.007 | (0.992, 1.022) | 0.367 |  | 1.007 | (0.992, 1.021) | 0.378 |
| **DBP (mmHg)** | 1.008 | (0.984, 1.032) | 0.511 |  | 1.008 | (0.984, 1.033) | 0.505 |
| **BMI (km/m2)** | 0.996 | (0.947, 1.047) | 0.865 |  | 0.991 | (0.942, 1.043) | 0.734 |
| **Haemoglobin (g/dL)** | 0.869 | (0.730, 1.034) | 0.114 |  | 0.852 | (0.712, 1.019) | 0.079 |
| **Cholesterol (mmol/L)** | 1.497 | (1.150, 1.949) | **0.003*** |  | 1.494 | (1.140, 1.957) | **0.004*** |
| **Albumin (g/dL)** | 1.024 | (0.962, 1.091) | 0.452 |  | 1.022 | (0.959, 1.089) | 0.505 |
| **Creatinine (μmol/L)** | 0.994 | (0.980, 1.008) | 0.420 |  | 0.994 | (0.980, 1.009) | 0.436 |
| **eGFR (mL/min)** | 1.008 | (0.996, 1.021) | 0.196 |  | 1.009 | (0.995, 1.022) | 0.204 |
| **Fazeka PVHs score** | 1.145 | (0.796, 1.645) | 0.466 |  | 1.149 | (0.786, 1.678) | 0.474 |
| **Fazeka DWMH score** | 0.954 | (0.645, 1.410) | 0.813 |  | 0.930 | (0.626, 1.381) | 0.718 |
| **Fazeka Total score** | 1.031 | (0.836, 1.272) | 0.776 |  | 1.022 | (0.823, 1.270) | 0.841 |
| **Scheltens PVH score** | 1.042 | (0.839, 1.293) | 0.710 |  | 1.038 | (0.828, 1.301) | 0.746 |
| **Scheltens WMH score** | 1.015 | (0.958, 1.075) | 0.610 |  | 1.012 | (0.952, 1.075) | 0.704 |
| **Scheltens BG score** | 1.066 | (0.878, 1.295) | 0.519 |  | 1.058 | (0.869, 1.289) | 0.573 |
| **Scheltens ITF score** | 1.010 | (0.860, 1.186) | 0.903 |  | 0.998 | (0.847, 1.175) | 0.978 |
| **Scheltens total score** | 1.011 | (0.973, 1.050) | 0.579 |  | 1.008 | (0.969, 1.050) | 0.679 |
| **WMH volume/ICV** | 1.169 | (0.948, 1.443) | 0.145 |  | 1.181 | (0.945, 1.476) | 0.143 |
| **Brain volume** | 1.000 | (0.998, 1.003) | 0.767 |  | 1.000 | (0.998, 1.003) | 0.726 |
| **CSF volume** | 1.000 | (0.996, 1.005) | 0.876 |  | 1.001 | (0.996, 1.005) | 0.799 |

**Table S20: Predictors of cognitive decline in the second year after stroke (adjusting for allopurinol treatment group).**

Model 3: Logistic regression with adjustment for allopurinol treatment group; Model 4: Logistic regression adjusting for age, education and allopurinol treatment group. The reference group was participants having not cognitive impairment. *: *p<*0.05.

## Table S21: Difference between original data and imputed data.

|  |  | **N** | **Mean** | **SD** | ***p* value (a)** |
| --- | --- | --- | --- | --- | --- |
| **BMI** | **Original data** | 358 | 28.337 | 5.103 |  |
|  | **imputation 1** | 360 | 28.347 | 5.091 | 0.979 |
|  | **imputation 2** | 360 | 28.321 | 5.093 | 0.967 |
|  | **imputation 3** | 360 | 28.330 | 5.092 | 0.986 |
|  | **imputation 4** | 360 | 28.310 | 5.102 | 0.943 |
|  | **imputation 5** | 360 | 28.342 | 5.090 | 0.989 |
|  | **imputation 6** | 360 | 28.357 | 5.097 | 0.958 |
|  | **imputation 7** | 360 | 28.320 | 5.100 | 0.964 |
|  | **imputation 8** | 360 | 28.317 | 5.100 | 0.957 |
|  | **imputation 9** | 360 | 28.344 | 5.108 | 0.984 |
|  | **imputation 10** | 360 | 28.352 | 5.117 | 0.969 |
|  | **imputation 11** | 360 | 28.347 | 5.091 | 0.978 |
|  | **imputation 12** | 360 | 28.325 | 5.092 | 0.976 |
|  | **imputation 13** | 360 | 28.302 | 5.128 | 0.927 |
|  | **imputation 14** | 360 | 28.283 | 5.141 | 0.887 |
|  | **imputation 15** | 360 | 28.317 | 5.097 | 0.958 |
|  | **imputation 16** | 360 | 28.330 | 5.100 | 0.985 |
|  | **imputation 17** | 360 | 28.385 | 5.143 | 0.899 |
|  | **imputation 18** | 360 | 28.331 | 5.100 | 0.988 |
|  | **imputation 19** | 360 | 28.290 | 5.129 | 0.901 |
|  | **imputation 20** | 360 | 28.321 | 5.094 | 0.966 |
| **Cholesterol** | **Original data** | 346 | 3.860 | 0.939 |  |
|  | **imputation 1** | 360 | 3.855 | 0.938 | 0.943 |
|  | **imputation 2** | 360 | 3.853 | 0.937 | 0.921 |
|  | **imputation 3** | 360 | 3.854 | 0.947 | 0.934 |
|  | **imputation 4** | 360 | 3.842 | 0.950 | 0.797 |
|  | **imputation 5** | 360 | 3.865 | 0.938 | 0.950 |
|  | **imputation 6** | 360 | 3.842 | 0.940 | 0.798 |
|  | **imputation 7** | 360 | 3.865 | 0.959 | 0.948 |
|  | **imputation 8** | 360 | 3.854 | 0.937 | 0.932 |
|  | **imputation 9** | 360 | 3.849 | 0.944 | 0.871 |
|  | **imputation 10** | 360 | 3.854 | 0.954 | 0.933 |
|  | **imputation 11** | 360 | 3.867 | 0.940 | 0.927 |
|  | **imputation 12** | 360 | 3.850 | 0.939 | 0.888 |
|  | **imputation 13** | 360 | 3.868 | 0.961 | 0.915 |
|  | **imputation 14** | 360 | 3.839 | 0.946 | 0.768 |
|  | **imputation 15** | 360 | 3.836 | 0.938 | 0.731 |
|  | **imputation 16** | 360 | 3.845 | 0.931 | 0.826 |
|  | **imputation 17** | 360 | 3.843 | 0.943 | 0.809 |
|  | **imputation 18** | 360 | 3.846 | 0.935 | 0.844 |
|  | **imputation 19** | 360 | 3.865 | 0.947 | 0.948 |
|  | **imputation 20** | 360 | 3.861 | 0.943 | 0.991 |
| **WMH volume/ICV*100%** | **Original data** | 354 | 1.236 | 1.109 |  |
|  | **imputation 1** | 360 | 1.242 | 1.123 | 0.945 |
|  | **imputation 2** | 360 | 1.250 | 1.132 | 0.868 |
|  | **imputation 3** | 360 | 1.243 | 1.128 | 0.937 |
|  | **imputation 4** | 360 | 1.251 | 1.159 | 0.866 |
|  | **imputation 5** | 360 | 1.243 | 1.123 | 0.937 |
|  | **imputation 6** | 360 | 1.241 | 1.112 | 0.960 |
|  | **imputation 7** | 360 | 1.248 | 1.115 | 0.891 |
|  | **imputation 8** | 360 | 1.248 | 1.134 | 0.887 |
|  | **imputation 9** | 360 | 1.244 | 1.122 | 0.925 |
|  | **imputation 10** | 360 | 1.243 | 1.129 | 0.933 |
|  | **imputation 11** | 360 | 1.243 | 1.116 | 0.935 |
|  | **imputation 12** | 360 | 1.238 | 1.111 | 0.982 |
|  | **imputation 13** | 360 | 1.243 | 1.121 | 0.933 |
|  | **imputation 14** | 360 | 1.241 | 1.118 | 0.954 |
|  | **imputation 15** | 360 | 1.244 | 1.123 | 0.924 |
|  | **imputation 16** | 360 | 1.248 | 1.119 | 0.889 |
|  | **imputation 17** | 360 | 1.242 | 1.116 | 0.946 |
|  | **imputation 18** | 360 | 1.243 | 1.129 | 0.938 |
|  | **imputation 19** | 360 | 1.241 | 1.116 | 0.955 |
|  | **imputation 20** | 360 | 1.244 | 1.120 | 0.929 |
| **Brain volume (cm^3^)** | **Original data** | 348 | 1061.650 | 108.861 |  |
|  | **imputation 1** | 360 | 1063.669 | 109.409 | 0.805 |
|  | **imputation 2** | 360 | 1062.160 | 107.992 | 0.950 |
|  | **imputation 3** | 360 | 1062.014 | 108.285 | 0.965 |
|  | **imputation 4** | 360 | 1063.568 | 108.898 | 0.815 |
|  | **imputation 5** | 360 | 1063.469 | 108.691 | 0.824 |
|  | **imputation 6** | 360 | 1065.249 | 110.948 | 0.661 |
|  | **imputation 7** | 360 | 1062.034 | 108.646 | 0.963 |
|  | **imputation 8** | 360 | 1063.471 | 110.505 | 0.824 |
|  | **imputation 9** | 360 | 1061.648 | 108.757 | 1.000 |
|  | **imputation 10** | 360 | 1063.260 | 108.808 | 0.844 |
|  | **imputation 11** | 360 | 1062.284 | 109.261 | 0.938 |
|  | **imputation 12** | 360 | 1062.410 | 108.447 | 0.926 |
|  | **imputation 13** | 360 | 1061.700 | 108.207 | 0.995 |
|  | **imputation 14** | 360 | 1060.989 | 109.580 | 0.936 |
|  | **imputation 15** | 360 | 1060.430 | 108.639 | 0.882 |
|  | **imputation 16** | 360 | 1063.119 | 110.822 | 0.858 |
|  | **imputation 17** | 360 | 1061.997 | 108.563 | 0.966 |
|  | **imputation 18** | 360 | 1063.848 | 110.213 | 0.789 |
|  | **imputation 19** | 360 | 1062.931 | 108.397 | 0.876 |
|  | **imputation 20** | 360 | 1062.062 | 108.159 | 0.960 |
| **CSF volume (cm^3^)** | **Original data** | 348 | 308.155 | 58.511 |  |
|  | **imputation 1** | 360 | 307.545 | 58.216 | 0.890 |
|  | **imputation 2** | 360 | 308.655 | 58.542 | 0.910 |
|  | **imputation 3** | 360 | 308.724 | 59.041 | 0.897 |
|  | **imputation 4** | 360 | 308.646 | 58.157 | 0.911 |
|  | **imputation 5** | 360 | 308.608 | 58.159 | 0.918 |
|  | **imputation 6** | 360 | 308.003 | 58.363 | 0.972 |
|  | **imputation 7** | 360 | 308.297 | 58.483 | 0.974 |
|  | **imputation 8** | 360 | 308.871 | 58.544 | 0.871 |
|  | **imputation 9** | 360 | 307.669 | 58.209 | 0.912 |
|  | **imputation 10** | 360 | 308.815 | 59.742 | 0.881 |
|  | **imputation 11** | 360 | 307.868 | 58.708 | 0.948 |
|  | **imputation 12** | 360 | 309.197 | 58.951 | 0.813 |
|  | **imputation 13** | 360 | 308.246 | 58.308 | 0.984 |
|  | **imputation 14** | 360 | 308.732 | 58.660 | 0.896 |
|  | **imputation 15** | 360 | 307.638 | 58.303 | 0.906 |
|  | **imputation 16** | 360 | 308.035 | 57.871 | 0.978 |
|  | **imputation 17** | 360 | 308.058 | 58.361 | 0.982 |
|  | **imputation 18** | 360 | 308.525 | 59.706 | 0.933 |
|  | **imputation 19** | 360 | 308.997 | 58.534 | 0.848 |
|  | **imputation 20** | 360 | 308.531 | 58.644 | 0.932 |

**Table S21: Difference between original data and imputed data.**

(a)：*p* value of comparison between original data and imputed data.

## Table S22: Predictors of cognitive impairment in year 1 after stroke (multiple imputed data).

|  | **Model 1 (MI)** | | |  | **Model 2 (MI)** | | |
| --- | --- | --- | --- | --- | --- | --- | --- |
| **Variable** | **OR** | **95% CI** | ***p* value** |  | **OR** | **95% CI** | ***p* value** |
| **Age** | 1.046 | (1.016, 1.076) | **0.002*** |  |  |  |  |
| **Education year** | 0.896 | (0.814, 0.987) | **0.026*** |  |  |  |  |
| **Female** | 0.812 | (0.486, 1.359) | 0.428 |  | 0.833 | (0.493, 1.409) | 0.497 |
| **Left-handedness** | 2.592 | (1.153, 5.827) | **0.021*** |  | 2.648 | (1.154, 6.075) | **0.022*** |
| **Smoker** | 1.893 | (1.086, 3.297) | **0.024*** |  | 2.248 | (1.239, 4.077) | **0.008*** |
| **Alcohol use** | 0.756 | (0.455, 1.255) | 0.279 |  | 0.794 | (0.473, 1.333) | 0.384 |
| **Myocardial infarction** | 1.101 | (0.490, 2.473) | 0.815 |  | 1.129 | (0.496, 2.569) | 0.772 |
| **Previous stroke** | 1.872 | (0.872, 4.020) | 0.108 |  | 2.000 | (0.911, 4.390) | 0.084 |
| **PAD** | 1.542 | (0.596, 3.988) | 0.372 |  | 1.436 | (0.548, 3.764) | 0.462 |
| **Carotid artery disease** | 1.005 | (0.451, 2.238) | 0.991 |  | 0.957 | (0.424, 2.159) | 0.915 |
| **Hypertension** | 1.431 | (0.893, 2.295) | 0.137 |  | 1.237 | (0.760, 2.011) | 0.392 |
| **Diabetes** | 1.528 | (0.874, 2.671) | 0.137 |  | 1.393 | (0.784, 2.476) | 0.258 |
| **COPD** | 2.592 | (1.153, 5.827) | **0.021*** |  | 2.677 | (1.162, 6.163) | **0.021*** |
| **Event type (TIA with positive imaging)** | 1.262 | (0.530, 3.006) | 0.599 |  | 1.134 | (0.470, 2.737) | 0.780 |
| **TOAST_SVO** | 1.064 | (0.632, 1.793) | 0.815 |  | 1.009 | (0.592, 1.719) | 0.974 |
| **NIHSS** | 1.055 | (0.923, 1.206) | 0.431 |  | 1.071 | (0.931, 1.231) | 0.336 |
| **mRS** | 1.183 | (0.935, 1.496) | 0.162 |  | 1.194 | (0.938, 1.520) | 0.149 |
| **SBP (mmHg)** | 1.009 | (0.995, 1.023) | 0.195 |  | 1.007 | (0.993, 1.021) | 0.319 |
| **DBP (mmHg)** | 0.991 | (0.970, 1.014) | 0.449 |  | 0.999 | (0.977, 1.022) | 0.958 |
| **BMI (km/m2)** | 1.030 | (0.985, 1.078) | 0.194 |  | 1.037 | (0.990, 1.087) | 0.127 |
| **Haemoglobin (g/dL)** | 0.886 | (0.753, 1.043) | 0.146 |  | 0.910 | (0.768, 1.077) | 0.273 |
| **Cholesterol (mmol/L)** | 0.870 | (0.670, 1.129) | 0.295 |  | 0.911 | (0.695, 1.194) | 0.499 |
| **Albumin (g/dL)** | 0.903 | (0.848, 0.962) | **0.002*** |  | 0.905 | (0.848, 0.965) | **0.002*** |
| **Creatinine (μmol/L)** | 1.009 | (0.997, 1.022) | 0.139 |  | 1.006 | (0.994, 1.019) | 0.332 |
| **eGFR (mL/min)** | 0.993 | (0.982, 1.006) | 0.287 |  | 0.998 | (0.985, 1.011) | 0.767 |
| **Fazeka PVHs score** | 1.459 | (1.047, 2.033) | **0.026*** |  | 1.260 | (0.887, 1.791) | 0.197 |
| **Fazeka DWMH score** | 1.515 | (1.068, 2.148) | **0.020*** |  | 1.348 | (0.942, 1.928) | 0.102 |
| **Fazeka Total score** | 1.278 | (1.058, 1.545) | **0.011*** |  | 1.179 | (0.967, 1.436) | 0.103 |
| **Scheltens PVH score** | 1.243 | (1.021, 1.514) | **0.031*** |  | 1.136 | (0.924, 1.398) | 0.227 |
| **Scheltens WMH score** | 1.103 | (1.045, 1.165) | **<0.001*** |  | 1.078 | (1.018, 1.140) | **0.010*** |
| **Scheltens BG score** | 1.151 | (0.963, 1.376) | 0.121 |  | 1.098 | (0.913, 1.320) | 0.322 |
| **Scheltens ITF score** | 1.186 | (1.028, 1.369) | **0.019*** |  | 1.140 | (0.985, 1.320) | 0.080 |
| **Scheltens total score** | 1.067 | (1.030, 1.106) | **<0.001*** |  | 1.050 | (1.011, 1.090) | **0.011*** |
| **WMH volume/ICV*100%** | 1.397 | (1.143, 1.706) | **0.001*** |  | 1.278 | (1.036, 1.577) | **0.022*** |
| **Brain volume** | 0.996 | (0.993, 0.998) | **0.001*** |  | 0.996 | (0.994, 0.999) | **0.005*** |
| **CSF volume** | 1.007 | (1.002, 1.011) | **0.002*** |  | 1.006 | (1.001, 1.010) | **0.015*** |
| **Trial intervention (allopurinol)** | 0.722 | (0.450, 1.159) | 0.177 |  | 0.703 | (0.434, 1.141) | 0.154 |

**Table S22: Predictors of cognitive impairment in year 2 after stroke (multiple imputed data).**

Model 1 (MI): Logistic regression with no adjustment using multiple imputed data; Model 2 (MI): Logistic regression adjusting for age, education using multiple imputed data. The reference group was participants having not cognitive impairment. *: *p<*0.05.

## Table S23: Predictors of cognitive impairment in year 2 after stroke (multiple imputed data).

|  | **Model 1 (MI)** | | |  | **Model 2 (MI)** | | |
| --- | --- | --- | --- | --- | --- | --- | --- |
| **Variable** | **OR** | **95% CI** | ***p* value** |  | **OR** | **95% CI** | ***p* value** |
| **Age** | 1.037 | (1.008, 1.067) | **0.012*** |  |  |  |  |
| **Education year** | 0.886 | (0.803, 0.977) | **0.015*** |  |  |  |  |
| **Female** | 0.670 | (0.395, 1.138) | 0.138 |  | 0.689 | (0.402, 1.179) | 0.174 |
| **Left-handedness** | 2.218 | (0.980, 5.018) | 0.056 |  | 2.259 | (0.981, 5.204) | 0.056 |
| **Smoker** | 1.274 | (0.716, 2.266) | 0.410 |  | 1.363 | (0.744, 2.494) | 0.316 |
| **Alcohol use** | 1.046 | (0.619, 1.767) | 0.866 |  | 1.104 | (0.648, 1.882) | 0.716 |
| **Myocardial infarction** | 1.546 | (0.715, 3.342) | 0.268 |  | 1.581 | (0.723, 3.458) | 0.252 |
| **Previous stroke** | 2.967 | (1.404, 6.271) | **0.004*** |  | 3.145 | (1.456, 6.792) | **0.004*** |
| **PAD** | 4.720 | (1.865, 11.943) | **0.001*** |  | 4.529 | (1.769, 11.593) | **0.002*** |
| **Carotid artery disease** | 1.872 | (0.897, 3.908) | 0.095 |  | 1.807 | (0.856, 3.817) | 0.121 |
| **Hypertension** | 1.478 | (0.919, 2.375) | 0.107 |  | 1.303 | (0.800, 2.120) | 0.287 |
| **Diabetes** | 1.557 | (0.890, 2.724) | 0.121 |  | 1.411 | (0.795, 2.506) | 0.239 |
| **COPD** | 1.282 | (0.538, 3.054) | 0.575 |  | 1.251 | (0.517, 3.027) | 0.620 |
| **Event type (TIA with positive imaging)** | 1.282 | (0.538, 3.054) | 0.575 |  | 1.155 | (0.479, 2.781) | 0.748 |
| **TOAST_SVO** | 1.533 | (0.922, 2.549) | 0.100 |  | 1.491 | (0.888, 2.503) | 0.131 |
| **NIHSS** | 1.017 | (0.887, 1.167) | 0.806 |  | 1.023 | (0.886, 1.180) | 0.758 |
| **mRS** | 1.123 | (0.887, 1.422) | 0.336 |  | 1.124 | (0.883, 1.430) | 0.343 |
| **SBP (mmHg)** | 0.998 | (0.985, 1.012) | 0.825 |  | 0.996 | (0.982, 1.011) | 0.617 |
| **DBP (mmHg)** | 0.967 | (0.945, 0.990) | **0.005*** |  | 0.972 | (0.950, 0.996) | **0.020*** |
| **BMI (km/m2)** | 1.038 | (0.992, 1.086) | 0.111 |  | 1.042 | (0.994, 1.092) | 0.087 |
| **Haemoglobin (g/dL)** | 0.949 | (0.806, 1.118) | 0.532 |  | 0.969 | (0.818, 1.147) | 0.711 |
| **Cholesterol (mmol/L)** | 1.016 | (0.788, 1.309) | 0.905 |  | 1.057 | (0.812, 1.375) | 0.680 |
| **Albumin (g/dL)** | 0.983 | (0.926, 1.043) | 0.570 |  | 0.987 | (0.928, 1.049) | 0.674 |
| **Creatinine (μmol/L)** | 1.009 | (0.997, 1.022) | 0.145 |  | 1.007 | (0.994, 1.020) | 0.290 |
| **eGFR (mL/min)** | 0.997 | (0.986, 1.009) | 0.669 |  | 1.001 | (0.989, 1.014) | 0.842 |
| **Fazeka PVHs score** | 1.613 | (1.158, 2.246) | **0.005*** |  | 1.447 | (1.022, 2.051) | **0.038*** |
| **Fazeka DWMH score** | 1.533 | (1.080, 2.175) | **0.017*** |  | 1.378 | (0.964, 1.969) | 0.079 |
| **Fazeka Total score** | 1.325 | (1.096, 1.602) | **0.004*** |  | 1.240 | (1.018, 1.510) | **0.032*** |
| **Scheltens PVH score** | 1.319 | (1.083, 1.606) | **0.006*** |  | 1.231 | (1.001, 1.513) | **0.049*** |
| **Scheltens WMH score** | 1.092 | (1.034, 1.153) | **0.001*** |  | 1.069 | (1.010, 1.131) | **0.021*** |
| **Scheltens BG score** | 1.095 | (0.914, 1.312) | 0.326 |  | 1.045 | (0.867, 1.260) | 0.643 |
| **Scheltens ITF score** | 1.122 | (0.972, 1.295) | 0.117 |  | 1.076 | (0.929, 1.247) | 0.326 |
| **Scheltens total score** | 1.058 | (1.022, 1.097) | **0.002*** |  | 1.043 | (1.005, 1.082) | **0.027*** |
| **WMH volume/ICV*100%** | 1.464 | (1.195, 1.792) | **<0.001*** |  | 1.371 | (1.108, 1.697) | **0.004*** |
| **Brain volume** | 0.999 | (0.997, 1.001) | 0.512 |  | 1.000 | (0.998, 1.002) | 0.973 |
| **CSF volume** | 1.010 | (1.005, 1.014) | **<0.001*** |  | 1.009 | (1.005, 1.014) | **<0.001*** |
| **Trial intervention (allopurinol)** | 0.882 | (0.550, 1.414) | 0.602 |  | 0.870 | (0.538, 1.405) | 0.568 |

**Table S23: Predictors of cognitive impairment in year 2 after stroke (multiple imputed data).**

Model 1 (MI): Logistic regression with no adjustment using multiple imputed data; Model 2 (MI): Logistic regression adjusting for age, education using multiple imputed data. The reference group was participants having not cognitive impairment. *: *p<*0.05.

## Table S24: Predictors of cognitive improvement in the second year after stroke (multiple imputed data).

|  | **Model 1 (MI)** | | |  | **Model 2 (MI)** | | |
| --- | --- | --- | --- | --- | --- | --- | --- |
| **Variable** | **OR** | **95% CI** | ***p* value** |  | **OR** | **95% CI** | ***p* value** |
| **Age** | 1.018 | (0.988, 1.049) | 0.234 |  |  |  |  |
| **Education year** | 0.996 | (0.908, 1.093) | 0.932 |  |  |  |  |
| **Female** | 1.624 | (0.963, 2.738) | 0.069 |  | 1.627 | (0.963, 2.749) | 0.069 |
| **Left-handedness** | 0.866 | (0.316, 2.378) | 0.781 |  | 0.850 | (0.309, 2.338) | 0.753 |
| **Smoker** | 2.769 | (1.564, 4.903) | **<0.001*** |  | 3.325 | (1.805, 6.125) | **<0.001*** |
| **Alcohol use** | 0.668 | (0.390, 1.143) | 0.141 |  | 0.682 | (0.397, 1.169) | 0.164 |
| **Myocardial infarction** | 0.658 | (0.245, 1.771) | 0.408 |  | 0.667 | (0.247, 1.797) | 0.423 |
| **Previous stroke** | 1.311 | (0.562, 3.058) | 0.531 |  | 1.369 | (0.583, 3.219) | 0.471 |
| **PAD** | 1.241 | (0.436, 3.528) | 0.686 |  | 1.214 | (0.426, 3.462) | 0.717 |
| **Carotid artery disease** | 0.462 | (0.158, 1.354) | 0.159 |  | 0.458 | (0.156, 1.346) | 0.156 |
| **Hypertension** | 1.742 | (1.042, 2.913) | **0.034*** |  | 1.684 | (0.998, 2.842) | 0.051 |
| **Diabetes** | 0.771 | (0.398, 1.495) | 0.441 |  | 0.756 | (0.388, 1.474) | 0.412 |
| **COPD** | 2.071 | (0.885, 4.849) | 0.093 |  | 2.136 | (0.906, 5.037) | 0.083 |
| **Event type (TIA with positive imaging)** | 0.866 | (0.316, 2.378) | 0.781 |  | 0.843 | (0.306, 2.324) | 0.742 |
| **TOAST_SVO** | 0.906 | (0.511, 1.605) | 0.735 |  | 0.881 | (0.496, 1.566) | 0.667 |
| **NIHSS** | 1.040 | (0.901, 1.201) | 0.595 |  | 1.050 | (0.907, 1.215) | 0.512 |
| **mRS** | 1.044 | (0.810, 1.345) | 0.740 |  | 1.051 | (0.814, 1.356) | 0.702 |
| **SBP (mmHg)** | 1.001 | (0.986, 1.016) | 0.922 |  | 1.000 | (0.985, 1.015) | 0.988 |
| **DBP (mmHg)** | 1.006 | (0.983, 1.030) | 0.611 |  | 1.010 | (0.986, 1.034) | 0.437 |
| **BMI (km/m2)** | 1.020 | (0.971, 1.071) | 0.433 |  | 1.025 | (0.975, 1.077) | 0.333 |
| **Haemoglobin (g/dL)** | 0.963 | (0.809, 1.147) | 0.675 |  | 0.982 | (0.821, 1.175) | 0.843 |
| **Cholesterol (mmol/L)** | 0.774 | (0.578, 1.036) | 0.084 |  | 0.790 | (0.587, 1.064) | 0.121 |
| **Albumin (g/dL)** | 0.914 | (0.855, 0.977) | **0.008*** |  | 0.917 | (0.857, 0.981) | **0.012*** |
| **Creatinine (μmol/L)** | 0.990 | (0.976, 1.005) | 0.188 |  | 0.988 | (0.974, 1.003) | 0.126 |
| **eGFR (mL/min)** | 1.000 | (0.988, 1.013) | 0.971 |  | 1.003 | (0.989, 1.016) | 0.700 |
| **Fazeka PVHs score** | 1.245 | (0.870, 1.783) | 0.231 |  | 1.188 | (0.816, 1.729) | 0.369 |
| **Fazeka DWMH score** | 1.127 | (0.770, 1.650) | 0.537 |  | 1.091 | (0.738, 1.612) | 0.664 |
| **Fazeka Total score** | 1.113 | (0.906, 1.367) | 0.308 |  | 1.085 | (0.876, 1.345) | 0.453 |
| **Scheltens PVH score** | 1.090 | (0.879, 1.351) | 0.431 |  | 1.056 | (0.845, 1.321) | 0.631 |
| **Scheltens WMH score** | 1.042 | (0.984, 1.103) | 0.162 |  | 1.035 | (0.975, 1.100) | 0.261 |
| **Scheltens BG score** | 1.315 | (1.093, 1.583) | **0.004*** |  | 1.304 | (1.081, 1.573) | **0.006*** |
| **Scheltens ITF score** | 1.065 | (0.913, 1.243) | 0.425 |  | 1.054 | (0.900, 1.235) | 0.511 |
| **Scheltens total score** | 1.035 | (0.997, 1.075) | 0.069 |  | 1.032 | (0.992, 1.074) | 0.118 |
| **WMH volume/ICV*100%** | 1.065 | (0.858, 1.323) | 0.568 |  | 1.023 | (0.810, 1.291) | 0.850 |
| **Brain volume** | 0.996 | (0.993, 0.998) | **0.001*** |  | 0.996 | (0.993, 0.998) | **0.002*** |
| **CSF volume** | 0.997 | (0.993, 1.002) | 0.258 |  | 0.996 | (0.992, 1.001) | 0.117 |
| **Trial intervention (allopurinol)** | 0.819 | (0.494, 1.359) | 0.440 |  | 0.816 | (0.491, 1.355) | 0.432 |

**Table S24: Predictors of cognitive improvement in the second year after stroke (multiple imputed data).**

Model 1 (MI): Logistic regression with no adjustment using multiple imputed data; Model 2 (MI): Logistic regression adjusting for age, education using multiple imputed data. The reference group was participants having any other cognitive trajectories. *: *p<*0.05.

## Table S25: Predictors of cognitive improvement then stable cognition after stroke (multiple imputed data).

|  | **Model 1 (MI)** | | |  | **Model 2 (MI)** | | |
| --- | --- | --- | --- | --- | --- | --- | --- |
| **Variable** | **OR** | **95% CI** | ***p* value** |  | **OR** | **95% CI** | ***p* value** |
| **Age** | 0.985 | (0.955, 1.017) | 0.365 |  |  |  |  |
| **Education year** | 1.015 | (0.923, 1.116) | 0.758 |  |  |  |  |
| **Female** | 0.700 | (0.388, 1.261) | 0.234 |  | 0.697 | (0.386, 1.258) | 0.231 |
| **Left-handedness** | 0.985 | (0.358, 2.711) | 0.977 |  | 1.000 | (0.363, 2.756) | 0.999 |
| **Smoker** | 0.400 | (0.175, 0.917) | **0.030*** |  | 0.363 | (0.156, 0.848) | **0.019*** |
| **Alcohol use** | 0.986 | (0.553, 1.757) | 0.961 |  | 0.963 | (0.539, 1.722) | 0.900 |
| **Myocardial infarction** | 1.713 | (0.755, 3.886) | 0.198 |  | 1.708 | (0.752, 3.883) | 0.201 |
| **Previous stroke** | 1.231 | (0.508, 2.986) | 0.645 |  | 1.211 | (0.497, 2.951) | 0.674 |
| **PAD** | 0.207 | (0.027, 1.571) | 0.128 |  | 0.211 | (0.028, 1.609) | 0.133 |
| **Carotid artery disease** | 0.877 | (0.349, 2.208) | 0.781 |  | 0.889 | (0.352, 2.242) | 0.803 |
| **Hypertension** | 0.986 | (0.585, 1.662) | 0.959 |  | 1.036 | (0.608, 1.766) | 0.896 |
| **Diabetes** | 0.613 | (0.297, 1.267) | 0.186 |  | 0.622 | (0.300, 1.292) | 0.203 |
| **COPD** | 0.985 | (0.358, 2.711) | 0.977 |  | 0.977 | (0.353, 2.703) | 0.965 |
| **Event type (TIA with positive imaging)** | 0.520 | (0.152, 1.783) | 0.298 |  | 0.534 | (0.155, 1.836) | 0.319 |
| **TOAST_SVO** | 0.603 | (0.319, 1.141) | 0.120 |  | 0.614 | (0.324, 1.163) | 0.135 |
| **NIHSS** | 0.838 | (0.691, 1.017) | 0.074 |  | 0.834 | (0.688, 1.012) | 0.066 |
| **mRS** | 0.764 | (0.579, 1.009) | 0.058 |  | 0.761 | (0.575, 1.005) | 0.054 |
| **SBP (mmHg)** | 0.992 | (0.976, 1.007) | 0.291 |  | 0.992 | (0.977, 1.008) | 0.333 |
| **DBP (mmHg)** | 1.005 | (0.981, 1.030) | 0.663 |  | 1.003 | (0.978, 1.029) | 0.805 |
| **BMI (km/m2)** | 1.010 | (0.960, 1.062) | 0.706 |  | 1.008 | (0.957, 1.061) | 0.766 |
| **Haemoglobin (g/dL)** | 1.174 | (0.973, 1.415) | 0.093 |  | 1.166 | (0.963, 1.413) | 0.116 |
| **Cholesterol (mmol/L)** | 1.012 | (0.760, 1.347) | 0.936 |  | 0.990 | (0.738, 1.329) | 0.947 |
| **Albumin (g/dL)** | 1.004 | (0.940, 1.072) | 0.914 |  | 1.000 | (0.936, 1.069) | 0.996 |
| **Creatinine (μmol/L)** | 1.001 | (0.987, 1.015) | 0.867 |  | 1.002 | (0.988, 1.017) | 0.742 |
| **eGFR (mL/min)** | 1.001 | (0.988, 1.014) | 0.884 |  | 0.999 | (0.986, 1.013) | 0.919 |
| **Fazeka PVHs score** | 0.577 | (0.360, 0.925) | **0.022*** |  | 0.587 | (0.361, 0.955) | **0.032*** |
| **Fazeka DWMH score** | 0.699 | (0.451, 1.085) | 0.111 |  | 0.717 | (0.459, 1.121) | 0.144 |
| **Fazeka Total score** | 0.751 | (0.580, 0.972) | **0.030*** |  | 0.760 | (0.583, 0.991) | **0.043*** |
| **Scheltens PVH score** | 0.772 | (0.598, 0.997) | **0.047*** |  | 0.783 | (0.602, 1.019) | 0.068 |
| **Scheltens WMH score** | 0.912 | (0.853, 0.976) | **0.007*** |  | 0.913 | (0.852, 0.979) | **0.011*** |
| **Scheltens BG score** | 0.939 | (0.755, 1.168) | 0.570 |  | 0.952 | (0.764, 1.187) | 0.663 |
| **Scheltens ITF score** | 0.999 | (0.844, 1.181) | 0.987 |  | 1.012 | (0.854, 1.200) | 0.890 |
| **Scheltens total score** | 0.951 | (0.910, 0.994) | **0.025*** |  | 0.952 | (0.909, 0.997) | **0.037*** |
| **WMH volume/ICV*100%** | 0.675 | (0.490, 0.931) | **0.016*** |  | 0.676 | (0.481, 0.949) | **0.024*** |
| **Brain volume** | 1.000 | (0.997, 1.002) | 0.838 |  | 0.999 | (0.997, 1.002) | 0.640 |
| **CSF volume** | 1.000 | (0.996, 1.005) | 0.919 |  | 1.001 | (0.996, 1.006) | 0.706 |
| **Trial intervention (allopurinol)** | 0.956 | (0.567, 1.612) | 0.865 |  | 0.959 | (0.568, 1.619) | 0.877 |

**Table S25: Predictors of cognitive improvement then stable cognition after stroke (multiple imputed data).**

Model 1 (MI): Logistic regression with no adjustment using multiple imputed data; Model 2 (MI): Logistic regression adjusting for age, education using multiple imputed data. The reference group was participants having any other cognitive trajectories. *: *p<*0.05.

## Table S26: Predictors of continuous cognitive improvement over 2 years after stroke (multiple imputed data).

|  | **Model 2 (MI)** | | |
| --- | --- | --- | --- |
| **Variable** | **OR** | **95% CI** | ***p* value** |
| **Age** | 0.996 | (0.930, 1.068) | 0.917 |
| **Education year** | 1.064 | (0.875, 1.293) | 0.535 |
| **Female** | 1.566 | (0.486, 5.045) | 0.452 |
| **Left-handedness** | 2.700 | (0.560, 13.027) | 0.216 |
| **Smoker** | 0.824 | (0.176, 3.846) | 0.805 |
| **Alcohol use** | 0.381 | (0.120, 1.210) | 0.102 |
| **Myocardial infarction** | 0.930 | (0.116, 7.442) | 0.945 |
| **Previous stroke** | 0.964 | (0.120, 7.722) | 0.972 |
| **PAD** | 1.574 | (0.193, 12.838) | 0.672 |
| **Carotid artery disease** | 0.868 | (0.109, 6.934) | 0.894 |
| **Hypertension** | 0.989 | (0.313, 3.125) | 0.984 |
| **Diabetes** | 0.355 | (0.045, 2.793) | 0.325 |
| **COPD** | 1.175 | (0.146, 9.469) | 0.880 |
| **Event type (TIA with positive imaging) (a)** | 0.964 | (0.944, 0.984) | 1.000 |
| **TOAST_SVO** | 0.232 | (0.030, 1.820) | 0.164 |
| **NIHSS** | 1.260 | (1.006, 1.579) | **0.045*** |
| **mRS** | 1.094 | (0.617, 1.939) | 0.759 |
| **SBP (mmHg)** | 0.994 | (0.960, 1.029) | 0.715 |
| **DBP (mmHg)** | 0.998 | (0.946, 1.054) | 0.952 |
| **BMI (km/m2)** | 1.049 | (0.947, 1.161) | 0.362 |
| **Haemoglobin (g/dL)** | 0.822 | (0.561, 1.204) | 0.313 |
| **Cholesterol (mmol/L)** | 1.015 | (0.552, 1.864) | 0.963 |
| **Albumin (g/dL)** | 0.964 | (0.833, 1.117) | 0.630 |
| **Creatinine (μmol/L)** | 1.012 | (0.984, 1.041) | 0.418 |
| **eGFR (mL/min)** | 0.978 | (0.948, 1.009) | 0.160 |
| **Fazeka PVHs score** | 0.981 | (0.413, 2.329) | 0.965 |
| **Fazeka DWMH score** | 1.090 | (0.459, 2.589) | 0.844 |
| **Fazeka Total score** | 1.021 | (0.631, 1.651) | 0.934 |
| **Scheltens PVH score** | 0.993 | (0.600, 1.644) | 0.980 |
| **Scheltens WMH score** | 0.978 | (0.853, 1.121) | 0.746 |
| **Scheltens BG score** | 0.931 | (0.568, 1.527) | 0.778 |
| **Scheltens ITF score** | 1.079 | (0.774, 1.503) | 0.655 |
| **Scheltens total score** | 0.992 | (0.907, 1.086) | 0.868 |
| **WMH volume/ICV*100%** | 0.782 | (0.397, 1.540) | 0.476 |
| **Brain volume** | 0.996 | (0.991, 1.002) | 0.220 |
| **CSF volume** | 0.994 | (0.984, 1.004) | 0.268 |
| **Trial intervention (allopurinol)** | 0.765 | (0.238, 2.458) | 0.653 |

**Table S26: Predictors of continuous cognitive improvement over 2 years after stroke (multiple imputed data).**

Logistic regression with no adjustment using multiple imputed data. (a): Fisher’s exact test, using multiple imputed data. The reference group was participants having any other cognitive trajectories. *: *p<*0.05.

## Table S27: Predictors of cognitive decline in the second year after stroke (multiple imputed data).

|  | **Model 1 (MI)** | | |  | **Model 2 (MI)** | | |
| --- | --- | --- | --- | --- | --- | --- | --- |
| **Variable** | **OR** | **95% CI** | ***p* value** |  | **OR** | **95% CI** | ***p* value** |
| **Age** | 1.010 | (0.981, 1.040) | 0.483 |  |  |  |  |
| **Education year** | 0.927 | (0.843, 1.021) | 0.123 |  |  |  |  |
| **Female** | 1.105 | (0.657, 1.859) | 0.708 |  | 1.134 | (0.672, 1.914) | 0.639 |
| **Left-handedness** | 1.230 | (0.498, 3.034) | 0.654 |  | 1.237 | (0.499, 3.066) | 0.646 |
| **Smoker** | 0.874 | (0.464, 1.643) | 0.675 |  | 0.837 | (0.437, 1.605) | 0.593 |
| **Alcohol use** | 1.150 | (0.663, 1.997) | 0.619 |  | 1.175 | (0.675, 2.046) | 0.569 |
| **Myocardial infarction** | 1.105 | (0.477, 2.561) | 0.815 |  | 1.094 | (0.471, 2.543) | 0.834 |
| **Previous stroke** | 1.385 | (0.612, 3.137) | 0.434 |  | 1.356 | (0.594, 3.091) | 0.470 |
| **PAD** | 3.595 | (1.442, 8.962) | **0.006*** |  | 3.481 | (1.392, 8.708) | **0.008*** |
| **Carotid artery disease** | 1.924 | (0.909, 4.076) | 0.087 |  | 1.868 | (0.879, 3.968) | 0.104 |
| **Hypertension** | 0.986 | (0.605, 1.606) | 0.954 |  | 0.927 | (0.563, 1.525) | 0.764 |
| **Diabetes** | 0.835 | (0.445, 1.568) | 0.575 |  | 0.775 | (0.409, 1.470) | 0.435 |
| **COPD** | 0.121 | (0.016, 0.907) | **0.040*** |  | 0.115 | (0.015, 0.865) | **0.036*** |
| **Event type (TIA with positive imaging)** | 0.769 | (0.281, 2.105) | 0.609 |  | 0.724 | (0.264, 1.991) | 0.532 |
| **TOAST_SVO** | 1.789 | (1.063, 3.012) | **0.029*** |  | 1.783 | (1.055, 3.012) | **0.031*** |
| **NIHSS** | 1.020 | (0.885, 1.176) | 0.784 |  | 1.017 | (0.880, 1.174) | 0.821 |
| **mRS** | 1.052 | (0.823, 1.346) | 0.684 |  | 1.045 | (0.816, 1.338) | 0.728 |
| **SBP (mmHg)** | 1.007 | (0.993, 1.021) | 0.349 |  | 1.006 | (0.992, 1.021) | 0.402 |
| **DBP (mmHg)** | 0.999 | (0.977, 1.023) | 0.960 |  | 1.002 | (0.978, 1.025) | 0.899 |
| **BMI (km/m2)** | 1.009 | (0.962, 1.058) | 0.728 |  | 1.006 | (0.959, 1.057) | 0.797 |
| **Haemoglobin (g/dL)** | 0.901 | (0.761, 1.068) | 0.229 |  | 0.896 | (0.753, 1.066) | 0.214 |
| **Cholesterol (mmol/L)** | 1.393 | (1.080, 1.796) | **0.011*** |  | 1.419 | (1.092, 1.844) | **0.009*** |
| **Albumin (g/dL)** | 1.028 | (0.967, 1.093) | 0.376 |  | 1.029 | (0.967, 1.095) | 0.374 |
| **Creatinine (μmol/L)** | 0.997 | (0.984, 1.011) | 0.669 |  | 0.996 | (0.983, 1.010) | 0.594 |
| **eGFR (mL/min)** | 1.007 | (0.995, 1.020) | 0.248 |  | 1.009 | (0.996, 1.022) | 0.175 |
| **Fazeka PVHs score** | 1.529 | (1.087, 2.151) | **0.015*** |  | 1.505 | (1.053, 2.151) | **0.025*** |
| **Fazeka DWMH score** | 1.157 | (0.800, 1.672) | 0.439 |  | 1.099 | (0.756, 1.598) | 0.620 |
| **Fazeka Total score** | 1.200 | (0.986, 1.461) | 0.069 |  | 1.174 | (0.957, 1.439) | 0.123 |
| **Scheltens PVH score** | 1.176 | (0.958, 1.444) | 0.122 |  | 1.152 | (0.930, 1.429) | 0.196 |
| **Scheltens WMH score** | 1.052 | (0.995, 1.112) | 0.072 |  | 1.044 | (0.985, 1.107) | 0.145 |
| **Scheltens BG score** | 1.085 | (0.900, 1.309) | 0.392 |  | 1.065 | (0.880, 1.288) | 0.520 |
| **Scheltens ITF score** | 1.010 | (0.864, 1.180) | 0.900 |  | 0.987 | (0.842, 1.158) | 0.874 |
| **Scheltens total score** | 1.031 | (0.994, 1.070) | 0.098 |  | 1.025 | (0.987, 1.066) | 0.199 |
| **WMH volume/ICV*100%** | 1.330 | (1.088, 1.627) | **0.005*** |  | 1.325 | (1.069, 1.642) | **0.010*** |
| **Brain volume** | 1.001 | (0.999, 1.003) | 0.471 |  | 1.001 | (0.999, 1.004) | 0.331 |
| **CSF volume** | 1.002 | (0.998, 1.006) | 0.358 |  | 1.002 | (0.997, 1.006) | 0.397 |
| **Trial intervention (allopurinol)** | 1.040 | (0.638, 1.696) | 0.874 |  | 1.036 | (0.634, 1.693) | 0.887 |

**Table S27: Predictors of cognitive decline in the second year after stroke (multiple imputed data).**

Model 1 (MI): Logistic regression with no adjustment using multiple imputed data; Model 2 (MI): Logistic regression adjusting for age, education using multiple imputed data. The reference group was participants having any other cognitive trajectories. *: *p<*0.05.

## Table S28: Predictors of delayed cognitive decline in the second year after stroke (multiple imputed data).

|  | **Model 1 (MI)** | | |  | **Model 2 (MI)** | | |
| --- | --- | --- | --- | --- | --- | --- | --- |
| **Variable** | **OR** | **95% CI** | ***p* value** |  | **OR** | **95% CI** | ***p* value** |
| **Age** | 0.998 | (0.969, 1.029) | 0.916 |  |  |  |  |
| **Education year** | 0.941 | (0.854, 1.037) | 0.219 |  |  |  |  |
| **Female** | 1.276 | (0.753, 2.163) | 0.365 |  | 1.305 | (0.768, 2.218) | 0.325 |
| **Left-handedness** | 0.639 | (0.213, 1.912) | 0.423 |  | 0.647 | (0.216, 1.940) | 0.437 |
| **Smoker** | 0.983 | (0.521, 1.855) | 0.957 |  | 0.913 | (0.474, 1.756) | 0.784 |
| **Alcohol use** | 1.008 | (0.578, 1.760) | 0.977 |  | 1.011 | (0.578, 1.769) | 0.969 |
| **Myocardial infarction** | 1.014 | (0.421, 2.439) | 0.976 |  | 0.995 | (0.413, 2.400) | 0.991 |
| **Previous stroke** | 1.542 | (0.679, 3.499) | 0.301 |  | 1.483 | (0.650, 3.384) | 0.349 |
| **PAD** | 3.213 | (1.281, 8.061) | **0.013*** |  | 3.164 | (1.257, 7.963) | **0.014*** |
| **Carotid artery disease** | 1.581 | (0.721, 3.465) | 0.253 |  | 1.544 | (0.703, 3.391) | 0.279 |
| **Hypertension** | 0.923 | (0.559, 1.525) | 0.756 |  | 0.901 | (0.540, 1.501) | 0.688 |
| **Diabetes** | 0.756 | (0.390, 1.464) | 0.407 |  | 0.715 | (0.366, 1.396) | 0.326 |
| **COPD** | 0.134 | (0.018, 1.001) | 0.050 |  | 0.127 | (0.017, 0.951) | **0.045*** |
| **Event type (TIA with positive imaging)** | 0.639 | (0.213, 1.912) | 0.423 |  | 0.616 | (0.205, 1.848) | 0.387 |
| **TOAST_SVO** | 1.664 | (0.975, 2.842) | 0.062 |  | 1.681 | (0.981, 2.879) | 0.059 |
| **NIHSS** | 1.004 | (0.865, 1.164) | 0.961 |  | 0.996 | (0.858, 1.156) | 0.959 |
| **mRS** | 1.021 | (0.792, 1.314) | 0.875 |  | 1.010 | (0.784, 1.302) | 0.938 |
| **SBP (mmHg)** | 1.007 | (0.992, 1.022) | 0.368 |  | 1.007 | (0.992, 1.021) | 0.379 |
| **DBP (mmHg)** | 1.008 | (0.984, 1.032) | 0.517 |  | 1.008 | (0.984, 1.033) | 0.512 |
| **BMI (km/m2)** | 0.995 | (0.947, 1.046) | 0.851 |  | 0.991 | (0.942, 1.042) | 0.716 |
| **Haemoglobin (g/dL)** | 0.870 | (0.731, 1.035) | 0.116 |  | 0.853 | (0.713, 1.019) | 0.080 |
| **Cholesterol (mmol/L)** | 1.473 | (1.135, 1.911) | **0.004*** |  | 1.475 | (1.129, 1.927) | **0.004*** |
| **Albumin (g/dL)** | 1.025 | (0.962, 1.091) | 0.449 |  | 1.022 | (0.959, 1.089) | 0.502 |
| **Creatinine (μmol/L)** | 0.994 | (0.980, 1.008) | 0.419 |  | 0.994 | (0.980, 1.009) | 0.435 |
| **eGFR (mL/min)** | 1.008 | (0.996, 1.021) | 0.197 |  | 1.009 | (0.995, 1.022) | 0.205 |
| **Fazeka PVHs score** | 1.145 | (0.797, 1.646) | 0.463 |  | 1.149 | (0.787, 1.679) | 0.472 |
| **Fazeka DWMH score** | 0.955 | (0.646, 1.410) | 0.815 |  | 0.930 | (0.626, 1.382) | 0.720 |
| **Fazeka Total score** | 1.031 | (0.836, 1.272) | 0.773 |  | 1.023 | (0.824, 1.270) | 0.838 |
| **Scheltens PVH score** | 1.042 | (0.840, 1.293) | 0.709 |  | 1.038 | (0.829, 1.301) | 0.745 |
| **Scheltens WMH score** | 1.015 | (0.959, 1.075) | 0.604 |  | 1.012 | (0.953, 1.075) | 0.698 |
| **Scheltens BG score** | 1.065 | (0.877, 1.292) | 0.526 |  | 1.057 | (0.868, 1.286) | 0.582 |
| **Scheltens ITF score** | 1.009 | (0.860, 1.185) | 0.909 |  | 0.997 | (0.847, 1.173) | 0.972 |
| **Scheltens total score** | 1.011 | (0.973, 1.050) | 0.577 |  | 1.009 | (0.969, 1.050) | 0.677 |
| **WMH volume/ICV*100%** | 1.156 | (0.940, 1.423) | 0.170 |  | 1.169 | (0.938, 1.457) | 0.165 |
| **Brain volume** | 1.000 | (0.998, 1.003) | 0.683 |  | 1.001 | (0.998, 1.003) | 0.668 |
| **CSF volume** | 1.001 | (0.996, 1.005) | 0.723 |  | 1.001 | (0.997, 1.006) | 0.621 |
| **Trial intervention (allopurinol)** | 1.034 | (0.626, 1.708) | 0.895 |  | 1.033 | (0.625, 1.709) | 0.898 |

**Table S28: Predictors of delayed cognitive decline in the second year after stroke (multiple imputed data).**

Model 1 (MI): Logistic regression with no adjustment using multiple imputed data; Model 2 (MI): Logistic regression adjusting for age, education using multiple imputed data. The reference group was participants having any other cognitive trajectories. *: *p<*0.05.

## Table S29: Predictors of continuous cognitive decline over 2 years after stroke (multiple imputed data).

|  | **Model 2 (MI)** | | |
| --- | --- | --- | --- |
| **Variable** | **OR** | **95% CI** | ***p* value** |
| **Ageatrandomisation (a)** | 9.132 | (2.418, 15.846) | **0.008*** |
| **Educationyear (a)** | -1.294 | (-3.497, 0.909) | 0.249 |
| **Female (a)** | 0.976 | (0.957,0.995) | 0.183 |
| **Left-handedness (a)** | 14.391 | (2.750,75.324) | **0.006*** |
| **Smoker (a)** | 0.979 | (0.963,0.996) | 0.601 |
| **Alcohol use (a)** | 1.024 | (1.005, 1.043) | 0.190 |
| **Myocardial infarction (a)** | 2.084 | (0.236, 18.406) | 0.430 |
| **Previous stroke (a)** | 0.982 | (0.967, 0.996) | 1.000 |
| **PAD (a)** | 3.526 | (0.392, 31.705) | 0.292 |
| **Carotid artery disease (a)** | 5.031 | (0.887, 28.545) | 0.102 |
| **Hypertension (a)** | 2.000 | (0.362, 11.059) | 0.685 |
| **Diabetes (a)** | 2.029 | (0.364, 11.299) | 0.345 |
| **COPD (a)** | 0.982 | (0.968, 0.996) | 1.000 |
| **Event type (TIA with positive imaging) (a)** | 2.632 | (0.296, 23.405) | 0.364 |
| **TOAST_SVO (a)** | 2.688 | (0.533, 13.544) | 0.352 |
| **NIHSS (a)** | 0.517 | (-0.849, 1.883) | 0.457 |
| **mRS (a)** | 0.342 | (-0.460, 1.143) | 0.402 |
| **SBP (mmHg) (b)** | 1.386 | (-12.418, 15.189) | 0.844 |
| **DBP (mmHg) (b)** | -9.846 | (-18.407, -1.285) | **0.024*** |
| **BMI (km/m2) (b)** | 3.704 | (-0.363, 7.771) | 0.077 |
| **Haemoglobin (g/dL) (b)** | 0.651 | (-0.513, 1.816) | 0.272 |
| **Cholesterol (mmol/L) (b)** | -0.392 | (-1.154, 0.369) | 0.312 |
| **Albumin (g/dL) (b)** | 0.802 | (-2.424, 4.029) | 0.625 |
| **Creatinine (μmol/L) (b)** | 8.949 | (-5.874, 23.772) | 0.236 |
| **eGFR (mL/min) (b)** | -2.733 | (-18.742, 13.277) | 0.737 |
| **Fazeka PVHs score (b)** | 1.602 | (1.534, 1.669) | **<0.001*** |
| **Fazeka DWMH score (b)** | 0.884 | (0.366, 1.402) | **0.001*** |
| **Fazeka Total score (b)** | 2.486 | (1.562, 3.409) | **<0.001*** |
| **Scheltens PVH score (b)** | 1.856 | (0.946, 2.766) | **<0.001*** |
| **Scheltens WMH score (b)** | 7.647 | (4.230, 11.064) | **<0.001*** |
| **Scheltens BG score (b)** | 0.407 | (-0.607, 1.421) | 0.431 |
| **Scheltens ITF score (b)** | 0.031 | (-1.231, 1.293) | 0.961 |
| **Scheltens total score (b)** | 9.941 | (4.744, 15.138) | **<0.001*** |
| **WMH volume/ICV*100%** | 2.355 | (1.520, 3.189) | **<0.001*** |
| **Brain volume (b)** | 48.930 | (-39.072, 136.932) | 0.276 |
| **CSF volume (b)** | 46.265 | (-0.812, 93.343) | 0.054 |
| **Trial intervention (allopurinol)** | 1.082 | (0.216, 5.436) | 1.000 |

**Table S29: Predictors of continuous cognitive decline over 2 years after stroke (multiple imputed data).**

(a): Fisher’s exact test, using multiple imputed data; (b) T test, using multiple imputed data. The reference group was participants having any other cognitive trajectories. *: *p<*0.05.

## Table S30: Predictors of cognitive impairment in year 1 after stroke (adjusting for allopurinol treatment group using multiple imputed data).

|  | **Model 3 (MI)** | | |  | **Model 4 (MI)** | | |
| --- | --- | --- | --- | --- | --- | --- | --- |
| **Variable** | **OR** | **95% CI** | **p value** |  | **OR** | **95% CI** | **p value** |
| **Age** | 1.046 | (1.016, 1.076) | **0.002*** |  |  |  |  |
| **Education** | 0.897 | (0.815, 0.987) | **0.025*** |  |  |  |  |
| **Female** | 0.822 | (0.491, 1.377) | 0.457 |  | 0.853 | (0.503, 1.444) | 0.553 |
| **Left-handedness** | 2.555 | (1.134, 5.759) | **0.024*** |  | 2.604 | (1.132, 5.987) | **0.024*** |
| **Smoker** | 1.916 | (1.097, 3.345) | **0.022*** |  | 2.275 | (1.250, 4.139) | **0.007*** |
| **Alcohol use** | 0.758 | (0.455, 1.261) | 0.286 |  | 0.792 | (0.472, 1.331) | 0.379 |
| **Myocardial infarction** | 1.097 | (0.487, 2.469) | 0.823 |  | 1.117 | (0.489, 2.554) | 0.793 |
| **Previous stroke** | 1.859 | (0.864, 4.002) | 0.113 |  | 1.986 | (0.903, 4.368) | 0.088 |
| **PAD** | 1.504 | (0.580, 3.903) | 0.401 |  | 1.382 | (0.523, 3.657) | 0.514 |
| **Carotid artery disease** | 0.950 | (0.424, 2.127) | 0.900 |  | 0.894 | (0.393, 2.035) | 0.789 |
| **Hypertension** | 1.419 | (0.884, 2.278) | 0.148 |  | 1.220 | (0.749, 1.988) | 0.425 |
| **Diabetes** | 1.535 | (0.877, 2.688) | 0.134 |  | 1.397 | (0.785, 2.489) | 0.256 |
| **COPD** | 2.589 | (1.149, 5.836) | **0.022*** |  | 2.666 | (1.150, 6.178) | **0.022*** |
| **Event type (TIA with positive imaging)** | 1.290 | (0.540, 3.082) | 0.567 |  | 1.150 | (0.473, 2.792) | 0.758 |
| **TOAST_SVO** | 1.052 | (0.624, 1.776) | 0.848 |  | 1.002 | (0.588, 1.709) | 0.994 |
| **NIHSS** | 1.049 | (0.918, 1.200) | 0.479 |  | 1.064 | (0.925, 1.224) | 0.385 |
| **mRS** | 1.181 | (0.933, 1.494) | 0.167 |  | 1.192 | (0.936, 1.517) | 0.155 |
| **SBP (mmHg)** | 1.009 | (0.995, 1.023) | 0.201 |  | 1.007 | (0.993, 1.021) | 0.327 |
| **DBP (mmHg)** | 0.991 | (0.969, 1.013) | 0.402 |  | 0.998 | (0.976, 1.022) | 0.893 |
| **BMI (km/m2)** | 1.029 | (0.983, 1.076) | 0.225 |  | 1.035 | (0.988, 1.085) | 0.148 |
| **Haemoglobin (g/dL)** | 0.890 | (0.755, 1.048) | 0.163 |  | 0.913 | (0.770, 1.083) | 0.298 |
| **Cholesterol (mmol/L)** | 0.862 | (0.663, 1.122) | 0.270 |  | 0.904 | (0.688, 1.187) | 0.468 |
| **Albumin (g/dL)** | 0.904 | (0.849, 0.963) | **0.002*** |  | 0.905 | (0.849, 0.966) | **0.003*** |
| **Creatinine (μmol/L)** | 1.009 | (0.997, 1.022) | 0.138 |  | 1.006 | (0.993, 1.019) | 0.338 |
| **eGFR (mL/min)** | 0.993 | (0.981, 1.005) | 0.275 |  | 0.998 | (0.985, 1.011) | 0.743 |
| **Fazeka PVHs score** | 1.474 | (1.056, 2.058) | **0.023*** |  | 1.273 | (0.894, 1.813) | 0.180 |
| **Fazeka DWMH score** | 1.525 | (1.075, 2.164) | **0.018*** |  | 1.357 | (0.948, 1.942) | 0.095 |
| **Fazeka Total score** | 1.284 | (1.062, 1.553) | **0.010*** |  | 1.185 | (0.972, 1.444) | 0.094 |
| **Scheltens PVH score** | 1.246 | (1.023, 1.518) | **0.029*** |  | 1.139 | (0.926, 1.402) | 0.217 |
| **Scheltens WMH score** | 1.107 | (1.048, 1.170) | **<0.001*** |  | 1.082 | (1.022, 1.146) | **0.007*** |
| **Scheltens BG score** | 1.141 | (0.954, 1.365) | 0.147 |  | 1.086 | (0.903, 1.307) | 0.382 |
| **Scheltens ITF score** | 1.181 | (1.022, 1.363) | **0.024*** |  | 1.133 | (0.978, 1.313) | 0.096 |
| **Scheltens total score** | 1.067 | (1.030, 1.106) | **<0.001*** |  | 1.050 | (1.012, 1.090) | **0.010*** |
| **WMH volume/ICV** | 1.404 | (1.148, 1.717) | **0.001*** |  | 1.285 | (1.040, 1.587) | **0.020*** |
| **Brain volume** | 0.996 | (0.993, 0.998) | **0.001*** |  | 0.996 | (0.994, 0.999) | **0.005*** |
| **CSF volume** | 1.007 | (1.002, 1.011) | **0.002*** |  | 1.005 | (1.001, 1.010) | **0.016*** |

**Table S30: Predictors of cognitive impairment in year 1 after stroke (adjusting for allopurinol treatment group** **using multiple imputed data).**

Model 3 (MI): Logistic regression with adjustment for allopurinol treatment group using multiple imputed data; Model 4 (MI): Logistic regression adjusting for age, education and allopurinol treatment group using multiple imputed data. The reference group was participants having not cognitive impairment. *: *p<*0.05.

## Table S31: Predictors of cognitive impairment in year 2 after stroke (adjusting for allopurinol treatment group using multiple imputed data).

|  | **Model 3 (MI)** | | |  | **Model 4 (MI)** | | |
| --- | --- | --- | --- | --- | --- | --- | --- |
| **Variable** | **OR** | **95% CI** | **p value** |  | **OR** | **95% CI** | **p value** |
| **Age** | 1.037 | (1.008, 1.067) | **0.012*** |  |  |  |  |
| **Education** | 0.886 | (0.803, 0.977) | **0.015*** |  |  |  |  |
| **Female** | 0.673 | (0.397, 1.143) | 0.143 |  | 0.694 | (0.405, 1.189) | 0.184 |
| **Left-handedness** | 2.204 | (0.973, 4.990) | 0.058 |  | 2.242 | (0.973, 5.167) | 0.058 |
| **Smoker** | 1.278 | (0.718, 2.274) | 0.404 |  | 1.366 | (0.746, 2.502) | 0.313 |
| **Alcohol use** | 1.048 | (0.620, 1.771) | 0.861 |  | 1.104 | (0.647, 1.881) | 0.717 |
| **Myocardial infarction** | 1.544 | (0.714, 3.339) | 0.269 |  | 1.576 | (0.719, 3.451) | 0.256 |
| **Previous stroke** | 2.958 | (1.399, 6.255) | **0.005*** |  | 3.135 | (1.452, 6.772) | **0.004*** |
| **PAD** | 4.685 | (1.850, 11.866) | **0.001*** |  | 4.495 | (1.752, 11.534) | **0.002*** |
| **Carotid artery disease** | 1.845 | (0.880, 3.869) | 0.105 |  | 1.775 | (0.836, 3.771) | 0.135 |
| **Hypertension** | 1.472 | (0.916, 2.367) | 0.110 |  | 1.296 | (0.796, 2.112) | 0.297 |
| **Diabetes** | 1.559 | (0.891, 2.728) | 0.120 |  | 1.413 | (0.795, 2.509) | 0.238 |
| **COPD** | 1.279 | (0.536, 3.048) | 0.579 |  | 1.243 | (0.513, 3.015) | 0.630 |
| **Event type (TIA with positive imaging)** | 1.292 | (0.542, 3.082) | 0.563 |  | 1.161 | (0.481, 2.802) | 0.739 |
| **TOAST_SVO** | 1.527 | (0.918, 2.541) | 0.103 |  | 1.487 | (0.886, 2.497) | 0.133 |
| **NIHSS** | 1.015 | (0.885, 1.165) | 0.829 |  | 1.020 | (0.884, 1.177) | 0.786 |
| **mRS** | 1.122 | (0.886, 1.420) | 0.340 |  | 1.123 | (0.882, 1.429) | 0.347 |
| **SBP (mmHg)** | 0.998 | (0.985, 1.012) | 0.818 |  | 0.996 | (0.982, 1.010) | 0.612 |
| **DBP (mmHg)** | 0.967 | (0.944, 0.989) | **0.004*** |  | 0.972 | (0.949, 0.995) | **0.018*** |
| **BMI (km/m2)** | 1.037 | (0.991, 1.085) | 0.118 |  | 1.041 | (0.993, 1.091) | 0.093 |
| **Haemoglobin (g/dL)** | 0.951 | (0.808, 1.120) | 0.550 |  | 0.970 | (0.819, 1.150) | 0.729 |
| **Cholesterol (mmol/L)** | 1.013 | (0.785, 1.306) | 0.922 |  | 1.054 | (0.810, 1.372) | 0.695 |
| **Albumin (g/dL)** | 0.984 | (0.927, 1.044) | 0.584 |  | 0.987 | (0.929, 1.050) | 0.684 |
| **Creatinine (μmol/L)** | 1.009 | (0.997, 1.022) | 0.145 |  | 1.007 | (0.994, 1.020) | 0.292 |
| **eGFR (mL/min)** | 0.997 | (0.985, 1.009) | 0.662 |  | 1.001 | (0.989, 1.014) | 0.850 |
| **Fazeka PVHs score** | 1.620 | (1.162, 2.257) | **0.004*** |  | 1.453 | (1.025, 2.061) | **0.036*** |
| **Fazeka DWMH score** | 1.536 | (1.082, 2.180) | **0.016*** |  | 1.381 | (0.966, 1.973) | 0.076 |
| **Fazeka Total score** | 1.328 | (1.098, 1.605) | **0.003*** |  | 1.242 | (1.020, 1.513) | **0.031*** |
| **Scheltens PVH score** | 1.319 | (1.083, 1.607) | **0.006*** |  | 1.232 | (1.002, 1.514) | **0.048*** |
| **Scheltens WMH score** | 1.093 | (1.036, 1.155) | **0.001*** |  | 1.071 | (1.012, 1.133) | **0.018*** |
| **Scheltens BG score** | 1.091 | (0.910, 1.309) | 0.345 |  | 1.041 | (0.863, 1.255) | 0.677 |
| **Scheltens ITF score** | 1.119 | (0.970, 1.292) | 0.124 |  | 1.074 | (0.927, 1.244) | 0.344 |
| **Scheltens total score** | 1.058 | (1.022, 1.097) | **0.002*** |  | 1.043 | (1.005, 1.083) | **0.026*** |
| **WMH volume/ICV** | 1.466 | (1.197, 1.796) | **<0.001*** |  | 1.374 | (1.110, 1.701) | **0.004*** |
| **Brain volume** | 0.999 | (0.997, 1.001) | 0.515 |  | 1.000 | (0.998, 1.002) | 0.972 |
| **CSF volume** | 1.010 | (1.005, 1.014) | **<0.001*** |  | 1.009 | (1.005, 1.014) | **<0.001*** |

**Table S31: Predictors of cognitive impairment in year 2 after stroke (adjusting for allopurinol treatment group** **using multiple imputed data).**

Model 3 (MI): Logistic regression with adjustment for allopurinol treatment group using multiple imputed data; Model 4 (MI): Logistic regression adjusting for age, education and allopurinol treatment group using multiple imputed data. The reference group was participants having not cognitive impairment. *: *p<*0.05.

## Table S32: Predictors of cognitive improvement in the second year after stroke (adjusting for allopurinol treatment group using multiple imputed data).

|  | **Model 3 (MI)** | | |  | **Model 4 (MI)** | | |
| --- | --- | --- | --- | --- | --- | --- | --- |
| **Variable** | **OR** | **95% CI** | **p value** |  | **OR** | **95% CI** | **p value** |
| **Age** | 1.019 | (0.988, 1.050) | 0.231 |  |  |  |  |
| **Education** | 0.995 | (0.908, 1.092) | 0.923 |  |  |  |  |
| **Female** | 1.640 | (0.972, 2.769) | 0.064 |  | 1.648 | (0.974, 2.789) | 0.063 |
| **Left-handedness** | 0.855 | (0.311, 2.351) | 0.762 |  | 0.838 | (0.304, 2.309) | 0.733 |
| **Smoker** | 2.791 | (1.574, 4.948) | **<0.001*** |  | 3.345 | (1.814, 6.167) | **<0.001*** |
| **Alcohol use** | 0.669 | (0.391, 1.146) | 0.143 |  | 0.682 | (0.398, 1.170) | 0.165 |
| **Myocardial infarction** | 0.656 | (0.244, 1.766) | 0.404 |  | 0.664 | (0.246, 1.790) | 0.418 |
| **Previous stroke** | 1.303 | (0.558, 3.043) | 0.541 |  | 1.361 | (0.579, 3.201) | 0.480 |
| **PAD** | 1.221 | (0.429, 3.477) | 0.709 |  | 1.190 | (0.416, 3.405) | 0.745 |
| **Carotid artery disease** | 0.443 | (0.150, 1.304) | 0.139 |  | 0.438 | (0.148, 1.292) | 0.135 |
| **Hypertension** | 1.733 | (1.036, 2.899) | **0.036*** |  | 1.672 | (0.990, 2.824) | 0.055 |
| **Diabetes** | 0.772 | (0.398, 1.497) | 0.444 |  | 0.756 | (0.388, 1.475) | 0.412 |
| **COPD** | 2.065 | (0.881, 4.840) | 0.095 |  | 2.125 | (0.900, 5.020) | 0.086 |
| **Event type (TIA with positive imaging)** | 0.877 | (0.319, 2.410) | 0.799 |  | 0.852 | (0.309, 2.351) | 0.757 |
| **TOAST_SVO** | 0.899 | (0.507, 1.594) | 0.717 |  | 0.875 | (0.492, 1.556) | 0.650 |
| **NIHSS** | 1.036 | (0.897, 1.197) | 0.627 |  | 1.046 | (0.904, 1.211) | 0.545 |
| **mRS** | 1.042 | (0.809, 1.343) | 0.748 |  | 1.049 | (0.813, 1.354) | 0.711 |
| **SBP (mmHg)** | 1.001 | (0.986, 1.016) | 0.932 |  | 1.000 | (0.985, 1.015) | 0.980 |
| **DBP (mmHg)** | 1.006 | (0.982, 1.030) | 0.643 |  | 1.009 | (0.985, 1.034) | 0.461 |
| **BMI (km/m2)** | 1.018 | (0.970, 1.069) | 0.463 |  | 1.024 | (0.974, 1.076) | 0.360 |
| **Haemoglobin (g/dL)** | 0.967 | (0.811, 1.152) | 0.704 |  | 0.986 | (0.823, 1.180) | 0.875 |
| **Cholesterol (mmol/L)** | 0.769 | (0.575, 1.031) | 0.079 |  | 0.786 | (0.583, 1.060) | 0.114 |
| **Albumin (g/dL)** | 0.915 | (0.856, 0.978) | **0.009*** |  | 0.918 | (0.858, 0.982) | **0.013*** |
| **Creatinine (μmol/L)** | 0.990 | (0.976, 1.005) | 0.186 |  | 0.988 | (0.974, 1.003) | 0.123 |
| **eGFR (mL/min)** | 1.000 | (0.987, 1.013) | 0.982 |  | 1.003 | (0.989, 1.016) | 0.711 |
| **Fazeka PVHs score** | 1.252 | (0.874, 1.793) | 0.221 |  | 1.194 | (0.819, 1.738) | 0.357 |
| **Fazeka DWMH score** | 1.131 | (0.773, 1.655) | 0.526 |  | 1.093 | (0.740, 1.616) | 0.654 |
| **Fazeka Total score** | 1.116 | (0.908, 1.370) | 0.297 |  | 1.088 | (0.878, 1.347) | 0.442 |
| **Scheltens PVH score** | 1.091 | (0.881, 1.352) | 0.425 |  | 1.057 | (0.846, 1.322) | 0.624 |
| **Scheltens WMH score** | 1.044 | (0.985, 1.105) | 0.146 |  | 1.037 | (0.976, 1.102) | 0.238 |
| **Scheltens BG score** | 1.309 | (1.087, 1.576) | **0.005*** |  | 1.298 | (1.075, 1.567) | **0.007*** |
| **Scheltens ITF score** | 1.061 | (0.909, 1.239) | 0.451 |  | 1.050 | (0.897, 1.230) | 0.544 |
| **Scheltens total score** | 1.036 | (0.998, 1.075) | 0.067 |  | 1.032 | (0.992, 1.074) | 0.117 |
| **WMH volume/ICV** | 1.067 | (0.859, 1.325) | 0.558 |  | 1.024 | (0.812, 1.292) | 0.840 |
| **Brain volume** | 0.996 | (0.993, 0.998) | **0.001*** |  | 0.996 | (0.993, 0.998) | **0.002*** |
| **CSF volume** | 0.997 | (0.993, 1.002) | 0.245 |  | 0.996 | (0.991, 1.001) | 0.110 |

**Table S32: Predictors of cognitive improvement in the second year after stroke (adjusting for allopurinol treatment group** **using multiple imputed data).**

Model 3 (MI): Logistic regression with adjustment for allopurinol treatment group using multiple imputed data; Model 4 (MI): Logistic regression adjusting for age, education and allopurinol treatment group using multiple imputed data. The reference group was participants having not cognitive impairment. *: *p<*0.05.

## Table S33: Predictors of cognitive improvement then stable cognition after stroke (adjusting for allopurinol treatment group using multiple imputed data).

|  | **Model 3 (MI)** | | |  | **Model 4 (MI)** | | |
| --- | --- | --- | --- | --- | --- | --- | --- |
| **Variable** | **OR** | **95% CI** | **p value** |  | **OR** | **95% CI** | **p value** |
| **Age** | 0.985 | (0.955, 1.017) | 0.366 |  |  |  |  |
| **Education** | 1.015 | (0.923, 1.116) | 0.761 |  |  |  |  |
| **Female** | 0.700 | (0.388, 1.263) | 0.237 |  | 0.698 | (0.387, 1.259) | 0.232 |
| **Left-handedness** | 0.983 | (0.357, 2.705) | 0.973 |  | 0.997 | (0.361, 2.751) | 0.996 |
| **Smoker** | 0.401 | (0.175, 0.918) | **0.031*** |  | 0.364 | (0.156, 0.849) | **0.019*** |
| **Alcohol use** | 0.986 | (0.553, 1.758) | 0.962 |  | 0.964 | (0.539, 1.724) | 0.902 |
| **Myocardial infarction** | 1.712 | (0.754, 3.884) | 0.198 |  | 1.708 | (0.751, 3.881) | 0.201 |
| **Previous stroke** | 1.230 | (0.507, 2.982) | 0.647 |  | 1.209 | (0.496, 2.948) | 0.676 |
| **PAD** | 0.206 | (0.027, 1.563) | 0.126 |  | 0.211 | (0.028, 1.603) | 0.132 |
| **Carotid artery disease** | 0.869 | (0.344, 2.198) | 0.767 |  | 0.881 | (0.348, 2.233) | 0.790 |
| **Hypertension** | 0.985 | (0.584, 1.660) | 0.954 |  | 1.035 | (0.607, 1.764) | 0.900 |
| **Diabetes** | 0.613 | (0.297, 1.267) | 0.187 |  | 0.622 | (0.300, 1.292) | 0.203 |
| **COPD** | 0.984 | (0.358, 2.709) | 0.976 |  | 0.977 | (0.353, 2.702) | 0.964 |
| **Event type (TIA with positive imaging)** | 0.521 | (0.152, 1.788) | 0.300 |  | 0.535 | (0.155, 1.841) | 0.321 |
| **TOAST_SVO** | 0.602 | (0.318, 1.139) | 0.119 |  | 0.612 | (0.323, 1.161) | 0.133 |
| **NIHSS** | 0.838 | (0.690, 1.016) | 0.073 |  | 0.834 | (0.688, 1.012) | 0.065 |
| **mRS** | 0.764 | (0.579, 1.009) | 0.058 |  | 0.761 | (0.576, 1.005) | 0.054 |
| **SBP (mmHg)** | 0.992 | (0.976, 1.007) | 0.290 |  | 0.992 | (0.977, 1.008) | 0.331 |
| **DBP (mmHg)** | 1.005 | (0.981, 1.030) | 0.670 |  | 1.003 | (0.978, 1.029) | 0.812 |
| **BMI (km/m2)** | 1.010 | (0.960, 1.062) | 0.714 |  | 1.008 | (0.957, 1.061) | 0.773 |
| **Haemoglobin (g/dL)** | 1.176 | (0.974, 1.418) | 0.091 |  | 1.168 | (0.964, 1.416) | 0.113 |
| **Cholesterol (mmol/L)** | 1.011 | (0.759, 1.346) | 0.941 |  | 0.989 | (0.737, 1.328) | 0.942 |
| **Albumin (g/dL)** | 1.004 | (0.940, 1.072) | 0.908 |  | 1.000 | (0.936, 1.069) | 0.990 |
| **Creatinine (μmol/L)** | 1.001 | (0.987, 1.016) | 0.867 |  | 1.002 | (0.988, 1.017) | 0.742 |
| **eGFR (mL/min)** | 1.001 | (0.988, 1.014) | 0.886 |  | 0.999 | (0.986, 1.013) | 0.917 |
| **Fazeka PVHs score** | 0.577 | (0.360, 0.925) | **0.022*** |  | 0.588 | (0.361, 0.956) | **0.032*** |
| **Fazeka DWMH score** | 0.700 | (0.451, 1.086) | 0.112 |  | 0.718 | (0.459, 1.121) | 0.145 |
| **Fazeka Total score** | 0.751 | (0.580, 0.973) | **0.030*** |  | 0.760 | (0.583, 0.991) | **0.043*** |
| **Scheltens PVH score** | 0.773 | (0.599, 0.997) | **0.048*** |  | 0.783 | (0.602, 1.019) | 0.069 |
| **Scheltens WMH score** | 0.912 | (0.853, 0.976) | **0.007*** |  | 0.913 | (0.851, 0.979) | **0.011*** |
| **Scheltens BG score** | 0.937 | (0.754, 1.166) | 0.562 |  | 0.951 | (0.763, 1.185) | 0.655 |
| **Scheltens ITF score** | 0.998 | (0.843, 1.181) | 0.979 |  | 1.011 | (0.853, 1.199) | 0.897 |
| **Scheltens total score** | 0.951 | (0.910, 0.994) | **0.025*** |  | 0.952 | (0.909, 0.997) | **0.038*** |
| **WMH volume/ICV** | 0.675 | (0.490, 0.931) | **0.017*** |  | 0.676 | (0.481, 0.949) | **0.024*** |
| **Brain volume** | 1.000 | (0.997, 1.002) | 0.840 |  | 0.999 | (0.997, 1.002) | 0.642 |
| **CSF volume** | 1.000 | (0.996, 1.005) | 0.924 |  | 1.001 | (0.996, 1.006) | 0.710 |

**Table S33: Predictors of cognitive improvement then stable cognition after stroke (adjusting for allopurinol treatment group** **using multiple imputed data).**

Model 3 (MI): Logistic regression with adjustment for allopurinol treatment group using multiple imputed data; Model 4 (MI): Logistic regression adjusting for age, education and allopurinol treatment group using multiple imputed data. The reference group was participants having not cognitive impairment. *: *p<*0.05.

## Table S34: Predictors of cognitive decline in the second year after stroke (adjusting for allopurinol treatment group using multiple imputed data).

|  | **Model 3 (MI)** | | |  | **Model 4 (MI)** | | |
| --- | --- | --- | --- | --- | --- | --- | --- |
| **Variable** | **OR** | **95% CI** | **p value** |  | **OR** | **95% CI** | **p value** |
| **Age** | 1.010 | (0.981, 1.040) | 0.484 |  |  |  |  |
| **Education** | 0.927 | (0.843, 1.021) | 0.123 |  |  |  |  |
| **Female** | 1.103 | (0.655, 1.857) | 0.712 |  | 1.132 | (0.670, 1.912) | 0.643 |
| **Left-handedness** | 1.233 | (0.499, 3.044) | 0.650 |  | 1.240 | (0.500, 3.075) | 0.642 |
| **Smoker** | 0.873 | (0.464, 1.641) | 0.673 |  | 0.837 | (0.437, 1.604) | 0.591 |
| **Alcohol use** | 1.150 | (0.662, 1.996) | 0.620 |  | 1.175 | (0.674, 2.046) | 0.570 |
| **Myocardial infarction** | 1.106 | (0.477, 2.562) | 0.814 |  | 1.095 | (0.471, 2.545) | 0.832 |
| **Previous stroke** | 1.387 | (0.613, 3.142) | 0.432 |  | 1.357 | (0.595, 3.095) | 0.468 |
| **PAD** | 3.617 | (1.449, 9.031) | **0.006*** |  | 3.504 | (1.399, 8.772) | **0.007*** |
| **Carotid artery disease** | 1.953 | (0.917, 4.160) | 0.083 |  | 1.896 | (0.887, 4.050) | 0.099 |
| **Hypertension** | 0.987 | (0.605, 1.609) | 0.958 |  | 0.928 | (0.563, 1.527) | 0.768 |
| **Diabetes** | 0.835 | (0.445, 1.568) | 0.575 |  | 0.775 | (0.409, 1.469) | 0.435 |
| **COPD** | 0.121 | (0.016, 0.907) | **0.040*** |  | 0.115 | (0.015, 0.865) | **0.036*** |
| **Event type (TIA with positive imaging)** | 0.766 | (0.280, 2.100) | 0.605 |  | 0.723 | (0.263, 1.988) | 0.530 |
| **TOAST_SVO** | 1.793 | (1.065, 3.020) | **0.028*** |  | 1.785 | (1.056, 3.017) | **0.030*** |
| **NIHSS** | 1.021 | (0.885, 1.177) | 0.777 |  | 1.017 | (0.881, 1.176) | 0.814 |
| **mRS** | 1.053 | (0.823, 1.346) | 0.682 |  | 1.045 | (0.816, 1.339) | 0.726 |
| **SBP (mmHg)** | 1.007 | (0.993, 1.021) | 0.348 |  | 1.006 | (0.992, 1.021) | 0.401 |
| **DBP (mmHg)** | 1.000 | (0.977, 1.023) | 0.967 |  | 1.002 | (0.978, 1.025) | 0.892 |
| **BMI (km/m2)** | 1.009 | (0.962, 1.058) | 0.720 |  | 1.007 | (0.959, 1.057) | 0.790 |
| **Haemoglobin (g/dL)** | 0.900 | (0.760, 1.067) | 0.225 |  | 0.895 | (0.752, 1.065) | 0.211 |
| **Cholesterol (mmol/L)** | 1.395 | (1.081, 1.800) | **0.010*** |  | 1.421 | (1.093, 1.847) | **0.009*** |
| **Albumin (g/dL)** | 1.028 | (0.967, 1.093) | 0.379 |  | 1.028 | (0.966, 1.095) | 0.376 |
| **Creatinine (μmol/L)** | 0.997 | (0.984, 1.011) | 0.669 |  | 0.996 | (0.983, 1.010) | 0.595 |
| **eGFR (mL/min)** | 1.007 | (0.995, 1.020) | 0.247 |  | 1.009 | (0.996, 1.022) | 0.174 |
| **Fazeka PVHs score** | 1.529 | (1.087, 2.151) | **0.015*** |  | 1.504 | (1.053, 2.150) | **0.025*** |
| **Fazeka DWMH score** | 1.156 | (0.800, 1.671) | 0.440 |  | 1.099 | (0.756, 1.598) | 0.622 |
| **Fazeka Total score** | 1.200 | (0.985, 1.461) | 0.070 |  | 1.173 | (0.957, 1.439) | 0.124 |
| **Scheltens PVH score** | 1.176 | (0.958, 1.444) | 0.122 |  | 1.152 | (0.929, 1.429) | 0.196 |
| **Scheltens WMH score** | 1.052 | (0.995, 1.112) | 0.073 |  | 1.044 | (0.985, 1.107) | 0.147 |
| **Scheltens BG score** | 1.087 | (0.900, 1.313) | 0.385 |  | 1.066 | (0.880, 1.291) | 0.511 |
| **Scheltens ITF score** | 1.011 | (0.865, 1.182) | 0.893 |  | 0.988 | (0.842, 1.159) | 0.881 |
| **Scheltens total score** | 1.031 | (0.994, 1.070) | 0.098 |  | 1.025 | (0.987, 1.066) | 0.199 |
| **WMH volume/ICV** | 1.330 | (1.088, 1.627) | **0.005*** |  | 1.325 | (1.069, 1.641) | **0.010*** |
| **Brain volume** | 1.001 | (0.999, 1.003) | 0.472 |  | 1.001 | (0.999, 1.004) | 0.332 |
| **CSF volume** | 1.002 | (0.998, 1.006) | 0.355 |  | 1.002 | (0.997, 1.006) | 0.394 |

**Table S34: Predictors of cognitive decline in the second year after stroke (adjusting for allopurinol treatment group** **using multiple imputed data).**

Model 3 (MI): Logistic regression with adjustment for allopurinol treatment group using multiple imputed data; Model 4 (MI): Logistic regression adjusting for age, education and allopurinol treatment group using multiple imputed data. The reference group was participants having not cognitive impairment. *: *p<*0.05.

## Table S35: Predictors of delayed cognitive decline after stroke (adjusting for allopurinol treatment group using multiple imputed data).

|  | **Model 3 (MI)** | | |  | **Model 4 (MI)** | | |
| --- | --- | --- | --- | --- | --- | --- | --- |
| **Variable** | **OR** | **95% CI** | **p value** |  | **OR** | **95% CI** | **p value** |
| **Age** | 0.998 | (0.969, 1.029) | 0.915 |  |  |  |  |
| **Education** | 0.941 | (0.854, 1.037) | 0.219 |  |  |  |  |
| **Female** | 1.275 | (0.752, 2.162) | 0.367 |  | 1.304 | (0.767, 2.216) | 0.327 |
| **Left-handedness** | 0.640 | (0.214, 1.916) | 0.425 |  | 0.648 | (0.216, 1.943) | 0.438 |
| **Smoker** | 0.982 | (0.520, 1.854) | 0.955 |  | 0.912 | (0.474, 1.755) | 0.782 |
| **Alcohol use** | 1.008 | (0.577, 1.759) | 0.979 |  | 1.011 | (0.578, 1.769) | 0.970 |
| **Myocardial infarction** | 1.014 | (0.421, 2.441) | 0.975 |  | 0.996 | (0.413, 2.402) | 0.993 |
| **Previous stroke** | 1.543 | (0.680, 3.504) | 0.299 |  | 1.484 | (0.650, 3.389) | 0.348 |
| **PAD** | 3.231 | (1.286, 8.116) | **0.013*** |  | 3.182 | (1.263, 8.018) | **0.014*** |
| **Carotid artery disease** | 1.599 | (0.726, 3.522) | 0.244 |  | 1.562 | (0.707, 3.448) | 0.270 |
| **Hypertension** | 0.924 | (0.560, 1.527) | 0.759 |  | 0.901 | (0.541, 1.503) | 0.691 |
| **Diabetes** | 0.756 | (0.390, 1.464) | 0.406 |  | 0.715 | (0.366, 1.396) | 0.325 |
| **COPD** | 0.134 | (0.018, 1.002) | 0.050 |  | 0.127 | (0.017, 0.952) | **0.045*** |
| **Event type (TIA with positive imaging)** | 0.637 | (0.213, 1.908) | 0.421 |  | 0.614 | (0.205, 1.845) | 0.385 |
| **TOAST_SVO** | 1.667 | (0.976, 2.848) | 0.061 |  | 1.683 | (0.982, 2.884) | 0.058 |
| **NIHSS** | 1.004 | (0.866, 1.165) | 0.954 |  | 0.997 | (0.859, 1.157) | 0.965 |
| **mRS** | 1.021 | (0.792, 1.315) | 0.874 |  | 1.010 | (0.784, 1.303) | 0.936 |
| **SBP (mmHg)** | 1.007 | (0.992, 1.022) | 0.367 |  | 1.007 | (0.992, 1.021) | 0.378 |
| **DBP (mmHg)** | 1.008 | (0.984, 1.032) | 0.511 |  | 1.008 | (0.984, 1.033) | 0.505 |
| **BMI (km/m2)** | 0.995 | (0.947, 1.046) | 0.857 |  | 0.991 | (0.942, 1.042) | 0.721 |
| **Haemoglobin (g/dL)** | 0.869 | (0.730, 1.034) | 0.114 |  | 0.852 | (0.712, 1.019) | 0.079 |
| **Cholesterol (mmol/L)** | 1.475 | (1.136, 1.915) | **0.004*** |  | 1.477 | (1.130, 1.930) | **0.004*** |
| **Albumin (g/dL)** | 1.024 | (0.962, 1.091) | 0.452 |  | 1.022 | (0.959, 1.089) | 0.505 |
| **Creatinine (μmol/L)** | 0.994 | (0.980, 1.008) | 0.420 |  | 0.994 | (0.980, 1.009) | 0.436 |
| **eGFR (mL/min)** | 1.008 | (0.996, 1.021) | 0.196 |  | 1.009 | (0.995, 1.022) | 0.204 |
| **Fazeka PVHs score** | 1.145 | (0.796, 1.645) | 0.466 |  | 1.149 | (0.786, 1.678) | 0.474 |
| **Fazeka DWMH score** | 0.954 | (0.645, 1.410) | 0.813 |  | 0.930 | (0.626, 1.381) | 0.718 |
| **Fazeka Total score** | 1.031 | (0.836, 1.272) | 0.776 |  | 1.022 | (0.823, 1.270) | 0.841 |
| **Scheltens PVH score** | 1.042 | (0.839, 1.293) | 0.710 |  | 1.038 | (0.828, 1.301) | 0.746 |
| **Scheltens WMH score** | 1.015 | (0.958, 1.075) | 0.610 |  | 1.012 | (0.952, 1.075) | 0.704 |
| **Scheltens BG score** | 1.066 | (0.878, 1.295) | 0.519 |  | 1.058 | (0.869, 1.289) | 0.573 |
| **Scheltens ITF score** | 1.010 | (0.860, 1.186) | 0.903 |  | 0.998 | (0.847, 1.175) | 0.978 |
| **Scheltens total score** | 1.011 | (0.973, 1.050) | 0.579 |  | 1.008 | (0.969, 1.050) | 0.679 |
| **WMH volume/ICV** | 1.156 | (0.939, 1.423) | 0.170 |  | 1.169 | (0.938, 1.456) | 0.165 |
| **Brain volume** | 1.000 | (0.998, 1.003) | 0.684 |  | 1.001 | (0.998, 1.003) | 0.669 |
| **CSF volume** | 1.001 | (0.996, 1.005) | 0.720 |  | 1.001 | (0.997, 1.006) | 0.617 |

**Table S35: Predictors of cognitive decline in the second year after stroke (adjusting for allopurinol treatment group** **using multiple imputed data).**

Model 3 (MI): Logistic regression with adjustment for allopurinol treatment group using multiple imputed data; Model 4 (MI): Logistic regression adjusting for age, education and allopurinol treatment group using multiple imputed data. The reference group was participants having not cognitive impairment. *: *p<*0.05.

## Figure S1: Cognition and cognitive change over 2 years.

**Figure S1: Patient number on specific MoCA score over 2 years.**

(I) MoCA score at baseline and year 1. (II) MoCA score at year 1 and year 2. The numbers represent the number of participants. “a” cells (red) a 2 or more point decrease, “b” cells (green) are the number of participants with a 2 or more-point increase, “c” cells (grey) a 1 point change or no change. “d” cells (blue) show the number of participants with that MoCA score at the specific time point. The more patients there are in the cell, the darker the cell colour is as illustrated in bottom panel.

## Figure S2: Cognitive trajectory over 2 years.


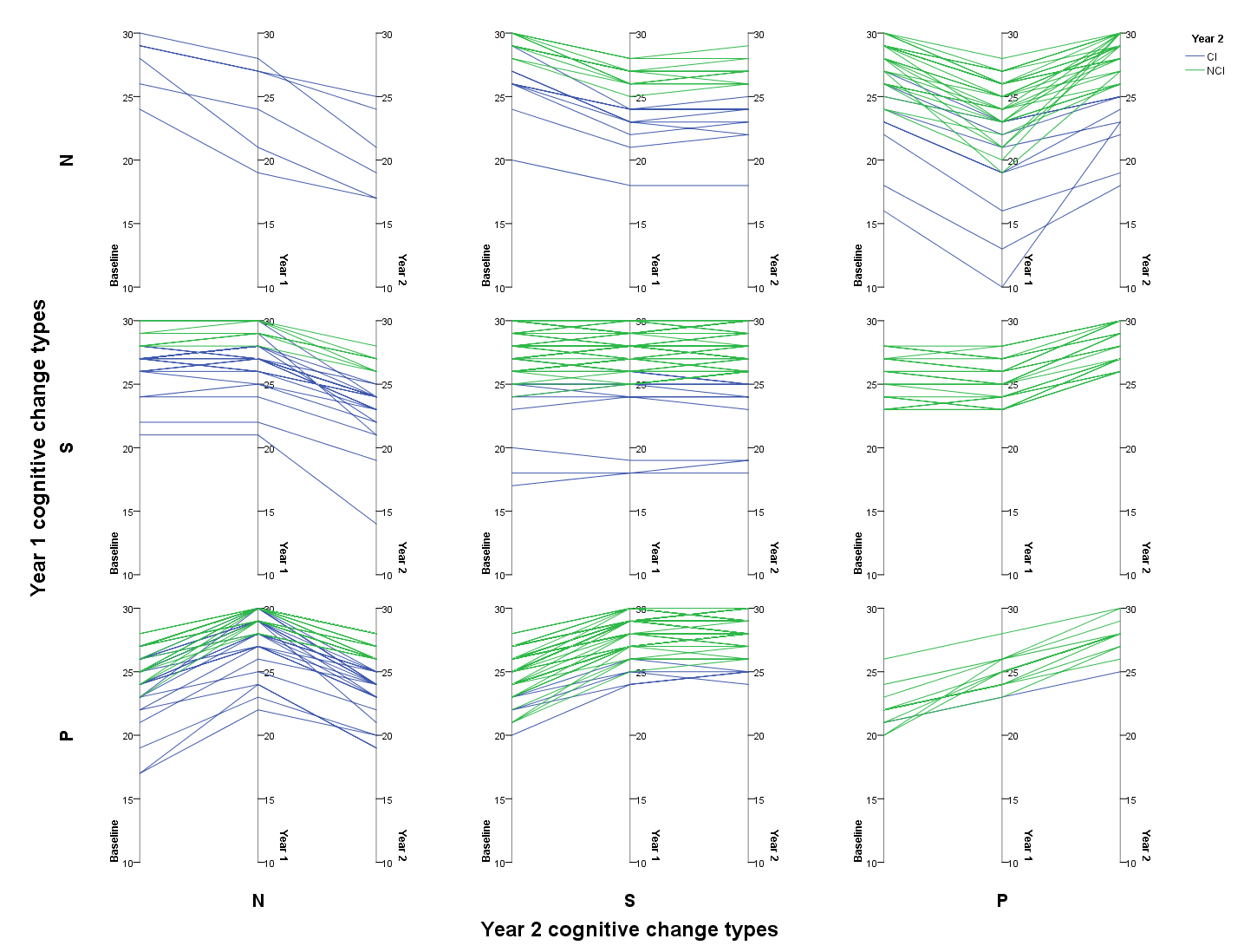


**Figure S2: Cognitive trajectory over 2 years.**

CI: cognitive impairment (MoCA score<26); NCI: non-cognitive impairment (MoCA score≥26); N: Negative cognitive change (2 or more MoCA score decrease); S: Stable cognition (cognitive change between -1 to 1 MoCA); P: Positive cognitive change (2 or more MoCA score increase). Nine different cognitive trajectories were identified. Only a small proportion of participants had continuous cognitive decline or improvement over 2 years after stroke. All the participants having continuous cognitive decline in this study had cognitive impairment at year 2. All the participants having stable cognition in year 1 and then cognitive improvement in year 2 and nearly all the participants having continuous cognitive improvement had no cognitive impairment at year 2. Among participants who experienced the other 6 cognitive trajectories, those having second-year cognitive decline slightly tend to have cognitive impairment at year 2, while those having stable or improved cognition in the second year tend to have non-cognitive impairment at year 2.

## Figure S3: Patterns of cognitive change over 2 years in participants having ischemic stroke and in participants having TIA with positive imaging.

**Figure S3: Patterns of cognitive change over 2 years in participants having ischemic stroke and in participants having TIA with positive imaging.**

(I) Patterns of cognitive change in participants having ischemic stroke. (II) Patterns of cognitive change in participants having TIA with positive imaging. For patterns of cognitive change, the first letter denotes change in year 1 and the second change in year 2 (N: Negative cognitive change; S: Stable cognition; P: Positive cognitive change). For example, S.P denotes stable cognition in year 1 and a 2 point or more improvement in MoCA score in year 2.
